# SEC Mediates m^6^A Deposition and Transcription Pause Release to Drive Cell Identity Transition

**DOI:** 10.1002/advs.78131

**Published:** 2026-10-06

**Authors:** Zhijing Zhang, Jingyi Xu, Yonghong Wu, Yitong Qu, Xinglin Hu, Yuchen Sun, Yanshuang Wu, Zhonghua Liu, Jiaqiang Wang, Lei Lei

**Affiliations:** ^1^ Key Laboratory of Animal Cellular and Genetic Engineering of Heilongjiang Province Northeast Agricultural University Harbin Heilongjiang China; ^2^ Department of Histology and Embryology School of Basic Medical Sciences Harbin Medical University Harbin Heilongjiang China; ^3^ Key Laboratory of Preservation of Human Genetic Resources and Disease Control in China (Harbin Medical University) Ministry of Education Harbin China

**Keywords:** cell identity transition, iPSCs, m^6^A modification, SEC, transcription pause release

## Abstract

**Background**: Induced pluripotent stem cell (iPSC) reprogramming and embryonic stem cell (ESC) differentiation represent opposite models of cell identity transition, yet their regulatory mechanisms remain incompletely understood. Here, we identify the Super Elongation Complex (SEC), the most pivotal regulators of transcription pause release, as a central driver of cell identity transition in both models.

**Results**: SEC depletion markedly impairs iPSC reprogramming efficiency, particularly during the early stage, by suppressing core reprogramming genes, including Klf4 and Myc, thereby disrupting key reprogramming events, such as cell‐cycle progression and activation of the pluripotency network. Mechanistically, SEC activates the expression of Klf4 and Myc by promoting the release of pre‐established paused RNA polymerase II (RNA Pol II). Moreover, SEC also cooperates with METTL3 to facilitate N6‐methyladenosine (m^6^A) deposition and transcription pause release at Klf4 and Myc, revealing an additional regulatory layer during iPSC reprogramming. Similarly, during ESC neural induction, SEC upregulates neural gene expression and facilitates ESC transition into the neuroectoderm lineage, via promoting transcription pause release at relevant loci.

**Conclusions**: These findings establish SEC as a shared regulator of cell identity transition in both iPSC reprogramming and ESC differentiation. Elucidating the mechanisms by which SEC drives cell identity transitions provides a foundation for further investigation into diverse cell identity transitions under physiological and pathological conditions.

## Graphical abstract

The SEC promotes cell identity transitions by facilitating transcription pause release at key identity‐associated genes, including Klf4 and Myc during iPSC reprogramming and Nes and Tubb3 during ESC‐to‐neuroectodermal differentiation. During early iPSC reprogramming, this function additionally involves cooperation with METTL3‐associated m^6^A regulation. SEC disruption impairs these transcriptional programs, thereby compromising the efficient acquisition of pluripotent and neural cell identities.

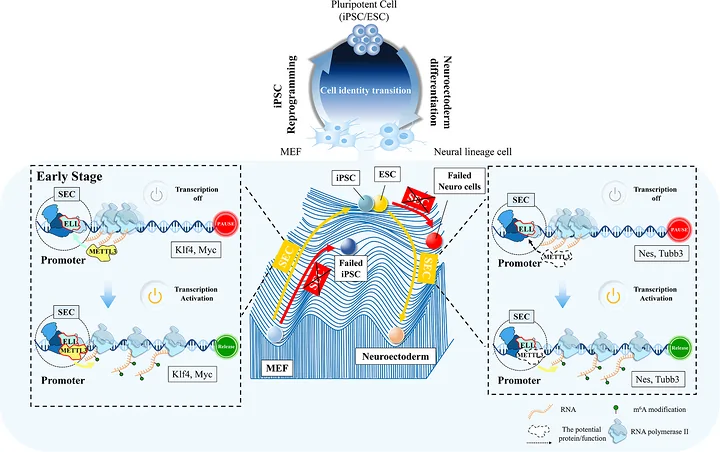

**Abbreviations**
4sU: (4‐Thiouridine)
AFF1/4: AF4/FMR2 Family Member 1/4
AP: Alkaline Phosphatase
ATCC: American Type Culture Collection
Co‐IP: Co‐immunoprecipitation
CUT&RUN: Cleavage Under Targets and Release Using Nuclease
CUT&Tag: Cleavage Under Targets and Tagmentation
DMEM: Dulbecco's Modified Eagle Medium
DOX: Doxycycline
EAF: ELL‐Associated Factors
EBs: Embryoid Bodies
ECDF: Empirical Cumulative Distribution Function
ELL: Eleven‐Nineteen Lysine‐Rich Leukemia
ENL: Eleven‐Nineteen Leukemia
ESC: Embryonic Stem Cell
EU: Ethynyl Uridine
FBS: Fetal Bovine Serum
GC: Gastric Cancer
GO: (Gene Ontology)
HLCs: (hepatocyte‐like cells)
IF: Immunofluorescence
iPSC: Induced Pluripotent Stem Cell
KO‐DMEM: KnockOut DMEM
KOSR: KnockOut Serum Replacement
m^6^A: N6‐Methyladenosine
MEFs: Mouse embryonic Fibroblasts
MNase: Micrococcal Nuclease
MTC: Methyltransferase Complex
NEAA: Nonessential Amino Acids
OE: Overexpression
PFA: Paraformaldehyde
PI: Propidium Iodide
RNA Pol II: RNA Polymerase II
pSer2: Ser2 Phosphorylation
PVDF: Polyvinylidene Fluoride
RA: Retinoic Acid
SD: Standard Deviations
SELECT: Single‐Base Elongation‐ and Ligation‐Based qPCR Amplification Method
SDS‐PAGE: SDS‐Polyacrylamide Gel Electrophoresis
SEC: Super Elongation Complex
TFs: Transcription Factors
TM: Traveling Matrix
TR: Traveling Ratio
TSS: Transcription Start Site

## 1 Background

Cell identity transition, also known as cell fate transition, is a central topic in biology. In eukaryotic cells, two opposing directions of cell identity transitions — from somatic cells to pluripotency and from pluripotent stem cells to lineage commitment — represent two major processes throughout life, such as organism development, immune reaction, tumorigenesis, and so on [1, 2]. Induced pluripotent stem cell (iPSC) reprogramming and embryonic stem cell (ESC) differentiation are two typical biological models that represent these processes, providing valuable systems for studying cell fate transitions in detail. During cell identity transitions, the gene transcriptional network is extensively remodeled. Among these changes, the activation of genes specific to the next cell identity is a core subject [3]. Over the past decades, much effort has been devoted to understanding the regulation of these key genes activation from various perspectives, including transcription factors (TFs), epigenetic modifications, metabolism alteration, and so on [4, 5, 6, 7, 8]. However, less attention has been paid to the role of intrinsic transcriptional programs of these key genes in regulating their activation during cell identity transitions. Gene transcription is a highly dynamic and heterogeneous process that primarily involves three finely regulated steps: initiation, elongation, and termination. Following transcription initiation and promoter clearance, RNA polymerase II (RNA Pol II) progresses downstream and pauses approximately 20–100 nucleotides from the transcription start site (TSS), awaiting a release signal [7]. Successfully released RNA Pol II carries out a series of downstream transcriptional events. In contrast, RNA Pol II that fails to be released from the pause site rapidly dissociates from the genome, leading to premature transcription termination [9]. Therefore, the molecules that facilitate the transition from transcription pause to release may serve as key drivers of cell identity transitions by activating next lineage‐specific genes.

Super Elongation Complex (SEC) was first identified in 2011 and is considered as one of the most pivotal regulators of transcription pause release to date [10]. SEC is composed of the elongation factors eleven‐nineteen lysine‐rich leukemia (ELL) 1/2/3, AF4/FMR2 family member 1/4 (AFF1/4), ELL‐associated factors (EAF) 1/2, and the YEATS domain‐containing epigenetic reader ENL (eleven‐nineteen leukemia) [11]. By facilitating the release of paused RNA Pol II, SEC plays vital roles in the activation of key genes and is therefore indispensable in a wide range of biological processes, including tumorigenesis, organ development, and others [12, 13, 14]. Despite numerous studies investigating SEC in various biological events, a systematic exploration of how SEC regulates cell identity transition — particularly in iPSC reprogramming and ESC lineage differentiation — is still lacking. Moreover, the precise mechanisms by which SEC regulates cell identity transitions remain largely unknown.

N6‐methyladenosine (m^6^A), an abundant modification on most eukaryotic mRNAs, is deposited by the m^6^A methyltransferase complex (MTC), and therefore closely linked to transcriptional programs [15]. For instance, JinDong Hao et al. recently revealed that METTL3‐mediated m^6^A modification regulates transcription termination through cooperation with DDX21 in HEK‐293T cells [16]. Intriguingly, previous studies have demonstrated that, in *Drosophila*, METTL3‐mediated m^6^A is sufficient to promote transcriptional pause release [17]. However, it remains unclear whether this mechanism also exists in mammals. In mammals, various studies have highlighted the essential roles of m^6^A in regulating cell identity transitions. For instance, knockout of METTL3, METTL14, or METTL16 in embryos leads to early post‐implantation embryonic lethality [18, 19, 20, 21]. The m^6^A modification mediated by METTL3 promotes gastric cancer (GC) cell proliferation, migration, and invasion through upregulation of LncRNA THAP7‐AS1 [22, 23]. In addition, it is generally accepted that early depletion of METTL3 impairs reprogramming efficiency, though the precise role of m^6^A in iPSC reprogramming remains debated [20, 24]. Collectively, these findings suggest that RNA m^6^A might act as an important factor facilitating cell identity transitions. However, the detailed regulatory mechanism of m^6^A during ESC differentiation and iPSC reprogramming is largely ambiguous.

In this study, in order to investigate whether SEC regulates cell identity transitions in different biological contexts, we used two independent models: mouse embryonic fibroblasts (MEFs)‐to‐iPSC reprogramming and ESC‐to‐neuroectoderm differentiation. We demonstrated that SEC is essential for the transition from MEFs to iPSCs, particularly during the early stage of reprogramming. During the early stage of iPSC reprogramming, SEC promotes the expression of key reprogramming genes, including Klf4 and Myc, by facilitating the release of pre‐established paused RNA Pol II at their loci, thereby supporting cell‐cycle progression and activation of the pluripotency network. Moreover, beyond the canonical mechanism, the SEC can also rely on an additional pathway involving cooperation with METTL3 to deposit m^6^A modifications and promote transcription pause release at the Klf4 and Myc loci. Similarly, in the ESC differentiation system, we also observed that SEC promotes the release of paused RNA Pol II to activate neuronal genes, such as Nes and Tubb3, thereby specifically facilitating the transition from ESCs to neuroectodermal lineage cells.

## 2 Results

### 2.1 SEC is Required for Reprogramming From MEF to iPSCs

We utilize the iPSC reprogramming as a biological model to investigate the role of SEC in regulating cell identity transition. To inhibit SEC function, we used KL‐2, a structure‐guided peptidomimetic SEC disruptor that selectively interferes with the interaction between the SEC scaffold protein AFF4 and Cyclin T1 (CCNT1), a subunit of P‐TEFb, thereby disrupting the assembly of functional SEC complexes (Figure S1A) [14]. We first inhibited SEC in MEFs (15 µm for 12 h) to assess its impact on basal cell survival and identity under non‐reprogramming conditions. We found that SEC inhibition in MEFs neither impairs cell proliferation nor induces significant apoptosis (Figure 1A, Figure S1B). These findings indicate that SEC deficiency does not affect basal survival or identity in MEFs under steady‐state conditions.

**FIGURE 1:**
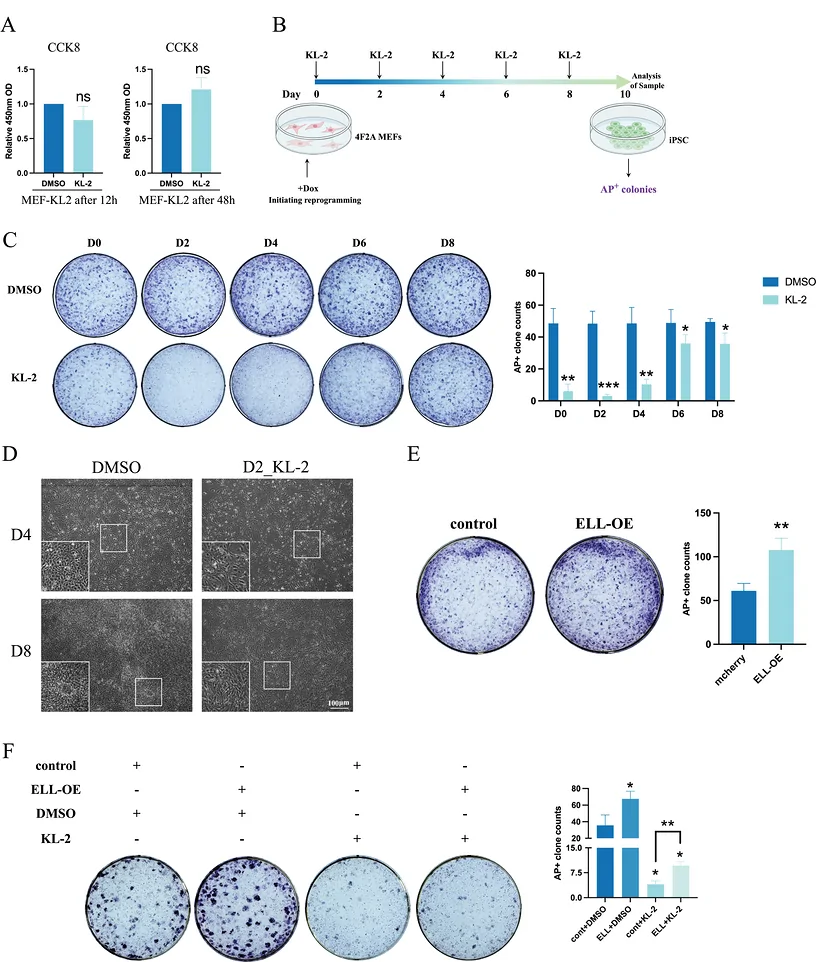
Early inhibition of SEC impairs iPSC reprogramming. A: CCK‐8 assay showing cell proliferation of MEF in DMSO and KL‐2 groups at 12 and 48 h post‐treatment. B: Schematic diagram illustrating the experimental design of KL‐2 treatment at specific time points during reprogramming. C: AP staining at day 10 (D10) showing iPSC colony formation under DMSO or KL‐2 treatment at different time points. The histogram quantifies the number of AP^+^ colonies. D: Phase‐contrast images displaying the cellular morphology at D4 and D8 of reprogramming under D2_KL‐2 treatment. Scale bar = 100 µm. E: AP staining at D10 showing iPSC colonies following ELL overexpression (ELL OE) at D0. The histogram shows the number of AP^+^ clone. F: AP staining at D10 showing the ELL OE partially alleviated the reduction in iPSC colony formation impaired by early KL‐2 treatment. The histogram displays the number of AP^+^ colonies in each group. Bars represent the mean ± standard deviation (SD) from n = 3 independent experiments. ns: not significant, ^*^ *p* < 0.05, ^**^ *p* < 0.01, ^***^ *p* < 0.001 by two‐tailed Student's t‐test.

We further initiated reprogramming of MEFs derived from 4F2A mice by adding doxycycline (DOX) to the culture medium, and inhibited SEC at specific time points using KL‐2. Reprogramming efficiency was evaluated on day 10 using alkaline phosphatase (AP) staining (Figure 1B). AP staining indicated that SEC inhibition at any time point markedly reduced iPSC reprogramming efficiency (Figure 1C). Notably, early SEC inhibition (D0, D2 and D4) resulted in the most pronounced impairment of reprogramming. Morphological observations further revealed that early SEC inhibition (D2_KL‐2 treatment; hereafter referred to as early SEC inhibition) caused reprogramming cells to remain in a mesenchymal state, failing to acquire epithelial morphology by day 4 and to form iPSC colonies by day 8 (Figure 1D). Similarly, using siRNA, early interference (D0 treatment) of SEC components AFF4 and ELL‐either individually or in combination‐also markedly reduced reprogramming efficiency (Figure S1C,D). Moreover, ELL interference resulted in a more significant decrease of reprogramming efficiency than AFF4. In order to achieve more stable suppression of ELL expression, we performed shRNA‐mediated ELL knockdown at D0 of iPSC reprogramming and found that it also markedly reduced reprogramming efficiency (Figure S1E–F). Since ELL is also the primary component directly responsible for transcription elongation within SEC [25, 26], we overexpressed (OE) ELL to enhance SEC activity (Figure S1G). Overexpression of ELL significantly promote MEF reprogramming into iPSCs (Figure 1E). Moreover, ELL overexpression partially alleviated the impaired reprogramming efficiency caused by early KL‐2 treatment (Figure 1F). Together, these findings demonstrate that SEC is a critical driver of iPSC reprogramming, with ELL potentially serving as one of the core components mediating this function.

### 2.2 SEC Mediates Early Transcriptional Activation of Key Genes During Reprogramming

According to our results, early inhibition of SEC has a stronger inhibitory effect on iPSC reprogramming than mid‐late inhibition (Figure 1C). This suggests that SEC plays a more critical role during the early stage; therefore, subsequent analyses primarily focused on its function during the early stage. To identify genes regulated by SEC during the early stage, we performed RNA‐seq on reprogramming cells at day 4 following early SEC inhibition (Figure 2A). This inhibition resulted in downregulation of more than 50% of differentially expressed genes, including numerous stem cell‐associated and cell cycle‐related genes, such as Klf4 and Myc (Figure 2A,B). It has been well established that both the activation of the pluripotency gene network and maintenance of the cell cycle are critical events in iPSC reprogramming and directly influence reprogramming efficiency [27, 28, 29, 30]. We therefore specifically analyzed the overall expression patterns of pluripotency‐ and cell cycle–related genes obtained from the Gene Ontology (GO) database and found that early inhibition of the SEC effectively decreased the transcription of most of these genes (Figure S1H). Among these genes, Klf4 and Myc are core reprogramming factors that play essential roles in multiple key events during reprogramming, including maintenance of the cell cycle and activation of the pluripotency gene network [27]. Moreover, previous studies have demonstrated that early transcriptional activation of Klf4 and Myc triggers the first transcriptional wave, thereby establishing them as decisive factors in iPSC reprogramming [27]. Accordingly, we designated Klf4 and Myc as representative core targets of the SEC in subsequent analyses. qPCR analysis further confirmed that early SEC inhibition or knockdown significantly reduced the expression of Klf4, Myc, and other key genes, including the cell cycle gene Orc1 and the pluripotency‐associated gene Sall1 (Figure 2C,D). These results suggest that early inhibition of SEC impairs the activation of various key reprogramming genes and might further impede both the maintenance of the cell cycle and the activation of the pluripotency gene network in reprogramming cells.

**FIGURE 2:**
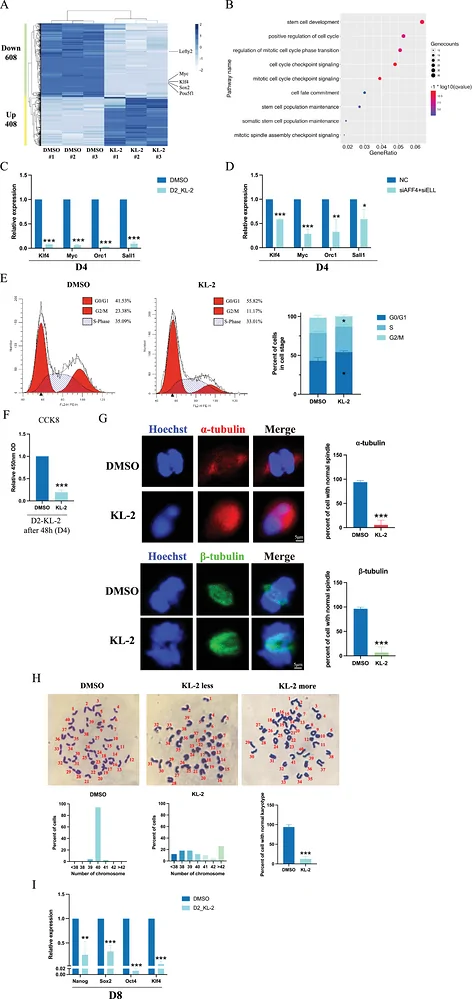
Early SEC inhibition disrupts core reprogramming gene expression and key reprogramming events. A: RNA‐seq analysis showing differential gene expression at D4 following KL‐2 treatment at D2 (*p* < 0.05 and |Log_2_FC| > 1.5). B: GO enrichment analysis of downregulated genes identified in Figure 2A. C‐D: qPCR validation of SEC target genes (Klf4, Myc, Orc1, and Sall1) expression at D4 after KL‐2 treatment at D2 (C) or siAFF4 + siELL co‐knockdown at D0 (D). E: Cell cycle distribution of reprogramming cells at D4 under DMSO and D2_KL‐2 treatment, analyzed by propidium iodide (PI) staining and flow cytometry. The stacked bar plot shows the percentage of cells in each phase. F: CCK‐8 assay showing reprogramming cell proliferation at D4 (48 h post‐D2_KL‐2 treatment). G: Immunofluorescence staining showing spindle morphology in reprogramming cells (D4) under D2_KL‐2 treatment using α‐tubulin (top) and β‐tubulin (bottom) markers. Scale bar = 5 µm. The histogram quantifies the proportion of cells with normal spindle structures. H: Karyotype analysis showing chromosome number distribution in D4 reprogramming cells treated with D2_KL‐2 (top). The histogram (bottom left) shows the percentage of cells with various chromosome numbers in DMSO and D2_KL‐2 groups. The histogram (bottom right) shows the proportion of cells with a normal karyotype. I: qPCR analysis of pluripotency gene expression (Nanog, Sox2, Oct4, and Klf4) at D8 under D2_KL‐2 treatment. Bars represent the mean ± SD from n = 3 independent experiments. ^*^ *p* < 0.05, ^**^ *p* < 0.01, ^***^ *p* < 0.001 by two‐tailed Student's t‐test.

To validate this hypothesis, cell cycle analysis was performed and revealed that early SEC inhibition induced pronounced G0/G1 arrest, characterized by a significant increase in the G0/G1 cells, a mild reduction in the S‐phase cells, and a marked decrease in the G2/M‐phase cells (Figure 2E). CCK‐8 assays further confirmed that cell proliferation was significantly decreased following early KL‐2 treatment (Figure 2F). Intriguingly, in MEFs with a stable somatic cell identity, SEC inhibition by KL‐2 did not affect cell proliferation (Figure 1A), suggesting that the proliferation defect observed during reprogramming is more likely a downstream consequence of impaired SEC‐dependent reprogramming programs rather than a primary direct cytotoxic effect of KL‐2. In addition, the spindle is a key organelle responsible for the equal segregation of chromosomes into daughter cells [31]. Hence, we examined spindle morphology and found that early inhibition of the SEC disrupted spindle assembly and led to abnormal spindle structures, such as multipolar spindles (Figure 2G). Furthermore, karyotype analysis revealed that most reprogramming cells with early SEC inhibition exhibited an abnormal chromosome number, with both increases and decreases observed (Figure 2H). These results indicate that, during iPSC reprogramming, early SEC inhibition downregulates the expression of these key reprogramming genes, thereby compromising spindle structure and function, leading to abnormal karyotypes in daughter cells, and ultimately impairing cell‐cycle progression and proliferation in reprogramming cells. Finally, qPCR analysis showed that early SEC inhibition leads to failure in the activation of several pluripotency genes at mid‐late stage, confirming the failure of activation of the pluripotency gene network (Figure 2I). In conclusion, these results demonstrate that early inhibition of SEC impedes the early transcriptional activation of various key reprogramming genes, leading to cell cycle arrest, failure in pluripotency network activation, and ultimately impairing the transition from MEF to iPSC.

### 2.3 Genome‐Wide Dynamics of Transcription Pause Release During iPSC Reprogramming

Given that the SEC is a key regulator of transcription pause release, we hypothesized that SEC activate Klf4 and Myc, the representative key reprogramming genes, may be through facilitation of RNA Pol II pause release at these loci during the early stage of iPSC reprogramming. First, we utilized the RNA Pol II ChIP‐seq data (GSE67520) in different reprogramming days (eg., D0, D5, D11, and iPSCs) to investigate the dynamics of the RNA Pol II pause and release during reprogramming [32]. To quantitatively assess RNA Pol II pause release, we applied two analytical methods to RNA Pol II ChIP‐seq and nascent RNA sequencing signals: (a) the Traveling Ratio (TR, also known as the Pause Index) and (b) the 2D Traveling Matrix (TM) (Figure 3A). Following established standards from previous studies, we defined the promoter‐proximal region as 30 bp upstream to 300 bp downstream of the TSS, and the gene body as the region from 300 to 3000 bp downstream of the TSS [33]. The TR is calculated as the ratio of RNA Pol II signal in the promoter‐proximal region to that in the gene body. An increase in transcriptional pause leads to a higher TR value, characterized by elevated RNA Pol II signal in the promoter‐proximal region and reduced signal in the gene body. However, because the TR is a one‐dimensional metric, it cannot distinguish whether a given TR value results from increased promoter signal, decreased gene body signal, or both. This limitation is addressed by the TM method, proposed by Beckedorff et al. [34]. In the TM method, changes in RNA Pol II signal density at the promoter‐proximal region and gene body are simultaneously mapped to the *X*‐ and *Y*‐axes, respectively, forming a two‐dimensional topographic. Color intensity reflects the density of genes located at specific coordinate positions. In TM analysis, the coordinate plane is divided into four quadrants, each representing a distinct gene cluster. Cluster I, characterized by reduced Pol II signal in the promoter‐proximal region and increased signal in the gene body, represents genes with enhanced transcription release. Conversely, Cluster IV shows increased Pol II signal in the promoter‐proximal region and reduced signal in the gene body, indicating enhanced transcription pause.

**FIGURE 3:**
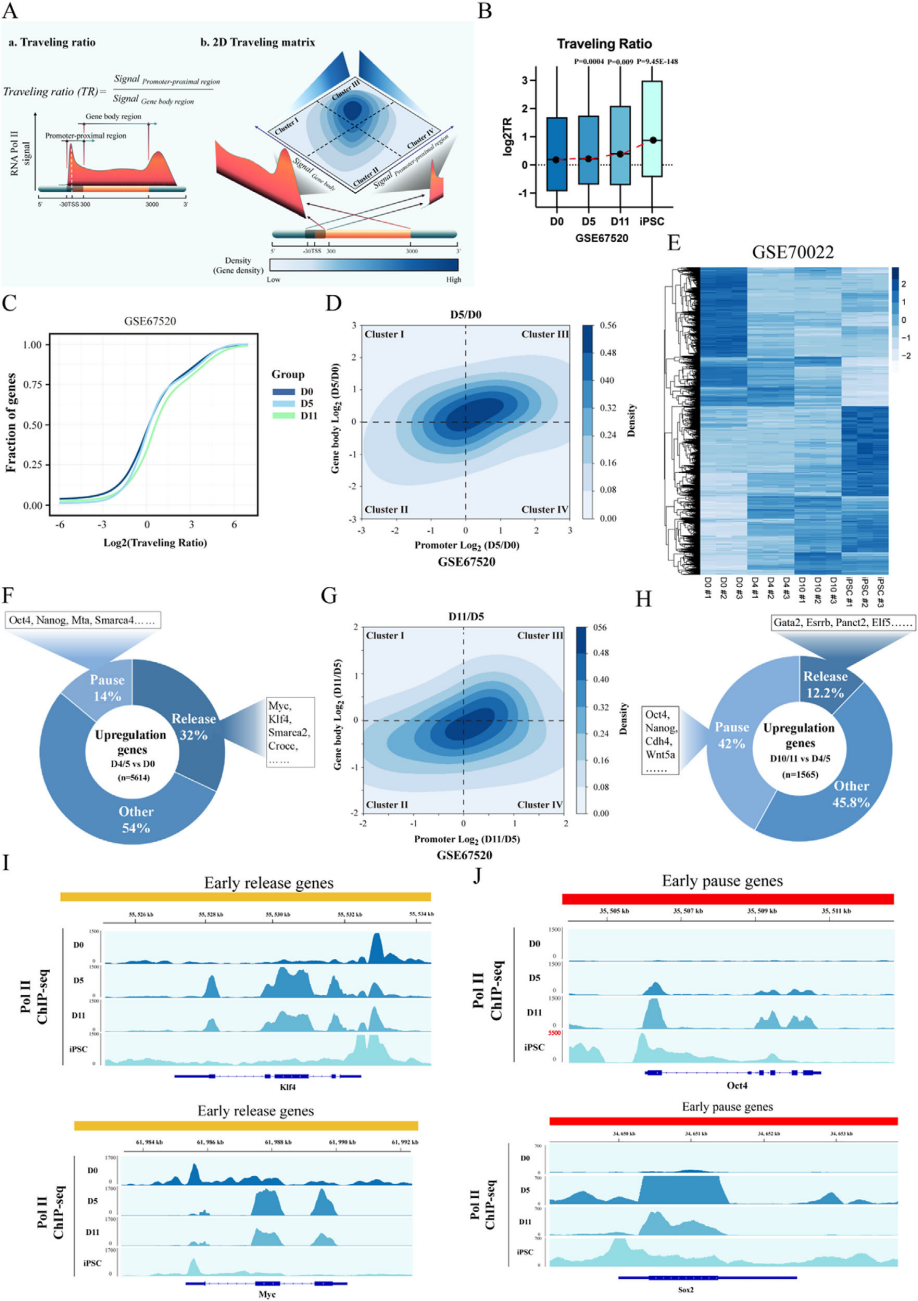
TR and TM analysis for the transcription pause and release during iPSC reprogramming. (A) Schematic diagram illustrating the theory of TR and TM analysis. (B) Box plot showing the log_2_TR values at the indicated time points during reprogramming, based on published RNA Pol II ChIP‐seq data (GSE67520). *p*‐values were calculated using a t‐test and are shown above each box. (C) Empirical cumulative distribution function (ECDF) plot of log_2_TR at day 0 (D0), day 5 (D5), and day 11 (D11) of reprogramming. (D) 2D topographic map of TM analysis illustrating differential enrichment of RNA Pol II at promoter and gene body regions in D5 versus D0. Cluster I represents genes with increased transcription release, while Cluster IV represents genes with enhanced transcription pause. (E) Heatmap displaying differential gene expression among D0, D4, D10, and iPSC, based on published RNA‐seq data (GSE70022) (*p* < 0.05 and |Log_2_FC| > 1.5). (F) Pie chart showing the proportions of pause‐increased genes, release‐increased genes, and other categories among upregulated genes in D4/5 compared to D0 (n = 5614). (G) 2D topographic map of TM analysis showing differential RNA Pol II enrichment at promoter and gene body regions in D11 versus D5. (H) Pie chart showing the proportions of pause‐increased genes, release‐increased genes, and other categories among upregulated genes in D10/11 compared to D4/5 (n = 1565). (I, J) ChIP‐seq browser tracks showing RNA Pol II enrichment at representative early released genes (e.g., Klf4 and Myc) and early or mid‐late paused genes (e.g., Oct4 and Sox2) at D0, D5, D11, and iPSC stages.

We performed TR analysis for ChIP‐seq data and observed a gradual increase in transcription pause throughout the reprogramming process (Figure 3B,C). Based on the reprogramming stage criteria previously proposed by our group [35], only a mild increase in TR was observed during the early stage of reprogramming (D0‐D5), whereas a marked elevation occurred during the mid‐late stage (D5‐D11). To further resolve transcriptional dynamics in the early stage, we applied the TM analysis. Consistent with the TR results, a few genes exhibited increased transcription pause (Cluster IV), while numerous of genes showed rapid release of paused RNA Pol II (Cluster I) (Figure 3D, Figure S2A). We reanalyzed previous RNA‐seq data (GSE70022) with similar time periods (eg., D0, D4, D10, iPSCs) to RNA Pol II ChIP‐seq, thereby identifying genes activated in the early and mid‐late stages of reprogramming, respectively (Figure 3E) [36]. After calculating the proportions of genes with increased transcription pause or release among early activated genes (D0‐D4/5), we found that approximately 14% showed increased pause, while 32% exhibited increased release (Figure 3F, Figure S2B). In contrast, TM analysis of the mid‐late stage revealed that a large number of genes showed significantly enhanced transcription pause, whereas only a small part exhibited increased transcriptional release (Figure 3G, Figure S2C). Moreover, genes with increased pause accounted for 42% of mid‐late stage activated genes (D4/5‐D10/11), while those with increased release accounted for only 12.2% (Figure 3H, Figure S2D).

Further analysis found that core reprogramming genes, such as Klf4 and Myc, had pre‐established paused RNA Pol II at the promoter‐proximal region prior to the initiation of reprogramming (Figure 3I, Figure S2E). Upon reprogramming initiation, these genes rapidly release the paused RNA Pol II, thereby efficiently upregulating transcription. In contrast, other critical reprogramming genes, such as Oct4 and Sox2, do not exhibit pre‐established paused RNA Pol II (Figure 3J). Instead, they gradually acquire transcription pause after reprogramming initiation and ultimately activate transcription through a coupled pause and release mechanism. GO analysis of genes showing increased transcription pause or release at different stages of reprogramming revealed that all groups were strongly associated with multiple key reprogramming events (Figure S2F,G). Moreover, to validate the above ChIP‐seq analysis, a published GRO‐seq dataset (GSE27037) was analyzed to assess direct transcriptional patterns [37]. MEFs were hypothetically regarded as the reprogramming origin, whereas ESCs were assumed to represent the putative reprogramming endpoint. Similar to the ChIP‐seq results, an increased TR value was also observed in ESCs compared with MEFs (Figure S2H). TM analysis further revealed that numerous genes exhibited either rapid transcription release or increased transcription pause in ESCs compared with MEFs (Figure S2I,J). We next quantified the proportions of paused and released genes among upregulated genes in ESCs vs. MEFs from RNA‐seq data (GSE113430), and found that paused and released genes accounted for 23% and 36%, respectively (Figure S2K,L) [38]. Specifically, the released Myc gene was also observed when comparing ESCs with MEFs (Figure S2M). Additionally, a similar analysis was performed using GRO‐seq (GSE117086) and RNA‐seq (GSE138312) datasets, in which hepatocyte‐like cells (HLCs) were considered the putative reprogramming origin, whereas human iPSCs were regarded as the putative reprogramming destination [39, 40]. A similar pattern of transcription release and transcription pause was also observed during this putative reprogramming process (Figure S2N–Q).

In conclusion, our results uncover the dynamics of transcription pause and release during iPSC reprogramming and demonstrate that the early‐activated genes Klf4 and Myc are primarily upregulated through the rapid release of pre‐established paused RNA Pol II. Previous studies have proposed that, at later stages, the release of paused RNA Pol II at pluripotency genes constitutes a rate‐limiting step in reprogramming [41]. Based on our findings, we propose that, during the early stage of iPSC reprogramming, the efficient release of pre‐established paused RNA Pol II at core reprogramming genes, such as Klf4 and Myc, may constitute an additional rate‐limiting step in the reprogramming process.

### 2.4 SEC Facilitates Activation of Klf4 and Myc via the Release of Paused RNA Pol II

In order to determine whether SEC regulates the transcription pause release during iPSC reprogramming, we performed Cleavage Under Targets and Tagmentation (CUT&Tag) for RNA Pol II in reprogramming cells (D4), followed by TM analysis to assess the effect of early KL‐2 treatment on transcription pause release of SEC target genes (Figure 4A). TM analysis revealed that early inhibition of SEC significantly increased transcription pauses across numerous target genes (Figure 4A, Figure S3A). TR analysis confirmed the enhanced pause in Cluster IV genes identified in the TM analysis (Figure 4B). In Cluster IV genes, RNA Pol II accumulated at promoter‐proximal regions with little occupancy along the gene bodies, indicating the enhanced transcriptional pause (Figure 4C). Consistent with the CUT&Tag results, 5‐ethynyl uridine (EU) incorporation assays performed at day 4 (D4) also revealed a significant reduction in nascent RNA synthesis following early KL‐2 treatment or ELL interference (Figure S3B,C). Moreover, overexpression of ELL significantly enhanced nascent RNA synthesis (Figure S3D). These results indicate that the SEC regulates global transcription during the early stage of iPSC reprogramming by facilitating transcription pause release.

**FIGURE 4:**
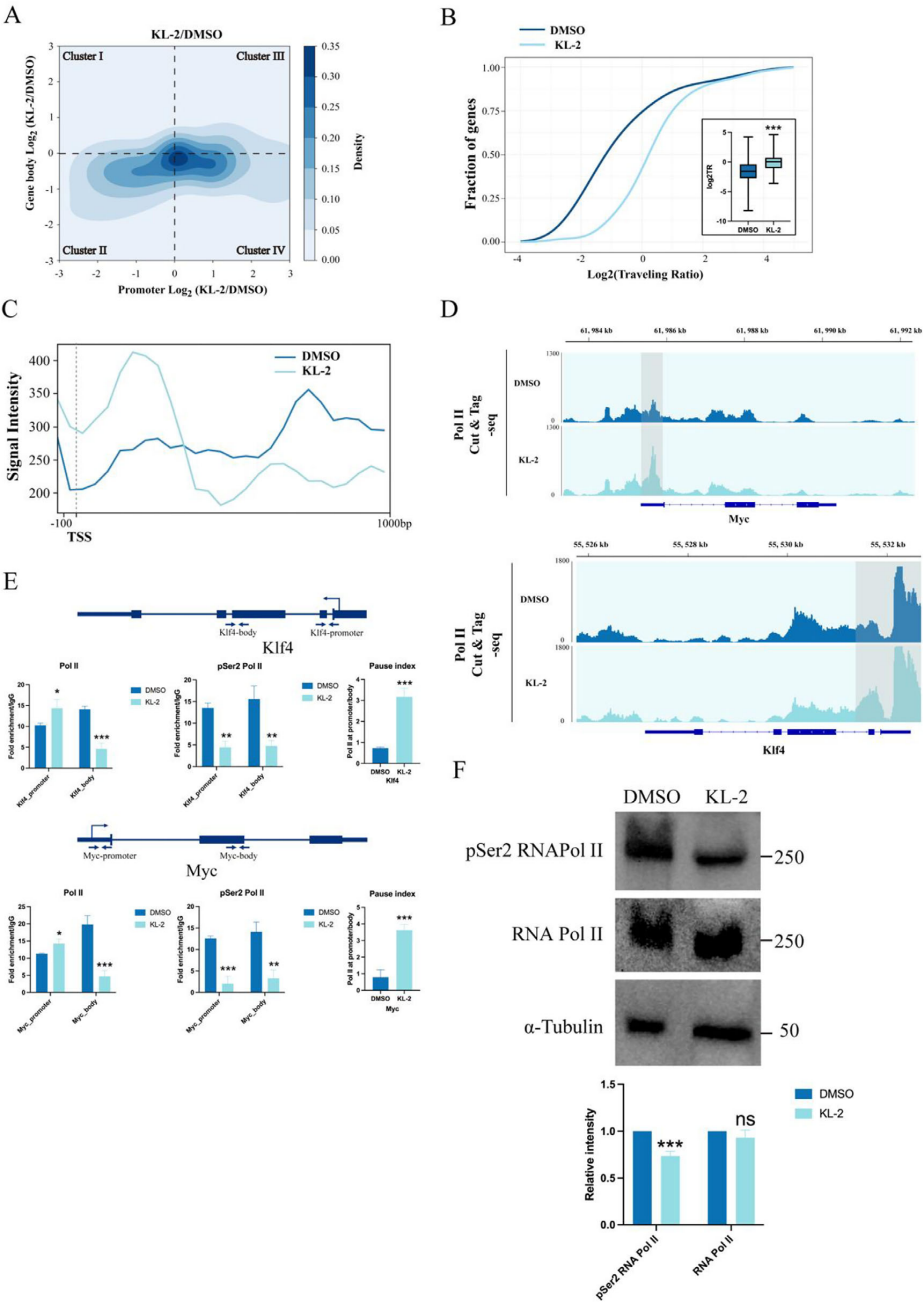
SEC triggers rapid transcription pause release at Klf4 and Myc in the early stage. (A) 2D topographic map from TM analysis showing differential enrichment of RNA Pol II (D4) at promoter and gene body regions between D2_KL‐2 and DMSO conditions. (B) ECDF plot and box plot displaying log_2_‐transformed TR values for Cluster IV genes (from Figure 4A) in D2_KL‐2 versus DMSO. (C) Comparison of RNA Pol II density at Cluster IV genes in reprogramming cells (D4) under D2_KL‐2 treatment. (D) CUT&Tag browser tracks showing RNA Pol II occupancy at Klf4 and Myc loci. (E) ChIP‐qPCR analysis showing RNA Pol II and pSer2 RNA Pol II enrichment at promoter and gene body regions of Klf4 and Myc in D4 reprogramming cells treated with D2_KL‐2 (left). Right: Pause index of RNA Pol II at Klf4 and Myc. (F) Western blot analysis showing the total protein levels of RNA Pol II and pSer2 RNA Pol II in D4 reprogramming cells upon D2_KL‐2 treatment. The original blots are provided in Files S3. The bar plot shows statistical results of gray value. Bars represent the mean ± SD from n = 3 independent experiments. ns: not significant, ^*^ *p* < 0.05, ^**^ *p* < 0.01, ^***^ *p* < 0.001 by two‐tailed Student's t‐test.

Genome browser tracks and ChIP‐qPCR assays both showed that early SEC inhibition led to increased RNA Pol II occupancy at the promoters of two representative target genes Klf4 and Myc, along with reduced occupancy in their gene bodies (Figure 4D,E). Moreover, ChIP‐qPCR assays also revealed that RNA Pol II Ser2 phosphorylation (pSer2), a marker of transcriptional elongation, was significantly reduced at both the promoter and gene body regions of Klf4 and Myc upon early inhibition of SEC, confirming defective transcription pause release (Figure 4E). Pause index analysis further confirmed that early SEC inhibition significantly enhanced transcription pauses on Klf4 and Myc (Figure 4E). It also found that total pSer2 RNA Pol II levels were decreased, whereas total RNA Pol II levels remained unchanged following early KL‐2 treatment, supporting the occurrence of transcriptional release failure (Figure 4F). Collectively, these data demonstrate that during the early stage of reprogramming, the SEC activates Klf4 and Myc expression by facilitating the rapid release of pre‐established paused RNA Pol II.

### 2.5 SEC Regulates m^6^A Deposition on Klf4 and Myc During Early Stage of Reprogramming

We then aimed to elucidate the mechanism by which the SEC regulates RNA Pol II pause release at the Klf4 and Myc loci during the early stage of reprogramming. Previous studies have shown that SEC promotes transcription pause release through its P‐TEFb kinase module, which phosphorylates the RNA Pol II C‐terminal domain (CTD) and the pause factors DSIF and NELF [42]. Consistent with this canonical mechanism, early inhibition of the SEC indeed decreased pSer2 RNA Pol II levels without altering total RNA Pol II levels (Figure 4F). Moreover, the phosphorylation of NELF‐e was also reduced following the early SEC inhibition (Figure S4A). However, in addition to this canonical mechanism, we wondered whether SEC may engage additional mechanisms to facilitate the transcription pause release at the Klf4 and Myc loci during the early stage of reprogramming. Recent studies have demonstrated that RNA m^6^A modification, which is closely associated with transcriptional programs, is capable of promoting transcription pause release in *Drosophila* [15, 17]. Although this mechanism has not yet been validated in mammals, we hypothesized that the SEC may cooperate with m^6^A modification to regulate transcription pause release. By analyzing published ChIP‐seq datasets of the SEC components AFF4 (GSE30267) and ELL (GSE32120), together with MeRIP‐seq data (GSE202846), we found that more than 40% of protein‐coding transcripts associated with SEC target genes contain m^6^A modifications (Figure S4B) [43, 44, 45]. Hence, this finding further suggests a potential link between m^6^A modification and the transcription pause‐release factor SEC. To validate this hypothesis, using RNA dot blot and ELISA assays, we measured the global m^6^A modification levels of total RNA in reprogramming cells following early SEC inhibition. As predicted, early inhibition of the SEC led to a significant reduction in global m^6^A levels when normalized to equal amounts of RNA (Figure 5A,B). Subsequently, we performed MeRIP‐seq to identify m^6^A‐modified mRNA regulated by SEC following early KL‐2 treatment. Early inhibition of the SEC modestly altered the m^6^A distribution pattern, slightly reducing enrichment at the 3′ UTR and stop codon while increasing enrichment within the CDS and 5′ UTR (Figure S4C). Differential analysis was performed to identify hypo‐ and hyper‐methylated m^6^A peaks following early SEC inhibition. Consistent with the RNA dot blot and ELISA results, early SEC inhibition led to significantly decreased m^6^A enrichment in 6006 transcripts, whereas only 749 transcripts showed significantly increased enrichment (Figure 5C,D). Motif analysis revealed that the differential m^6^A peaks contain the classic DRACH consensus sequence (D = A/G/U, R = A/G, H = A/C/U), confirming their identity as bona fide m^6^A sites (Figure S4D). These hypo‐methylated m^6^A peaks were primarily enriched in the CDS and 3' UTR regions (Figure 5E). By comparing RNA‐seq and MeRIP‐seq data, the genes that exhibited both transcription downregulation and reduced m^6^A levels after early SEC inhibition were identified (Figure 5F). GO analysis revealed that most of these overlapping genes are also related to pluripotency and cell cycle regulation (Figure 5G). Notably, the two representative SEC‐target genes Klf4 and Myc were again identified among these genes (Figure 5H).

**FIGURE 5:**
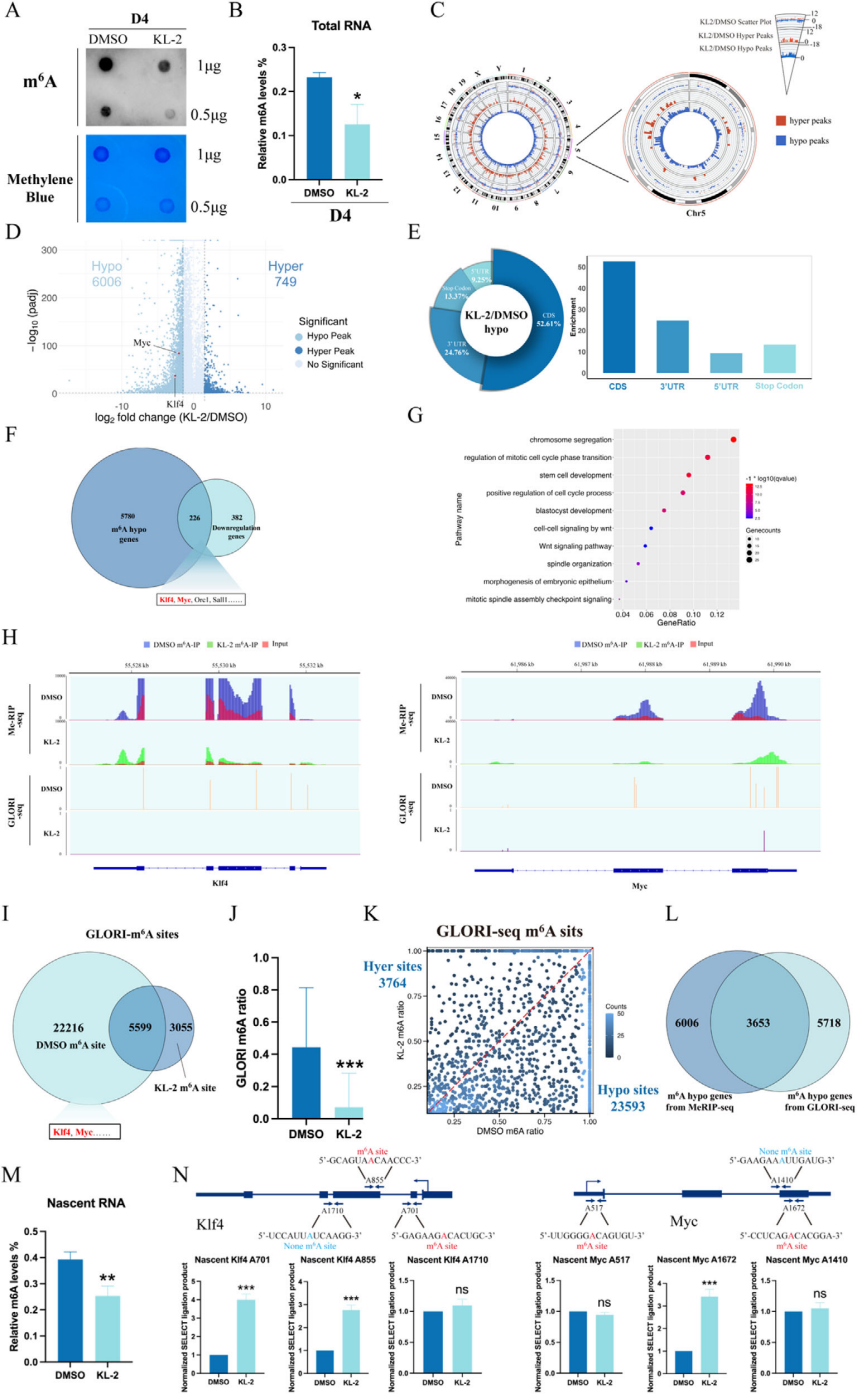
SEC regulates m^6^A modification during the early stage of reprogramming. (A) RNA dot blot showing global m^6^A levels of total RNA at D4 following KL‐2 treatment at D2. Methylene blue staining was used as a loading control. The original blot is provided in Files S3. (B) ELISA assay quantification of global m^6^A levels of total RNA at D4 after D2 KL‐2 treatment. (C) Circos plots showing genome‐wide (left) and chromosome 5 (Chr5, right) distribution of hypermethylated and hypomethylated m^6^A peaks at D4 after D2_KL‐2 treatment. (D) Volcano plot displaying genes with differential m^6^A peaks between D2_KL‐2 and DMSO groups (Padj < 0.05, |log_2_FC| > 1.5). (E) Distribution of hypomethylated m^6^A peaks in D4 reprogramming cells upon D2_KL‐2 treatment based on MeRIP‐seq data. Pie chart (left) indicates the percentage of peaks located in CDS, 3′UTR, 5′UTR, and stop codon regions. Bar plot (right) quantifies enrichment across these regions. (F) Venn diagram showing the overlap between transcripts with decreased m^6^A level and decreased expression upon D2_KL‐2 treatment. (G) GO enrichment analysis of genes with concurrent reductions in m^6^A modification and expression (from Figure 2A) following KL‐2 treatment at D2. (H) MeRIP‐seq (Top) and GLORI‐seq (Bottom) browser tracks illustrating m^6^A ratio differences in Klf4 and Myc transcripts between DMSO and D2_KL‐2 groups. (I) Overlap analysis of m^6^A sites identified by GLORI‐seq in the DMSO‐ and KL‐2‐treated groups. (J) Histogram showing global m^6^A levels measured by GLORI‐seq following early SEC inhibition. (K) Scatterplot comparing site‐specific m^6^A ratios between the DMSO‐ and KL‐2‐treated groups, as determined by GLORI‐seq. (L) Venn diagram showing the overlap between genes exhibiting reduced m^6^A modification after early KL‐2 treatment, as identified by GLORI‐seq and MeRIP‐seq. (M) ELISA assay quantification of global m^6^A levels of nascent RNA at D4 after D2 KL‐2 treatment. (N) Normalized levels of SELECT ligation products at selected sites in nascent Klf4 transcripts (A701, A855, and A1710; left) and nascent Myc transcripts (A517, A1410, and A1672; right). The SELECT product signal at each target site was normalized to the abundance of the corresponding transcript containing that site. Bars represent the mean ± SD from n = 3 independent experiments. ns: not significant, ^*^ *p* < 0.05, ^**^ *p* < 0.01, ^***^ *p* < 0.001 by two‐tailed Student's t‐test.

To further validate SEC‐mediated regulation of m^6^A modification, we employed GLORI‐seq, an antibody‐independent m^6^A profiling method, to map and quantify m^6^A sites at single‐nucleotide resolution following SEC inhibition during the early stage of iPSC reprogramming. In GLORI‐seq, unmodified adenosines (A) are converted to inosines (I) through glyoxal‐ and nitrite‐mediated deamination. During reverse transcription, inosines are read as guanosines (G), whereas m^6^A residues remain resistant to deamination and are still read as adenosines (A). Consequently, the m^6^A modification level at each site can be quantitatively estimated from the proportion of A reads among the total A and G reads obtained by high‐throughput sequencing [46]. By GLORI‐seq, a total of 27815 and 8654 m^6^A sites were identified in the DMSO‐ and KL‐2‐treated groups, respectively (Figure 5I). The conserved DRACH motif was also enriched among the m^6^A sites identified in both the DMSO‐ and KL‐2‐treated groups (Figure S4E). GLORI‐seq further revealed a modest decrease in the relative enrichment of m^6^A sites within the 3′ UTR, accompanied by increased enrichment within the coding sequence (CDS), stop condon and 5′ UTR (Figure S4F). Moreover, GLORI‐seq also indicated that SEC inhibition during early iPSC reprogramming markedly reduced global m^6^A levels in reprogramming cells (Figure 5J). Specifically, 23593 m^6^A sites exhibited decreased modification levels, whereas only 3764 sites exhibited increased modification levels following early KL‐2 treatment (Figure 5K). Comparison of genes exhibiting reduced m^6^A modification showed that more than 60% were commonly identified by both MeRIP‐seq and GLORI‐seq (Figure 5L). Furthermore, GLORI‐seq also revealed that m^6^A modification levels on the Klf4 and Myc transcripts were markedly reduced following SEC inhibition during the early stage of iPSC reprogramming (Figure 5H, Figure S4G).

Given that SEC promotes transcription pause release and thereby regulates nascent RNA synthesis (Figures S3B–D and S4H), we asked whether SEC regulates m^6^A deposition on nascent RNA. To address this issue, D4 reprogramming cells were metabolically labeled with 4‐thiouridine (4sU) for 1 h. The 4sU‐labeled RNA was biotinylated, affinity‐purified, and subjected to m^6^A analysis. RNA m^6^A ELISA was performed to assess global m^6^A levels in the nascent RNA and showed that early SEC inhibition significantly decreased m^6^A levels in nascent RNA (Figure 5M). Furthermore, in order to assess site‐specific m^6^A changes in nascent Klf4 and Myc transcripts, we used the established single‐base elongation‐ and ligation‐based qPCR amplification method (SELECT) [47, 48, 49]. SELECT exploits the ability of m^6^A to impede single‐base elongation by DNA polymerase and subsequent nick ligation by DNA ligase. The resulting ligation products were quantified by qPCR, with lower product abundance indicating higher m^6^A levels at the interrogated site because m^6^A impedes ligation between the upstream and downstream probes. SELECT analysis showed that early SEC inhibition significantly decreased m^6^A levels at both the promoter‐proximal A701 site and the A855 site in the gene body region of the nascent Klf4 transcript, whereas no significant change was detected at the non‐m^6^A negative control site A1710 (Figure 5N). Moreover, early SEC inhibition significantly reduced m^6^A levels at A1672 in the 3′ region of the nascent Myc transcript, whereas no significant change was detected at the non‐m^6^A negative control site A1410 (Figure 5N). At the promoter‐proximal A517 site of nascent Myc transcripts, however, m^6^A levels showed a slight but statistically nonsignificant increase, consistent with the MeRIP‐seq and GLORI‐seq profile (Figure 5N). Collectively, these results support a role for SEC in the co‐transcriptional regulation of m^6^A deposition on nascent RNA, with site‐specific effects observed in the representative SEC target transcripts Klf4 and Myc.

### 2.6 SEC Recruits METTL3 to the Klf4 and Myc loci in Reprogramming Cells

As m^6^A modification is primarily mediated by the MTC, mainly composed of METTL3, METTL14, and WTAP [15], we hence postulate a potential interaction between SEC and MTC. To test this hypothesis, we performed molecular docking to predict and compare the interaction potential between SEC components and MTC members, using previously published protein structure data. By comparing the average hydrogen bond distances between individual SEC components and MTC members, we identified ELL‐METTL3 and AFF4‐METTL3 as the top 2 likely interaction models (Figure 6A, Figure S5A). We further reanalyzed published ChIP‐seq data for AFF4 (GSE30267), ELL (GSE32120), and METTL3 (GSE154135) to investigate their genomic co‐localization [43, 44, 50]. Heatmap and genomic distribution analyses revealed that both AFF4 and ELL are highly co‐localized with METTL3 near TSSs, and that more than 50% of SEC‐binding genes co‐occupied by AFF4 and ELL are also bound by METTL3 (Figure S5B–D). However, a higher similarity was observed between ELL and METTL3 (Figure S5B,D). Moreover, correlation analysis revealed that the genomic binding profiles of both AFF4 and ELL, but not of the unrelated negative control sample H3K18la, were positively correlated with that of METTL3, with ELL showing a stronger correlation (Figure S5E). Specifically, co‐localization of AFF4, ELL, METTL3, paused RNA Pol II was observed at the Klf4 locus (Figure S5F). These findings suggest that the SEC component ELL has the greatest potential to interact with METTL3.

**FIGURE 6:**
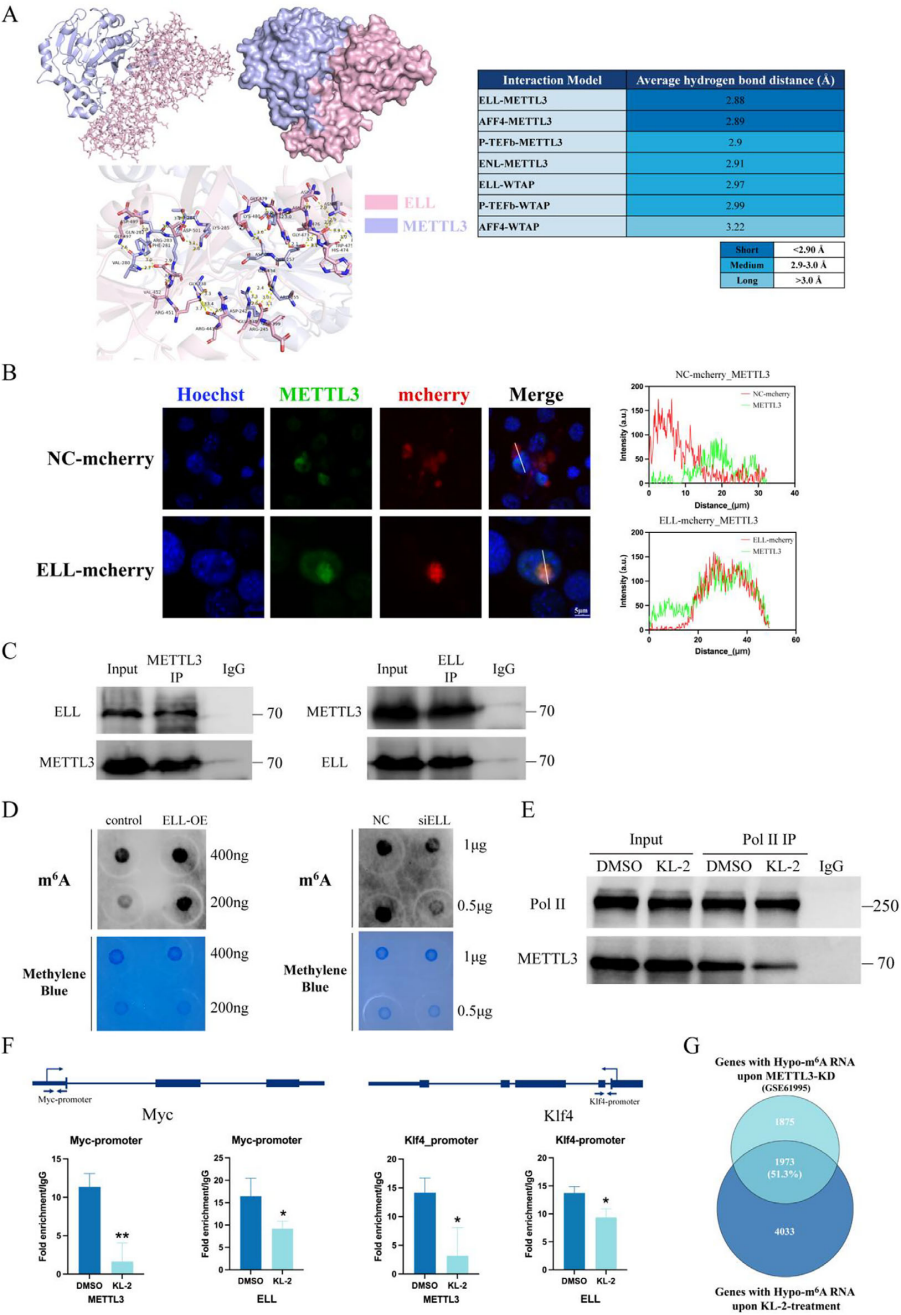
SEC facilitates METTL3 recruitment to the Klf4 and Myc loci during reprogramming. (A) Molecular docking showing the interaction mode between ELL and METTL3 (top). Hydrogen bond distances are labeled in the image (below). The table summarizes the average hydrogen bond distances across models (right). (B) Immunofluorescence images showing co‐localization of ELL‐mCherry and METTL3 at D4. Line plots on the right show pixel intensity profiles along the indicated lines in the merged images. Scale bar = 5 µm. (C) Co‐IP showing the interaction between METTL3 and ELL. The original blots are provided in Files S3. (D) RNA dot blot showing global m^6^A levels of total RNA at D4 following ELL overexpression (OE) or knockdown (siELL). Methylene blue was used as a loading control. The original blots are provided in Files S3. (E) Co‐IP showing the effect of D2_KL‐2 treatment on the interaction between METTL3 and RNA Pol II in reprogramming cells at D4. The original blots are provided in Files S3. (F) ChIP‐qPCR analysis showing the binding of METTL3 and ELL at the promoters of Myc and Klf4 at D4 under D2_KL‐2 treatment. (G) Venn diagram showing the overlap between transcripts with decreased m^6^A levels upon D2_KL‐2 treatment (this study) and those from published METTL3 knockdown MeRIP‐seq data (GSE61995). Bars represent the mean ± SD from n = 3 independent experiments. ^*^ *p* < 0.05, ^**^ *p* < 0.01 by two‐tailed Student's t‐test.

To validate this hypotheses, immunofluorescence (IF) and co‐localization analysis were performed to detect ELL‐mCherry and METTL3, revealing a strong co‐localization in the nucleus of reprogramming cells (Figure 6B). Co‐immunoprecipitation (Co‐IP) further confirmed the interaction between ELL and METTL3 (Figure 6C). To investigate the role of SEC component ELL in regulating m^6^A, we either overexpressed or interfered ELL and observed corresponding increases or decreases in global m^6^A levels of total RNA (Figure 6D). Subsequently, we inhibited SEC at the early stage and assessed the binding of METTL3 to RNA Pol II. Co‐IP assay showed that early inhibition of SEC, without affecting METTL3 protein levels, significantly disrupted the interaction between METTL3 and RNA Pol II (Figure 6E, Figure S5G). ChIP‐qPCR assays were conducted and further confirmed the co‐occupancy of METTL3 and ELL at the promoters of Myc and Klf4 (Figure 6F). Moreover, early SEC inhibition impaired the occupancy of both METTL3 and ELL at these promoters (Figure 6F). These results indicate that, during the early stage of iPSC reprogramming, SEC recruits METTL3 to and co‐occupies the promoters of the Klf4 and Myc loci. Finally, to determine whether SEC and METTL3 regulate m^6^A deposition on shared transcripts, we compared transcripts with reduced m^6^A modification following SEC inhibition and METTL3 knockout (GSE61995) and found that more than 50% overlapped (Figure 6G, Figure S5H) [51]. In conclusion, our data demonstrate that in the early stage of reprogramming, SEC recruits and interacts with METTL3 at the promoters of Klf4 and Myc, thereby regulating m^6^A deposition on their transcripts.

### 2.7 SEC Promotes Transcription Pause Release at Klf4 and Myc via Cooperation With METTL3

Here, we further explore whether SEC facilitates transcription pause release of Klf4 and Myc through interaction with METTL3. We first examined the role of METTL3‐mediated m^6^A in iPSC reprogramming. Although some studies have explored the role of m^6^A modification in reprogramming, its precise regulatory functions and underlying mechanisms remain a matter of debate [20, 24]. Here, we used the reported METTL3 inhibitor STM2457 (15 µm for 48 h) [52] to specifically inhibit its m^6^A methyltransferase activity without altering the expression of SEC components (Figure S6A,B). STM2457 was administered at different reprogramming stages, and reprogramming efficiency was assessed at day 10 (Figure 7A). AP staining showed that all treatment strategies significantly impaired reprogramming efficiency. Notably, once METTL3 inhibition encompassed the early stage of reprogramming (D0‐D4), it caused irreversible impairment of reprogramming efficiency (Figure 7A). Based on cellular morphology, we observed that early inhibition of METTL3 also blocked reprogramming, as cells failed to undergo epithelial transition or form iPSC colonies (Figure S6C). We further investigated and found that early inhibition of METTL3 (D0‐D4) markedly reduced reprogramming efficiency, whereas inhibition at the mid‐late stage (D6‐D10) significantly enhanced it (Figure 7B). These findings uncover a stage‐specific role for METTL3 in iPSC reprogramming: promoting reprogramming in the early stage, but suppressing it in the mid‐late stage. This dual functionality may explain the conflicting reports regarding METTL3‐mediated m^6^A in reprogramming and suggests that the SEC‐METTL3 pathway may play a critical role primarily during the early stage, whereas the SEC likely act through alternative mechanisms at later stages.

**FIGURE 7:**
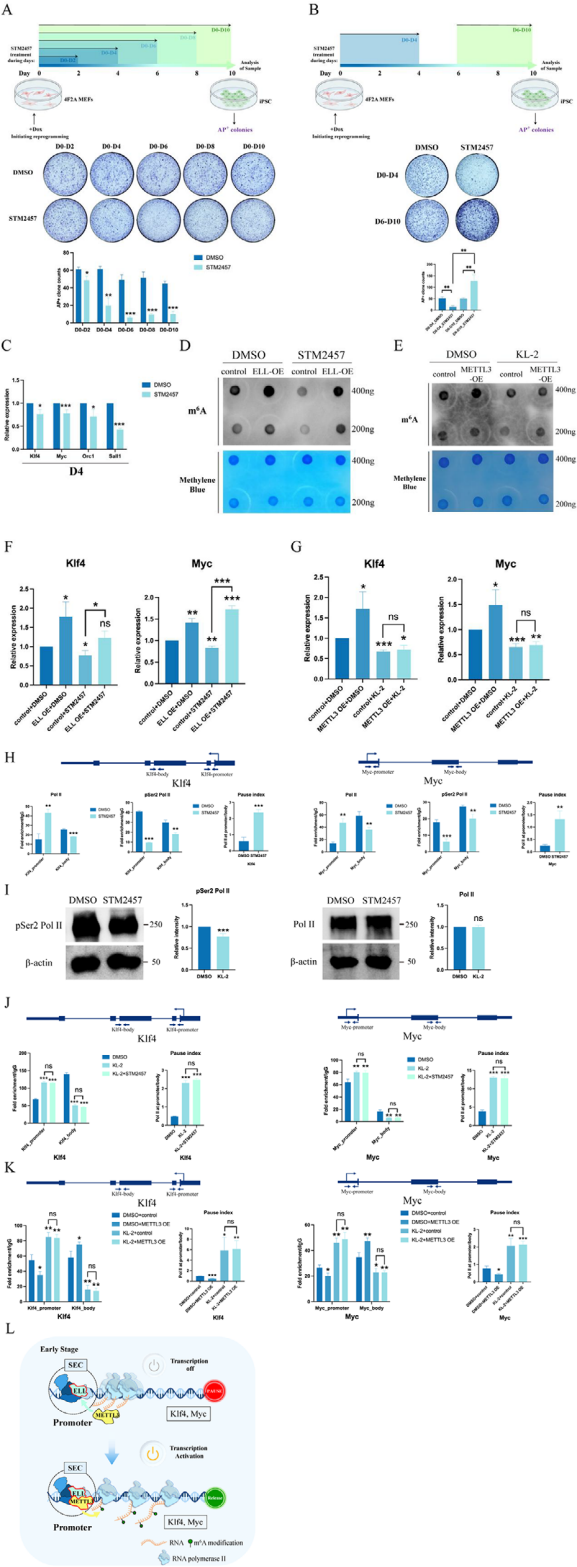
SEC cooperates with METTL3 to jointly promote transcription pause release and activates Klf4 and Myc expression. (A) AP staining on D10 shows iPSC colony formation following STM2457 treatment (middle), based on the treatment strategy illustrated (top). The histogram quantifies the number of AP^+^ colonies (bottom). (B) AP staining on D10 shows iPSC colony formation following STM2457 treatment during either the early stage (D0–D4) or the mid‐late stage (D6–D10) of reprogramming (middle). The schematic diagram illustrates the treatment strategy (top). The histogram quantifies the number of AP^+^ colonies (bottom). (C) qPCR shows the expression of SEC target genes (Klf4, Myc, Orc1, and Sall1) upon STM2457 treatment during the early reprogramming stage (D0–D4). (D, E) RNA dot blot shows the restoration of global m^6^A levels in total RNA by ELL OE under STM2457 treatment from D0 to D4 (D) or by METTL3 OE following KL‐2 treatment at D2 (E) in D4 reprogramming cells. Methylene blue was used as a loading control. The original blots are provided in Files S3. (F, G) qPCR analysis shows the restoration of Klf4 and Myc expression by ELL OE under STM2457 treatment from D0 to D4 (F) or by METTL3 OE following KL‐2 treatment at D2 (G) in D4 reprogramming cells. (H) ChIP‐qPCR analysis showing RNA Pol II and pSer2 RNA Pol II enrichment at the promoter and gene body of Klf4 and Myc in D4 reprogramming cells under early STM2457 treatment (D0–D4) (left). Right: Pause index for each gene as indicated. (I) Western blot showing the total protein level of pSer2 RNA Pol II and RNA Pol II in D4 reprogramming cells following early STM2457 treatment. The bar plot shows statistical results of gray value. The original blots are provided in Files S3. (J) ChIP‐qPCR showing RNA Pol II occupancy at the promoter and gene body of Klf4 and Myc under DMSO, D2_KL‐2, and combined D2_KL‐2 + early STM2457 treatment (left). Pause index for each gene as indicated (right). (K) CUT&RUN showing RNA Pol II occupancy at the promoter and gene body of Klf4 and Myc in D4 reprogramming cells following DMSO or KL‐2 treatment at D2, with or without METTL3 OE (left). Pause index for each gene as indicated (right). (L) Schematic model illustrating the mechanism by which the SEC facilitates iPSC reprogramming during the early stage through cooperation with METTL3. Upon initiation of reprogramming, the SEC component ELL recruits and interacts with the m^6^A methyltransferase METTL3 at the promoters of the representative key reprogramming genes, including Klf4 as well as Myc, thereby promoting m^6^A deposition and triggering the rapid release of pre‐established paused RNA Pol II, which in turn induces efficient transcriptional activation. Bars represent the mean ± SD from n = 3 independent experiments. ns: not significant, ^*^ *p* < 0.05, ^**^ *p* < 0.01, ^***^ *p* < 0.001 by two‐tailed Student's t‐test.

Subsequently, we examined the regulatory effect of METTL3 on the representative SEC target genes, such as Klf4 and Myc, during the early stage of reprogramming. Consistent with SEC inhibition, qPCR revealed a similarly significant downregulation of Klf4, Myc, and other SEC target genes following early inhibition of METTL3 (Figure 7C). These results therefore suggest that, during the early stage of reprogramming, the SEC may cooperate with METTL3 to activate the expression of SEC‐target genes, such as Klf4 and Myc. To test this hypothesis, we treated reprogramming cells with KL‐2 or STM2457 during the early stage and assessed whether overexpression of ELL, a component of the SEC, or METTL3 could respectively restore these effects (Figure S6D). We first examined the changes in global m^6^A level of total RNA. Early STM2457 treatment significantly reduced global m^6^A levels, which were partially alleviated by ELL overexpression (Figure 7D). Given that STM2457 inhibits METTL3 enzymatic activity, the partial alleviation mediated by ELL overexpression may be associated with enhanced recruitment of residual catalytically active METTL3, which is not completely blocked after STM2457 treatment (Figure S6A), at relevant loci, rather than direct restoration of METTL3 enzymatic function. In contrast, METTL3 overexpression, capable of increasing m^6^A levels under normal conditions, failed to restore m^6^A levels observed after KL‐2 treatment (Figure 7E). Next, we investigated whether the transcription of Klf4 and Myc could be restored. Similarly, ELL overexpression partially restored Klf4 and Myc expression levels reduced by early STM2457 treatment (Figure 7F). However, although METTL3 overexpression promotes Klf4 and Myc transcription under normal conditions, it still failed to restore their expression following early KL‐2 treatment (Figure 7G). These findings suggest that during the early stage of reprogramming, the SEC can enhance Klf4 and Myc transcription through functional cooperation with METTL3.

To further test the possibility that the SEC facilitates transcription pause release at Klf4 and Myc loci through additional cooperation with METTL3, we first explored whether METTL3 can regulate transcription pause release in mammals. We reanalyzed published ChIP‐seq data for RNA Pol II in HeLa cells with METTL3 knockdown (HRA002494) and found that METTL3 knockdown (shMETTL3) markedly increased RNA Pol II enrichment near the TSS, accompanied by reduced occupancy along gene bodies (Figure S6E). TR and TM analyses further confirmed that METTL3 knockdown significantly enhanced transcription pause in HeLa cells (Figure S6F–H). We also reanalyzed published NET‐seq data from METTL3 knockout mESC (GSE133582), which revealed increased nascent transcripts near the TSS and reduced signals along the gene body in METTL3 knockout cells (Figure S6I) [53]. Additionally, TM analysis showed that METTL3 knockout led to a substantial increase in transcription pause across a large number of genes (Figure S6J,K). These results suggest that METTL3 likely also facilitates transcription pause release in mammals. Subsequently, ChIP‐qPCR was performed to investigate whether METTL3 regulates RNA Pol II pause release at the Klf4 and Myc loci, two representative SEC target genes, during the early stage of iPSC reprogramming (Figure 7H). In line with the ChIP‐seq and NET‐seq results, early STM2457 treatment markedly increased RNA Pol II enrichment at the promoters of Myc and Klf4, while significantly reducing its presence along their gene bodies (Figure 7H). ChIP of pSer2 RNA Pol II further revealed a significant reduction at both the promoter and gene body regions of Klf4 and Myc following early STM2457 treatment (Figure 7H). The pause index also indicated a significant increase in transcription pause at Klf4 and Myc upon early METTL3 inhibition (Figure 7H). It also showed that early STM2457 treatment reduced total pSer2 RNA Pol II levels without affecting total RNA Pol II expression (Figure 7I). Additionally, nascent RNA synthesis assays revealed that early METTL3 inhibition also significantly decreased nascent RNA transcription (Figure S6L). These results confirm that during the early stage of reprogramming, METTL3 also contributes to transcription pause release at the SEC‐target genes, such as Klf4 and Myc loci.

Finally, we investigated whether SEC can cooperate with METTL3 to facilitate the release of pre‐established paused RNA Pol II at Klf4 and Myc loci. We identified genes expressed at D4 from our RNA‐seq data and classified them according to SEC and METTL3 occupancy. SEC occupancy was defined as the co‐occupancy of AFF4 and ELL, based on published ChIP‐seq datasets for AFF4 (GSE30267) and ELL (GSE32120), whereas METTL3 occupancy was determined using a published METTL3 ChIP‐seq dataset (GSE154135) (Figure S7A). This analysis identified 648 expressed genes co‐occupied by SEC and METTL3 (Figure S7B,C). These co‐occupied genes included Klf4 and Myc, two representative SEC target genes that also exhibited METTL3 occupancy at their respective loci (Figure S7D,E). Reanalysis of published RNA‐seq data from METTL3‐knockout cells (GSE325527), together with our RNA‐seq data obtained following SEC inhibition, showed that approximately 40% of the SEC–METTL3 co‐occupied genes were downregulated under both SEC inhibition and METTL3‐knockout conditions (Figure S7F,G). Furthermore, we analyzed RNA Pol II CUT&Tag data obtained after early KL‐2 treatment and published NET‐seq data from METTL3‐knockout cells (GSE133582) to assess transcription pause release across the different gene groups. TR and TM analyses indicated that both early KL‐2 treatment and METTL3 knockout increased transcription pause among the expressed genes co‐occupied by SEC and METTL3 (Figure S7H–J). Collectively, these findings suggest that SEC and METTL3 may jointly regulate RNA Pol II pause release.

To further determine whether SEC and METTL3 regulate RNA Pol II pause release at the Klf4 and Myc loci through a shared functional pathway during early iPSC reprogramming, we performed combined SEC and METTL3 inhibition. Here, SEC and METTL3 were simultaneously inhibited by KL‐2 and STM2457 during the early stage of reprogramming. If these two factors regulated RNA Pol II pause release at Klf4 and Myc loci independently, combined inhibition would be expected to produce a greater increase in promoter‐proximal pause than inhibition of either factor alone. Conversely, if SEC and METTL3 functioned within the same regulatory pathway, simultaneous inhibition would be expected to produce little or no additional increase in promoter‐proximal pausing compared with inhibition of either factor alone. ChIP‐qPCR assay of RNA Pol II showed that early KL‐2 treatment significantly increased transcription pause at Klf4 and Myc. However, co‐treatment with KL‐2 and STM2457 did not further enhance the pause level (Figure 7J), suggesting that SEC and METTL3 function in the same pathway to regulate transcription pause release at Klf4 and Myc. Furthermore, METTL3 overexpression was used as another strategy to further test this regulatory model. Under conditions of SEC inhibition, if SEC and METTL3 regulated RNA Pol II pause release at the Klf4 and Myc loci through independent pathways, METTL3 overexpression would be expected to at least partially restore the defect in pause release. However, RNA Pol II Cleavage Under Targets and Release Using Nuclease (CUT&RUN) analysis showed that, under control conditions, METTL3 overexpression significantly enhanced the release of paused RNA Pol II at the Klf4 and Myc loci, supporting a role for METTL3 in promoting pause release at these loci during early reprogramming (Figure 7K). By contrast, under KL‐2 treatment, METTL3 overexpression failed to restore the defect in RNA Pol II pause release at either the Klf4 or the Myc locus (Figure 7K). These findings suggest that early SEC inhibition may disrupt the scaffolding function of SEC required for local METTL3 recruitment at Klf4 and Myc loci, thereby impairing METTL3‐mediated m^6^A deposition and RNA Pol II pause release through a shared SEC–METTL3 regulatory pathway. Together, these results support a model in which SEC cooperates with METTL3 to jointly facilitate RNA Pol II pause release at the Klf4 and Myc locus.

Collectively, our findings reveal that the SEC drives the reprogramming of MEFs into iPSCs by promoting early activation of core reprogramming genes, including Klf4 and Myc, and that the SEC additionally cooperates with METTL3 to facilitate the rapid release of pre‐established paused RNA Pol II at these loci (Figure 7L).

### 2.8 SEC Drives ESC Differentiation Into Neuroectoderm by Activating Core Neural Genes

Since iPSC reprogramming represents a reversal of normal differentiation, we next asked whether SEC also plays a vital role in driving cell identity transition during normal ESC differentiation. First, we reanalyzed published RNA Pol II ChIP‐seq data (GSE261039) to assess dynamic transcription pause during ESC differentiation [54]. TR analysis revealed that, similar to the reprogramming process, transcription pause gradually increases during the course of differentiation (Figure 8A,B). TM analysis identified early rapidly released genes (Cluster I) and gradually paused genes (Cluster IV) from 0 to 72 h of retinoic acid (RA) treatment (Figure 8C, Figure S8A). Using published RNA‐seq data of differentiated cells at corresponding time points (GSE185889), we identified activated genes and found that approximately 31.1% showed increased pause, while 14.8% displayed rapid release (Figure 8D,E, Figure S8B) [55]. GO analysis of the two gene groups confirmed that both are enriched for kinds of lineage development‐related genes (Figure S8C). In conclusion, these data suggest that similar to iPSC reprogramming, the initiation of differentiation activates lineage‐specific genes either by rapid release pre‐established paused RNA Pol II or via a coupled pause‐and‐release mechanism. Furthermore, these findings highlight that cell identity transitions, whether in ESC differentiation or iPSC reprogramming, critically rely on transcription release mechanisms to activate key genes.

**FIGURE 8:**
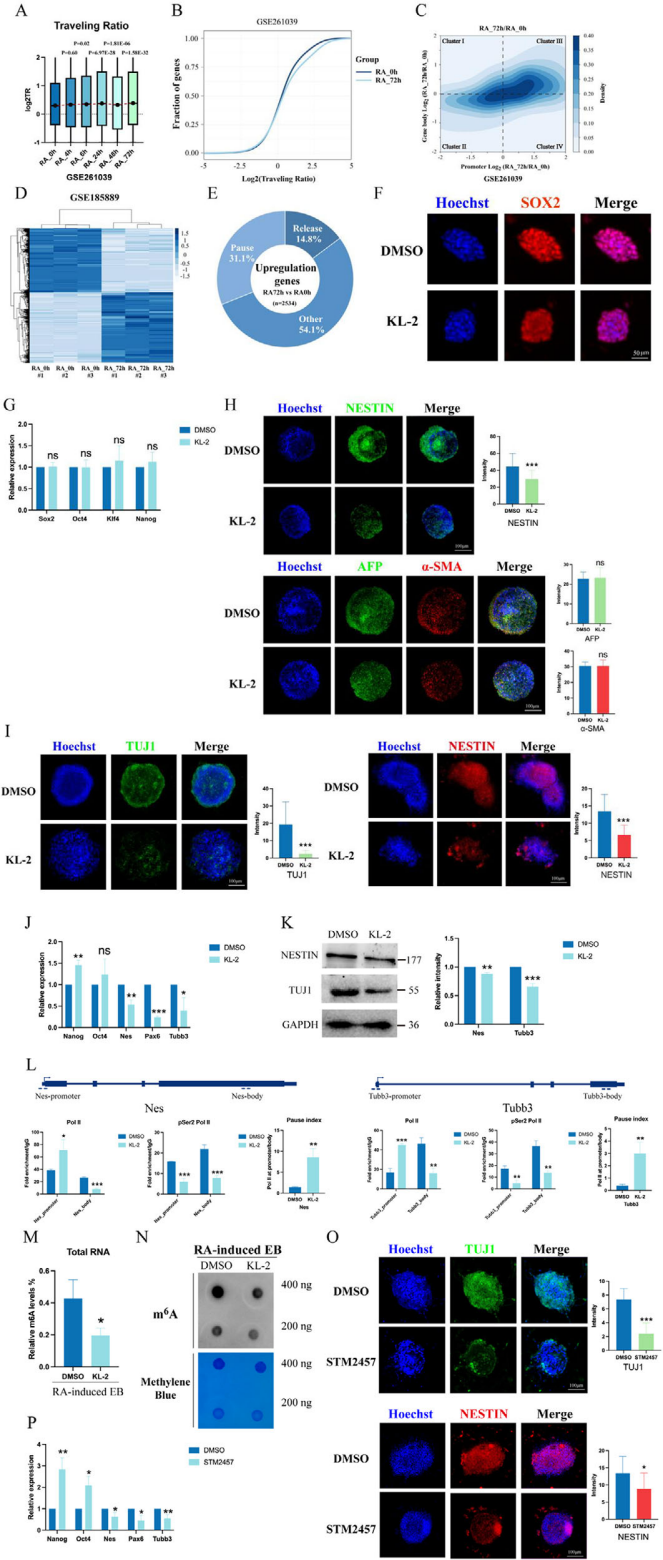
Inhibition of SEC specifically impairs the differentiation of ESC to neuroectoderm lineage. (A) Box plot showing log_2_TR values at the indicated time points during ESC differentiation, based on published RNA Pol II ChIP‐seq data (GSE261039). *p*‐values were calculated by t‐test and are labeled above each box. (B) ECDF plot of log_2_TR at RA_0 h and RA_72 h of ESC differentiation. (C) 2D topographic map from TM analysis showing differential enrichment of RNA Pol II at promoter and gene body regions between RA_72 h and RA_0 h. (D) Heatmap displaying differentially expressed genes between RA_0 h and RA_72 h, based on published RNA‐seq data (GSE185889) (*p* < 0.05 and |Log_2_FC| > 1.5). (E) Pie chart illustrating the proportions of pause‐increased genes, release‐increased genes, and the others among genes upregulated at RA_72 h compared to RA_0 h (n = 2534). (F) Immunofluorescence staining of pluripotency marker SOX2 in undifferentiated mESCs treated with KL‐2. Scale bar = 50 µm. (G) qPCR analysis showing the expression of pluripotency‐associated genes (Sox2, Oct4, Klf4 and Nanog) in undifferentiated mESCs treated with KL‐2. (H) Immunofluorescence images showing expression of lineage‐specific markers following spontaneous differentiation: NESTIN (neuroectoderm), α‐SMA (mesoderm), and AFP (endoderm) in DMSO versus KL‐2‐treated groups. Histograms show average fluorescence intensity per marker. Scale bar = 100 µm. (I) Immunofluorescence images showing TUJ1 and NESTIN expression following directed neuroectodermal differentiation of mESCs in the DMSO‐ and KL‐2‐treated groups. Histograms depict average fluorescence intensity for each marker. Scale bar = 100 µm. (J) qPCR showing transcription of pluripotency genes (Nanog and Oct4) and neuronal lineage genes (Nes, Pax6, and Tubb3) in DMSO and KL‐2 groups. (K) Western blot analysis showing levels of neuronal proteins NESTIN and TUJ1 in differentiated neuronal lineage cells. The bar plot shows statistical results of gray value. The original blots are provided in Files S3. (L) ChIP‐qPCR showing the occupancy of RNA Pol II and pSer2 RNA Pol II at the promoter and gene body regions of Nes and Tubb3 in neuronal lineage cells (left). Right: The pause index for each gene. (M, N) Global m^6^A levels in total RNA from cells undergoing directed neuroectodermal differentiation with or without KL‐2 treatment were assessed by ELISA (M) and RNA dot blot (N). Methylene blue was used as a loading control. The original blot is provided in Files S3. (O) Immunofluorescence staining showing TUJ1 and NESTIN expression following directed neuroectodermal differentiation in the DMSO‐ and STM2457‐treated groups. Histogram quantifies average fluorescence intensity. Scale bar = 100 µm. P: qPCR showing the expression of pluripotency markers (Nanog and Oct4) and neuroectodermal genes (Nes, Pax6, and Tubb3) in DMSO and STM2457 groups. Bars represent the mean ± SD from n = 3 independent experiments. ns: not significant, ^*^ *p* < 0.05, ^**^ *p* < 0.01, ^***^ *p* < 0.001 by two‐tailed Student's t‐test.

We next investigated whether SEC also functions as a key regulator of ESC differentiation by promoting transcription pause release at key genes. ESCs were treated with KL‐2 (10 µM, 12 h) under an undifferentiated state, and found that it had minimal effects on ESC colony morphology, pluripotency gene expression, or proliferation (Figure 8F,G, Figure S8D–G). These results suggest that SEC deficiency hardly affect the basic identity or survival of ESCs prior to differentiation. We then induced spontaneous ESC differentiation and examined the role of SEC by treating cells with KL‐2. KL‐2 was treated at 48 h after differentiation initiation. Interestingly, inhibition of SEC had no significant effect on mesoderm (α‐SMA as the hallmarker) or endoderm (AFP as the hallmarker) differentiation, but markedly reduced neuroectoderm (NESTIN as the hallmarker) differentiation efficiency (Figure 8H). These findings suggest that SEC may specifically regulate the differentiation of ESCs into neuroectoderm. To validate this hypothesis, we employed a RA‐induced directed differentiation system to drive ESC differentiation toward the neuroectoderm lineage. KL‐2 was administered at the initiation of RA treatment (RA_D0). Consistent with our hypothesis, SEC inhibition significantly reduced the expression of representative neural lineage marker, including TUJ1 and NESTIN, in differentiated cells, indicating impaired acquisition of neural cell identity (Figure 8I). qPCR analysis further revealed that inhibition of SEC during the differentiation impaired the activation of neural genes (Nes, Pax6, and Tubb3) and the downregulation of pluripotency genes (Nanog and Oct4) in differentiated neuronal lineage cells (Figure 8J). Western blot analysis also confirmed decreased protein levels of the representative neural lineage markers, including NESTIN and TUJ1 (Figure 8K). Moreover, to further validate the requirement of SEC in neural lineage differentiation, established iPSCs were also induced toward neuroectodermal differentiation under SEC inhibition to assess the role of SEC in this cell identity transition. Consistent with the observations in ESC differentiation, SEC inhibition also markedly impaired iPSC differentiation and the acquisition of neural cell identity, as indicated by decreased expression of neural lineage markers, including TUJ1 and NESTIN (Figure S8H). Collectively, these results suggest that SEC promotes the transition of pluripotent stem cells, including ESCs and iPSCs, toward neuroectodermal lineages by regulating the expression of neural genes, such as Nes and Tubb3.

We next investigated whether SEC activates the transcription of representative neural genes, such as Nes and Tubb3, by facilitating the release of paused RNA Pol II. ChIP‐qPCR assay for RNA Pol II was conducted after KL‐2 treatment. The results revealed that SEC inhibition led to increased RNA Pol II accumulation at the promoter of Nes and Tubb3, accompanied by reduced occupancy within the gene body (Figure 8L). Meanwhile, pSer2 RNA Pol II levels were markedly reduced at both the promoter and gene body of Nes and Tubb3 following SEC inhibition (Figure 8L). Pause index analysis further confirmed the increased transcription pause at Nes and Tubb3 upon SEC inhibition (Figure 8L). These results indicate that, during neuroectodermal differentiation, SEC promotes the release of paused RNA Pol II at neural lineage genes, including Nes and Tubb3, thereby activating transcription. Since SEC was shown to regulate m^6^A deposition during iPSC reprogramming, we hypothesized that it might play a similar role during ESC differentiation into neuroectoderm. ELISA and RNA dot blot assays performed after KL‐2 treatment showed that SEC inhibition also impaired global m^6^A levels in total RNA during ESC‐to‐neuroectoderm differentiation, suggesting that SEC‐mediated m^6^A regulation may represent a broader mechanism rather than a context‐specific effect (Figure 8M,N).

Moreover, we treated STM2457 (15 µm for 48 h) at the initiation of RA treatment (RA_D0). STM2457 treatment during ESC direct differentiation into neuroectoderm also significantly impaired differentiation efficiency, accompanied by failed repression of pluripotency genes and activation of neural genes (Figure 8O,P). Reanalysis of previous NET‐seq data from METTL3 knockout mESCs further revealed that most nascent transcripts were stalled near the promoters of Nes and Tubb3, with reduced levels in the gene body (Figure S8I). These findings suggest that METTL3‐mediated m^6^A probably also cooperates with SEC to promote key gene activation, thereby facilitating ESC differentiation into neural lineage cells.

## 3 Discussion

Although the cell identity transition has recently become a prominent topic linked to numerous biological processes, the multilayered regulatory network, particularly the core factors commonly involved across different types of transitions, remains poorly understood (6). Here, we identify the SEC, a key regulator of transcription pause release, as a central driver in two opposing cell identity transitions: iPSC reprogramming and ESC differentiation.

In the process of iPSC reprogramming, the SEC plays an indispensable role in driving the cell identity transition from MEFs to iPSCs, particularly at the early stage. At the early stage, the SEC regulates multiple key reprogramming events, including maintenance of the cell cycle and activation of the pluripotency network, by facilitating transcription pause release and thereby increasing the expression of the key reprogramming genes, including Klf4 and Myc. Previous studies have shown that iPSC reprogramming involves two major transcriptional waves [27]. The first wave primarily includes genes involved in cell adhesion as well as metabolism, and is driven by Klf4 and Myc. The second wave includes pluripotency‐related genes and is driven by Oct4, Sox2, and Klf4 [27]. Intriguingly, our results indicated that Klf4 and Myc undergo rapid release of pre‐established paused RNA Pol II and increased transcription during the early stage of reprogramming, whereas Oct4 and Sox2, which lack pre‐established paused RNA Pol II, gradually establish transcription pause before releasing paused RNA Pol II and activating transcription. It therefore suggests that the initiation of transcriptional waves might be tightly regulated by transcription pause release mechanisms, and SEC might function as a trigger for the first transcriptional wave. Further, it also implies that in the mid‐late stage, SEC may facilitate the expression of core genes, such as Oct4 and Sox2, by similarly regulating transcription pause release or through other unidentified mechanisms, thereby contributing to the second transcriptional wave. However, in Figure S2E, we observed that some early‐activated genes with rapid transcriptional release did not exhibit significant downregulation upon SEC inhibition. It therefore suggests that additional elongation factors may contribute to the initiation of transcriptional waves and thereby participate in regulating cell identity transitions during iPSC reprogramming by modulating transcription pause release.

In Figure 1F, we overexpressed ELL in an attempt to restore the impaired iPSC reprogramming efficiency caused by early KL‐2 treatment; however, only a modest rescue effect was observed. This limited restoration may be explained by the fact that ELL represents only one functional component of SEC, a multi‐subunit transcriptional regulatory complex. Therefore, ELL overexpression alone is unlikely to fully substitute for the function of the entire SEC complex and can only partially alleviate the impaired reprogramming efficiency. It is noteworthy that this partial alleviation should not be interpreted as evidence that ELL is the only SEC component regulating iPSC reprogramming, as other SEC components, including AFF4, ELL2, and ENL, may also be involved in SEC‐regulated iPSC reprogramming. Future studies focusing on the specific roles of other SEC components beyond ELL during iPSC reprogramming will help further dissect the detailed mechanisms by which the SEC regulates cell identity transition during iPSC reprogramming.

In addition, it is also worth mentioning that the stage‐specific regulation of METTL3‐mediated m^6^A in the reprogramming. As is well known, METTL3‐mediated m^6^A is a complex and versatile epigenetic modification involved in a wide range of biological processes [15]. It is likely that m^6^A modifications impair key biological events during the mid‐late stage of reprogramming, potentially by reducing transcript stability, interfering with proper splicing, or via other yet unidentified mechanisms. We propose that METTL3‐mediated m^6^A does not act as a strictly positive or negative regulator at specific stages, but rather exerts a dual function throughout reprogramming, just with its total function biased toward positive regulation in the early stage and negative regulation in the mid‐late stage. Therefore, identifying upstream regulatory factors that determine the directional balance of METTL3‐mediated m^6^A function in the iPSC reprogramming may represent a future research focus and could be key to understanding the mechanisms underlying the stage‐specific regulation of m^6^A modification.

While SEC is classically thought to promote transcriptional pause release through its P‐TEFb‐mediated phosphorylation of the RNA Pol II CTD and the pause factors DSIF and NELF, our study proposes an additional pathway in which the SEC can collaborate with METTL3 to regulate m^6^A modification at Klf4 and Myc loci. Although this pathway has thus far only been observed during the early stage of iPSC reprogramming, it is worthwhile to investigate in depth whether it represents a general mechanism by which the SEC functions in other biological contexts. Here, we found that SEC facilitates local METTL3 recruitment to the Klf4 and Myc loci through its scaffolding function, thereby promoting m^6^A deposition at these loci. ELL overexpression partially restored m^6^A levels under STM2457 treatment, possibly by enhancing the recruitment of residual catalytically active METTL3. By contrast, METTL3 overexpression failed to restore the reduced m^6^A levels under KL‐2 treatment. Given that early SEC inhibition reduced METTL3 occupancy at the Klf4 and Myc loci (Figure 6F), SEC serves a scaffolding role in local METTL3 recruitment. Thus, although total METTL3 protein abundance was unchanged following KL‐2 treatment, impaired SEC scaffolding function may have limited the local recruitment of METTL3 required for efficient m^6^A deposition. Notably, although residual AFF4 protein also remained detectable after KL‐2 treatment, its presence may not cooperate with overexpressed METTL3 to restore the reduced m^6^A levels. One possible explanation is that SEC may support METTL3‐mediated m^6^A deposition through mechanisms beyond its scaffolding role. Although our study primarily focuses on SEC‐dependent recruitment of METTL3 to the Klf4 and Myc loci as a mechanism underlying SEC–METTL3 cooperation, other SEC‐mediated mechanisms may also contribute to METTL3 function. For instance, a previous study reported that METTL3 interacts with RNA Pol II, thereby facilitating its access to nascent RNA and co‐transcriptional m^6^A deposition [56]. Our results showed that early SEC inhibition impaired the interaction between METTL3 and RNA Pol II (Figure 6E), suggesting that SEC may additionally support METTL3‐mediated m^6^A deposition by facilitating the association between METTL3 and RNA Pol II. Thus, disruption of these additional, non‐scaffolding functions of SEC may explain why METTL3 overexpression, which cannot compensate for their loss, was insufficient to restore m^6^A levels under KL‐2 treatment. In addition, our study also showed that SEC‐dependent m^6^A regulation varied across the transcript regions. Although m^6^A levels at the gene‐body and 3′‐region sites were generally reduced following early SEC inhibition, the promoter‐proximal sites exhibited divergent responses: m^6^A levels at the promoter‐proximal site in Klf4 decreased, whereas those at the corresponding site in Myc showed a slight but statistically nonsignificant increase. These findings suggest that SEC‐dependent m^6^A regulation may be influenced by additional factors, such as local transcript features, transcription kinetics, RNA processing states, and the recruitment or accessibility of m^6^A writer and eraser machinery. Future high‐resolution spatiotemporal m^6^A mapping will be valuable for dissecting these regulatory mechanisms.

Previous studies have primarily focused on the role of m^6^A on the post‐transcriptional RNA, whereas its regulatory functions in transcriptional program are now gaining increasing attention. Here, METTL3‐mediated m^6^A was shown to promote transcription pause release of representative SEC‐target genes, including Klf4 and Myc, during early stage of iPSC reprogramming, with the assistance of SEC. Interestingly, a similar role of m^6^A in transcription pause release has also been observed in *Drosophila* [17]. Hence, it suggested that the regulation of transcription pause release by METTL3‐mediated m^6^A may represent a conserved mechanism across diverse biological contexts and species. However, the mechanism by which METTL3‐mediated m^6^A regulates transcription pause release remains incompletely understood. Recent studies suggest that m^6^A deposition on R‐loops appears to be associated with transcriptional pause release. Upon transcriptional pause, R‐loops can block RNA Pol II progression and reinforce the paused state [57, 58]. However, m^6^A modification deposited by METTL3 on R‐loops can be recognized by m^6^A reader proteins, thereby recruiting various “cleaner” factors to resolve R‐loops and promote the release of paused RNA Pol II [16, 57, 59, 60, 61, 62]. Together, these studies suggest that m^6^A‐mediated R‐loop resolution may serve as a key trigger for transcription pause release, and that m^6^A deposited by the SEC–METTL3 pathway may localize to R‐loops to facilitate this process. Future studies to explore and validate this potential pathway will be valuable for filling up the detailed mechanism gap of how METTL3‐mediated m^6^A regulates transcription pause release and uncovering the complete roles of SEC and METTL3 in cell identity transitions.

For ESC differentiation, similar mechanisms of rapid release pre‐established paused RNA Pol II and a coupled pause and release were also observed in both ectoderm‐, mesoderm‐, and endoderm‐related genes in our studies. Given the sequential divergence of the three germ layers, the timing and magnitude of transcriptional pause release regulation may influence the outcome of ESC differentiation by modulating the expression of lineage‐specific genes. Interestingly, we here found that SEC primarily regulates the differentiation of ESCs into neuroectoderm, but not into mesoderm or endoderm, suggesting that SEC may function as a molecular determinant in directing cell fate. Meanwhile, other elongation factors may compensate for SEC specifically in mesendodermal lineage cells to facilitate paused RNA Pol II release. Previous studies have shown that another transcription elongation factor, BRD4, is essential for mesendoderm specification [63]. Moreover, although the SEC and BRD4 have similar functions in regulating transcription pause release, they have been reported to function independently in this process [64]. As a result, these results suggest that the functional divergence between SEC and BRD4 in regulating transcription pause release of lineage‐specific genes may be a key determinant of ESC differentiation direction. During differentiation toward neuroectoderm, our study demonstrates that SEC regulates m^6^A, which is also able to promote ESC differentiation into the neuroectoderm lineage. Thus, a potential SEC‐METTL3 regulatory pathway may also play a key role in activating the transcription of neuroectoderm‐specific genes and promoting differentiation.

It is noteworthy that, in this study, Klf4/Myc and Nestin/Tubb3 were used as representative SEC‐responsive genes associated with iPSC reprogramming and ESC‐to‐neural lineage differentiation, respectively. These genes should not be simply interpreted as the sole downstream effectors responsible for the global phenotypes caused by SEC inhibition. Both iPSC reprogramming and ESC‐to‐neural lineage differentiation require the coordinated activation of broad and temporally ordered transcriptional programs. Therefore, SEC inhibition likely affects multiple genes and regulatory pathways beyond the representative targets examined here. Future studies exploring additional SEC‐target genes in these two cell identity transitions will be valuable for achieving a deeper understanding of the SEC regulatory network.

Previous studies have proposed that RNA Pol II pause is dispensable for cell survival under steady‐state conditions but essential during periods of cell identity specification and fate transitions [65]. Likewise, our study further indicates that SEC is also non‐essential for basic cell survival in either MEFs or ESCs under steady‐state conditions, but is indispensable for both iPSC reprogramming and ESCs differentiation, highlighting the requirement of transcription pause release in cell identity transition. These findings support the hypothesis that SEC may serve as a key driver in additional cell identity transitions beyond those explored here, including early embryonic development, tissue transformation or regeneration after injury, and various disease initiation and progression. Exploring these issues would not only enhance our understanding of the mechanisms underlying various cell identity transitions, but also pave the way for developing SEC‐based diagnostic and therapeutic strategies for diseases associated with cell identity changes, such as tumorigenesis, viral infections, and infertility.

## 4 Conclusions

In summary, we identified SEC as a critical driver of cell identity transitions, including both iPSC reprogramming and ESC differentiation into neuroectoderm. During the early stage of iPSC reprogramming, SEC promotes the expression of core reprogramming genes, such as Klf4 and Myc, by facilitating the release of pre‐established paused RNA Pol II, thereby supporting key reprogramming events, including cell‐cycle progression and activation of the pluripotency network. Moreover, in the early stage, the SEC can additionally recruit and interact METTL3 to the promoter of Klf4 and Myc, and further promote m^6^A modification and transcription pause release at these loci. In contrast, early inhibition of SEC severely disrupts these processes, ultimately resulting in reprogramming failure (Figure 9, left). Similarly, during ESC differentiation toward the neuroectodermal lineage, SEC also specifically facilitates the release of paused RNA Pol II at the promoter of neural genes, including Nes and Tubb3, leading to their transcriptional activation. Additionally, SEC also regulates m^6^A deposition in differentiated cells, suggesting the existence of a potential SEC–METTL3 pathway. Likewise, loss of SEC function also strikingly impairs this process, thereby compromising the cell identity transition during neural differentiation (Figure 9, right).

**FIGURE 9:**
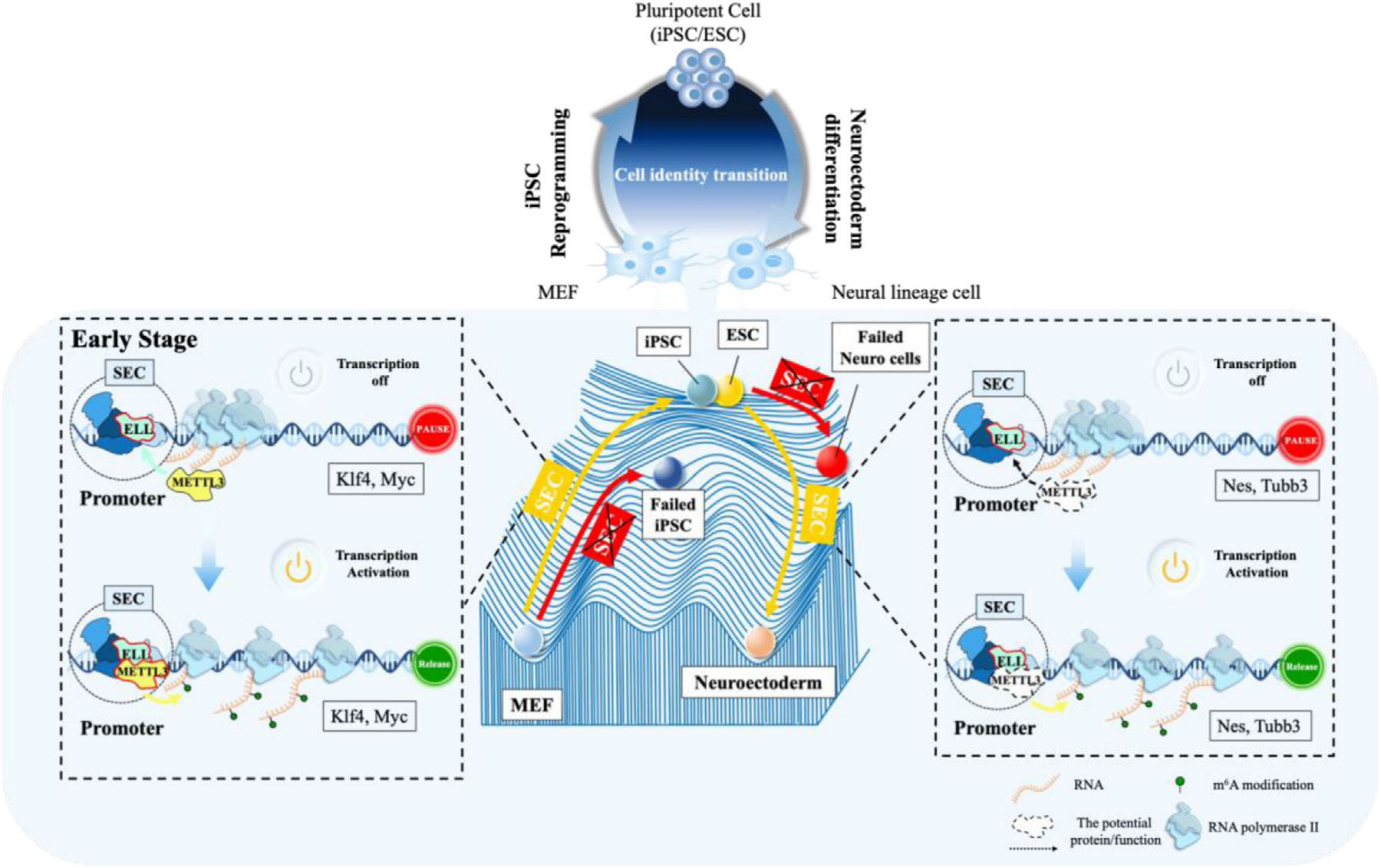
Model for the mechanism of SEC regulating cell identity transition. In this study, we employed two independent models of cell identity transition: MEF‐to‐iPSC reprogramming and ESC‐to‐neuroectodermal differentiation (top panel). The mechanism by which SEC regulates these transitions is illustrated below (bottom panel): In the early stage of iPSC reprogramming, SEC recruits and interacts with m^6^A writer METTL3 at the promoter of Klf4 and Myc, to form a SEC‐METTL3 functional complex. This complex can facilitate m^6^A deposition and trigger the rapid release of pre‐established paused RNA Pol II, thereby promoting efficient transcriptional activation of key reprogramming genes, including Klf4 and Myc. By cooperating with METTL3, SEC promotes the reprogramming of somatic cell identity in MEFs into pluripotent cell identity in iPSCs. Inhibition of SEC severely disrupts this process, resulting in reprogramming failure (left panel). During ESC differentiation into neuroectoderm, SEC similarly activates neuronal lineage genes, such as Nes and Tubb3, by promoting the release of paused RNA Pol II, thus facilitating the transition from ESCs with pluripotent cell identity to neural lineage cells with somatic cell identity. In this context, SEC also regulates m^6^A deposition and may function in cooperation with METTL3. SEC inhibition also impairs the transition of ESC to neuroectoderm (right panel).

## 5 Methods

### 5.1 Mice and Cell Culture

Female C57BL/6 and male DBA/2 mice were purchased from Vital River Laboratories (China). Male and female 4F2A mice, which express a doxycycline‐inducible polycistronic 4F2A cassette, were obtained from Jackson Laboratory (USA). MEF cells were isolated from E13.5 embryos obtained by crossing female C57BL/6 and male DBA/2 mice. 4F2A‐MEF cells were similarly derived from E13.5 embryos of 4F2A mice. The established iPSCs used in this study were generated by reprogramming 4F2A MEFs. The R1 mouse embryonic stem (ES) cell line was obtained from the American Type Culture Collection (ATCC).

MEFs and HEK293T cells were cultured in MEF medium (Dulbecco's Modified Eagle Medium (DMEM)/high glucose supplemented with fetal bovine serum (FBS, Biological Industries), MEM nonessential amino acids (NEAA, Gibco), L‐glutamine (Gibco), and penicillin‐streptomycin (Biological Industries)). 4F2A MEFs used for iPSC reprogramming were cultured in iPSC reprogramming medium (KnockOut DMEM (KO‐DMEM, Gibco) supplemented with KnockOut Serum Replacement (KOSR, Gibco), NEAA, L‐glutamine, penicillin‐streptomycin, sodium pyruvate (Gibco), LIF, 2‐mercaptoethanol, and 2 µg/mL doxycycline (DOX)). Undifferentiated R1 ESCs and iPSCs were cultured on a feeder layer of mitotically inactivated MEFs in ESC medium (DMEM/high glucose supplemented with FBS, LIF, sodium pyruvate, L‐glutamine, penicillin‐streptomycin, 2‐mercaptoethanol, and NEAA).

MEFs from 4F2A mice were used for MEF‐to‐iPSC reprogramming experiments. R1 mESCs and established miPSCs were used for differentiation experiments. These cell systems were used as distinct experimental models to investigate SEC function during different types of cell identity transitions. MEFs derived from C57BL/6 × DBA/2 mice were used for feeder preparation. HEK293T cells were used only for lentiviral packaging.

### 5.2 Plasmids Construction and siRNA Synthesis

The pLV[Exp]‐mCherry:T2A:Puro‐EF1A>mELL plasmid was purchased from VectorBuilder. The pLVX‐METTL3(mouse)‐Puro (P21800) was obtained from MiaoLingBio, China. The psPAX2, and pCMV ‐VSVG were acquired from Addgene. The shRNA was obtained from Focus company. The siRNA for AFF4 and ELL were synthesized by MIJIA company.

### 5.3 Virus Infection

HEK293T cells were transfected with the lentiviral packaging plasmids psPAX2 and pCMV‐VSVG, together with either overexpression plasmids, including pLV[Exp]‐mCherry:T2A:Puro‐EF1A>mELL and pLVX‐METTL3(mouse)‐Puro, or shRNA plasmids, including pFB‐9‐shNC (mouse) and pFB‐9‐shELL (mouse), using the Lipofectamine 3000 Transfection Kit. Lentivirus‐containing supernatants were collected at 48 and 72 h post‐transfection and used to infect target cells in the presence of polybrene for 48 h.

### 5.4 Generation of iPSCs and Alkaline Phosphatase (AP) Staining

4F2A MEFs were seeded onto mitomycin C‐treated MEF feeder layers pre‐plated in sixwell plates or 10‐cm dishes, and cultured in MEF medium supplemented with 2 µg/mL DOX for 3 days. The medium was then replaced with iPSC reprogramming medium containing 2 µg/mL DOX. Fresh medium was replenished daily. Inhibitors, including KL‐2 (MedChemExpress (Monmouth Junction, NJ, USA), HY‐123972) and STM2457 (MedChemExpress (Monmouth Junction, NJ, USA), HY‐134836), were added to the medium at the indicated time points. siRNAs were transfected into reprogramming cells using Lipofectamine3000 Transfection Kit. Reprogramming cells were collected at designated time points for analysis.

Reprogramming efficiency was evaluated on day 10 of induction using AP staining with the BCIP/NBT color development kit (Beyotime, C3206). AP‐positive colonies were quantified using ImageJ software.

### 5.5 ESC/iPSC Spontaneous Differentiation and Direct Differentiation to Neuronal Cells

For spontaneous differentiation, ESCs/iPSCs were dissociated into single cells using trypsin/EDTA and resuspended in ESC medium without LIF. The cell concentration was adjusted to approximately 5*10^^^5 cells/mL using 10 mL ESC medium without LIF. The cells were then replated into low‐attachment plates for differentiation, with medium changes every 3 days. Embryoid bodies (EBs) formed within 24 h under standard culture conditions (37°C, 5% CO_2_) and were cultured for 1 week before transitioning to MEF medium. After 10 days of differentiation, EBs were collected for further analysis.

To induce the direct differentiation of ESCs/iPSCs into neuronal cells, ESCs/iPSCs were first subjected to spontaneous differentiation for 96 h. A fresh medium containing 250 nm retinoic acid (RA) ((MedChemExpress (Monmouth Junction, NJ, USA), HY‐14649)) was then added to the EBs to induce differentiation toward the neurectoderm lineage. EBs were cultured for an additional 7 days, with the medium replaced every 2 days using fresh ESC medium containing 250 nm RA but lacking LIF. After 7 days, the EBs were plated on gelatin‐coated dishes and cultured for an additional 7 days before being collected for further experiments.

### 5.6 Cell Proliferation Assay

The experiment was performed according to the manufacturer's instructions for the CCK‐8 assay (Beyotime, C0037). Briefly, cells were incubated with 10 µL of CCK‐8 reagent per 1000–5000 cells for 1 h, and the absorbance was then measured at 450 nm using a microplate reader (Infinite E Plex).

### 5.7 RNA Extraction and qPCR

Total RNA was extracted using a TRIzol‐based protocol. The extracted RNA was reverse transcribed into cDNA using the HiScript IV All‐in‐One Ultra RT SuperMix for qPCR (Vazyme Biotech Co., Ltd, R433), according to the manufacturer's instructions. qPCR was performed using the Taq Pro Universal SYBR qPCR Master Mix (Vazyme Biotech Co., Ltd, Q712), following the manufacturer's instructions. The qPCR reactions were carried out using the 7500 Real‐Time PCR System (Applied Biosystems).

### 5.8 RNA Dot Blot and ELISA Assay for m^6^A Modification

For RNA dot blot experiment, the extracted RNA was denatured at 95°C for 3 min to disrupt secondary structures, then immediately chilled on ice. RNA samples were loaded onto positively charged nylon membranes (Beyotime, FFN10), and crosslinked using a UV crosslinker. The membrane was washed with TBS containing 0.1% Tween‐20, then blocked with 5% BSA for 1 h at room temperature. The membrane was incubated overnight at 4°C with rabbit anti‐m^6^A antibody (1:1000; CST, #56593). After washing with TBST, the membrane was incubated with the corresponding secondary antibody for 1 h. Blot images were captured using the ChemiDoc Imaging System (BIO‐RAD). Equal amounts of RNA were spotted onto the membrane and stained with methylene blue (Solarbio, G1301) to normalize total RNA levels [66, 67].

The ELISA assay was performed according to the manufacturer's instructions for the EpiQuik m^6^A RNA Methylation Quantification Kit (Colorimetric) (EPIGENTEK, P‐9005). 200 ng RNA from each sample was used for per reaction. Absorbance was measured at 450 nm using a microplate reader (Infinite E Plex).

### 5.9 SELECT

Site‐specific m^6^A detection by SELECT was performed as described previously [48, 49]. For each sample, 5 ng of RNA was combined with 40 nm of each Up and Down probe and 5 µm dNTPs (Beyotime, D7366) in a total volume of 17 µL containing 10 × rCutSmar Buffer (NEB, B6004V). A stepwise annealing program was performed as follows: 90°C for 1 min, 80°C for 1 min, 70°C for 1 min, 60°C for 1 min, 50°C for 1 min, and 40°C for 6 min. Subsequently, 17 µL of the annealed reaction mixture was combined with 3 µL of an enzyme mixture containing 0.01 U of Bst 2.0 DNA polymerase (NEB, 5370S), 0.5 U of SplintR ligase (NEB, M0375S), and 10 nmol of ATP (NEB, P0756S). The final 20 µL reaction mixture was incubated at 40°C for 20 min, denatured at 80°C for 20 min, and held at 4°C. The resulting products were subsequently analyzed by qPCR as described previously. The SELECT product signal at each target site was normalized to the abundance of the corresponding transcript containing that site. Primers and probes used in the SELECT assay are listed in File S2.

### 5.10 Western Blot

Western blot was performed according to our previous protocol [35]. Briefly, proteins with different molecular weights were separated using SDS‐polyacrylamide gel electrophoresis (SDS‐PAGE). Proteins were transferred to polyvinylidene fluoride (PVDF) membranes (Millipore) and blocked either with 2% No Protein Blocking Solution (Sangon Biotech, C530040) for 15 min at room temperature or with 0.1% PBS‐Tween‐20 (PBS‐T) containing 5% milk for 1 h at room temperature. Membranes were incubated overnight at 4°C with the primary antibody, washed with PBS containing 0.1% Tween‐20, and then incubated with the corresponding secondary antibody for 1 h at room temperature. Blot images were captured using the ChemiDoc Imaging System (Bio‐Rad).

### 5.11 Immunofluorescence

Cells were fixed with 4% paraformaldehyde (PFA) for 30 min at room temperature, followed by permeabilization with PBS containing 0.25% Triton X‐100 for 20 min. Cells were blocked with 1% BSA for 30 min, then incubated overnight at 4°C with the primary antibody. After washing with PBST, cells were incubated with the corresponding secondary antibody and Hoechst for 1 h at room temperature. Cells were washed with PBST and then visualized using a Nikon ECLIPSE Ti‐U microscope. Colocalization analysis was performed using ImageJ software.

### 5.12 Co‑Immunoprecipitation

For each sample, experiments were performed according to the guidelines of the PiercClassic Magnetic IP / Co‐IP Kit (Thermo Scientific #88804). Lysed protein samples were incubated overnight at 4°C with either a specific antibody or IgG. Immunoprecipitation was performed using Pierce Protein A/G Magnetic Beads. Proteins were eluted using Lane Marker Sample Buffer diluted fivefold with purified water. Eluted protein samples were mixed with protein loading buffer and heated at 105°C for 10 min before storage. The followed detection was conducted by Western blot.

### 5.13 Cell Cycle Analysis

Propidium iodide (PI) staining was performed to analyze the cell cycle, following a previously reported protocol [68]. Briefly, cells were digested with trypsin, harvested, and fixed in 70% ethanol. Cells were centrifuged and resuspended in 0.25% Triton X‐100, then incubated on ice for 15 min. After centrifugation and removal of the supernatant, cells were resuspended in PBS containing 10 µg/mL RNase A and 20 µg/mL PI (Solarbio, C0080), and incubated at room temperature in the dark for 30 min prior to analysis by flow cytometry (BD Accuri C6 Plus). Cell cycle distribution was analyzed using ModFit LT 5.0 software.

### 5.14 Nascent RNA Synthesis Assay

Nascent RNA synthesis was performed using the BeyoClic EU RNA Synthesis Kit with AF488 (Beyotime, R0301) according to the manufacturer's instructions. Briefly, 1 mm 5‐ethynyl uridine (EU) was added to the medium and incubated for 2 h. Cells were sequentially fixed, washed, and permeabilized using the corresponding reagents. Click reaction solution was added to the cell samples and incubated for 30 min at room temperature in the dark, followed by staining with Hoechst for 10 min. Fluorescence images were captured using a Leica DM4B upright fluorescence microscope.

### 5.15 Isolation of 4sU‐Labeled Newly Transcribed RNA

Reprogramming cells were cultured in medium containing 500 µm 4sU ((MedChemExpress (Monmouth Junction, NJ, USA), HY‐W011793))) for 1 h, after which total RNA was extracted using a TRIzol‐based protocol. Biotinylation and separation of 4sU‐labeled RNA were performed as described previously, with minor modifications [69]. Approximately 100 µg of total RNA was used for each biotinylation reaction. 4sU‐labeled RNA was biotinylated using EZ‐Link HPDP‐Biotin (Thermo Fisher) and further purified using a phenol/chloroform (pH 6.7)‐based protocol. The purified RNA was resuspended in RNase‐free water and incubated with Dynabead Streptavidin Magnetic Beads (Thermo Fisher). Bead‐bound biotinylated RNA was isolated and subsequently eluted with DTT. The eluate was precipitated overnight at −20°C using ethanol and glycogen. Newly transcribed RNA was collected by centrifugation after removal of the supernatant.

### 5.16 Cell Apoptosis

Cell apoptosis was assessed using the Annexin V‐FITC Apoptosis Detection Kit (Beyotime, C1062), according to the manufacturer's guidelines. Annexin V‐FITC, binding buffer, and PI were added to the cell samples and incubated for 10–20 min at room temperature in the dark. Apoptotic cells were observed in situ using a Leica DM4B upright fluorescence microscope.

### 5.17 Karyotype Analysis

0.5 µg/mL colcemid was added to reprogramming cells in the exponential growth phase and incubated for 12 h before harvesting the cells by trypsinization. The cells were resuspended in hypotonic solution (0.075 mol/L KCl) and incubated at 37°C for 30 min. Fresh fixative (methanol: glacial acetic acid = 3:1) was added to the hypotonic solution and incubated for 2 min for pre‐fixation before centrifugation. After discarding the supernatant, the cells were resuspended in cold fresh fixative and incubated on ice for 1 h. The cells were centrifuged again, the supernatant discarded, and then fixed again for 30 min. After a final centrifugation, the cells were resuspended in a small volume of cold fixative and dropped onto clean, chilled slides. After air‐drying, chromosomes were stained with Giemsa solution. Chromosomes were observed under a microscope.

### 5.18 CUT&RUN

CUT&RUN assays were performed using the Hyperactive pA/G‐MNase CUT&RUN Assay Kit for PCR/qPCR (Vazyme, HD101) according to the manufacturer's instructions. Approximately 5*10^^^5 cells were collected for subsequent reactions. Cell samples were incubated overnight at 4°C with specific antibodies or an IgG control, followed by cleavage and DNA release. DNA products obtained from the CUT&RUN assay were quantified by qPCR using primers specific for the relevant genomic regions.

### 5.19 ChIP‐qPCR

ChIP assays were performed using the SimpleChIPEnzymatic Chromatin IP Kit (Magnetic Beads) (CST, #9003s), following the manufacturer's instructions. Briefly, cells were first crosslinked with 1% paraformaldehyde and then quenched with 0.125 m glycine. After nuclear isolation, chromatin was digested with micrococcal nuclease (MNase) and subsequently sonicated to obtain crosslinked chromatin fragments. The chromatin was then incubated overnight at 4°C with specific antibodies or control IgG, followed by incubation with ChIP‐grade Protein G magnetic beads. After elution and reverse crosslinking, DNA was further purified. Finally, the DNA fragments obtained from the ChIP assay were quantified by qPCR using primers specific to the relevant genomic regions.

### 5.20 RNA‐seq and Data Analysis

Total RNA was extracted using TRIzol reagent (Invitrogen), and its quality was assessed using a Bioanalyzer 2100 with the RNA 6000 Nano LabChip Kit (Agilent, 5067‐1511). mRNA was purified from total RNA using Dynabeads Oligo(dT) (Thermo Fisher) and fragmented using the Magnesium RNA Fragmentation Module (NEB, E6150). The cDNA synthesis was carried out using SuperScript II Reverse Transcriptase (Invitrogen, 1896649). Subsequently, the second‐strand synthesis was performed using E. coli DNA polymerase I (NEB, M0209) and RNase H (NEB, M0297), with dUTP (Thermo Fisher, R0133) incorporated into the second strand. Furthermore, the cDNA ends were subjected to end repair, followed by the addition of an adenine (A) base to both termini. The cDNA fragments were then size‐selected and purified using the AMPure XP beads. After digestion of the second strand with UDG (NEB, M0280), PCR amplification was performed to construct the cDNA library. The final libraries were sequenced on an Illumina NovaSeq 6000 platform (LC‐Bio Technology Co., Ltd., Hangzhou, China) according to the manufacturer's protocol.

Raw sequencing data were assessed for quality using FastQC software. Adapter trimming and low‐quality reads filtering were performed using Trimmomatic. High‐quality reads were aligned to the reference genome (mm10 or hg38) using HISAT2 (v2.2.1), followed by conversion and sorting with SAMtools (v1.3.1). Read counts were quantified using FeatureCounts (v2.0.3). The resulting count matrix was imported into the R language (v4.1.0) for downstream bioinformatic analysis. Differential expression analysis was performed using the DESeq2 package. Differentially expressed genes were defined by a threshold of |log_2_FC| ≥ 1.5 and FDR < 0.05.

### 5.21 MeRIP‐seq and Data Analysis

Total RNA was extracted using TRIzol reagent (Invitrogen). RNA concentration and purity were assessed with NanoDrop, and integrity was evaluated using the Bioanalyzer 2100 (Agilent). Poly(A)+ RNA was purified using Dynabeads Oligo(dT)25‐61005 (Thermo Fisher) and fragmented using the Magnesium RNA Fragmentation Module (NEB). Fragmented RNA was immunoprecipitated with an anti‐m^6^A antibody (Synaptic Systems). Immunoprecipitated RNA was reverse transcribed into cDNA using SuperScript II Reverse Transcriptase (Invitrogen, 1896649). Subsequently, the second‐strand synthesis was performed using E. coli DNA polymerase I (NEB, M0209) and RNase H (NEB, M0297), with dUTP (Thermo Fisher, R0133) incorporated into the second strand. Furthermore, the cDNA ends were subjected to end repair, followed by the addition of an adenine (A) base to both termini. The cDNA fragments were then size‐selected and purified using the AMPure XP beads. After digestion of the second strand with UDG (NEB, M0280), PCR amplification was performed to construct the cDNA library. Sequencing was performed on an Illumina NovaSeq 6000 platform (LC‐Bio Technology Co., Ltd., Hangzhou, China) according to the manufacturer's protocol.

FastQC software was used to assess the quality of raw sequencing data. Trimmomatic was then used to trim adapters and filter low‐quality reads. Clean reads were aligned to the mouse reference genome (mm10) using HISAT2 (v2.2.1). SAMtools (v1.3.1) was used to convert and sort alignment files. The R package exomePeak2 was used to perform differential m^6^A peak analysis and characterize m^6^A peak features.

### 5.22 GLORI‐seq

Total RNA was extracted using TRIzol reagent (Invitrogen). The mRNA and small RNA fractions were sequentially enriched and purified from total RNA using the NEBNext Poly(A) mRNA Magnetic Isolation Module (New England Biolabs, USA), followed by the mirVana miRNA Isolation Kit (Thermo Fisher Scientific, USA). After these two purification steps, the resulting RNA sample was enriched mRNA. The enriched mRNA was fragmented in fragmentation buffer (NEB, E6150S) at 94°C for 4 min. Glyoxal solution and DMSO were then added, and the mixture was incubated at 50°C for 30 min in a preheated thermal cycler. After incubation, the samples were cooled to room temperature, and saturated H3BO3 solution (Sigma–Aldrich, B0394) was added. The samples were then incubated at 50°C for an additional 30 min. Subsequently, the samples were transferred to deamination buffer containing 750 mm NaNO2, 40 mm MES (pH 6.0), and 10 µL of glyoxal and incubated for 8 h. The treated mRNA was purified by ethanol precipitation, and sequencing libraries were constructed using the GenSeq Low Input RNA Library Prep Kit (GenSeq, Inc.) according to the manufacturer's instructions. The resulting libraries were sequenced in paired‐end mode on SURFSeq5000.

Following sequencing, raw reads were generated. Q30 scores were calculated, and FastQC and FastQ Screen were used to assess raw‐read quality and potential contamination. Cutadapt (v1.9.3) was then used to remove adapter sequences and low‐quality reads, yielding high‐quality clean reads. Clean reads were aligned to the mm10 reference genome using STAR (v2.7) and Bowtie2 (v2.2.4), and the resulting alignments were stored as BAM files. The GLORI‐tools pipeline (https://github.com/liucongcas/) was used to calculate A‐to‐G conversion rates at single‐nucleotide resolution after multiple filtering steps and to identify m^6^A sites and differentially methylated sites in mRNA. Sites with coverage of ≥ 10 reads and m^6^A ratio of ≥ 0.1 were identified as m^6^A sites.

### 5.23 CUT&Tag

According to our previous protocol [70], approximately 10000 cells were collected for library construction using the Hyperactive Universal CUT&Tag Assay Kit for Illumina Pro (Vazyme Biotech Co.,Ltd, #TD904‐02). The extracted DNA product was amplified by PCR to construct Libraries. PCR cycling began with an initial extension at 72°C for 5 min, followed by denaturation at 98°C for 30 s. This was followed by 14 cycles of denaturation at 98°C for 10 s and annealing at 60°C for 30 s. A final extension was performed at 72°C for 1 min, followed by cooling to 4°C. DNA Clean Beads were used for post‐PCR cleanup. Sequencing was performed on the Illumina NovaSeq 6000 platform (Hangzhou Kaitai Biotechnology Co., Ltd. (Hangzhou, China)) according to the manufacturer's instructions.

### 5.24 ChIP‐seq and CUT&Tag Data Analysis

The acquired raw data were checked for quality using FastQC software, followed by adapter trimming and low‐quality read filtering using Trimmatic. The sequence data were aligned to the mm10 or hg38 genome using Bowtie2 (version 2.3.5.1). The aligned data were then converted and sorted using SAMtools. Peak calling was performed using MACS2 (version 2.1.1), and downstream analysis was carried out with the R package ChIPseeker. DeepTools and IGV were used to analyze enrichment features and visualize the results.

### 5.25 Calculation of TR and TM

The promoter‐proximal region was defined as 30 bp upstream to 300 bp downstream of the TSS, and the gene body was defined as the region spanning from 300 to 3000 bp downstream of the TSS. TR was calculated based on ChIP‐seq or CUT&Tag signals of RNA Pol II or NET‐seq and GRO‐seq data, using the following formula:

$$\begin{array}{ccc} & & \mathrm{Trav}\mathrm{eling}\;\mathrm{Ratio}\;\\ & & \;=\;\frac{\mathrm{RNA}\;\mathrm{Pol}\;\mathrm{II}\;\left(\mathrm{or}\;\mathrm{Nascent}\;\mathrm{RNA}\right)\;\mathrm{Signa}l_{\mathrm{Promoter}\;\mathrm{Proximal}}}{\mathrm{RNA}\;\mathrm{Pol}\;\mathrm{II}\;\left(\mathrm{or}\;\mathrm{Nascent}\;\mathrm{RNA}\right)\mathrm{Signa}l_{\mathrm{gene}\;\mathrm{body}}\;} \end{array}$$

The TR values were log_2_‐transformed for downstream analysis. Based on the log_2_‐transformed TR values, the relevant results were analyzed using R language and GraphPad Prism 5.

For TM analysis, differential enrichment of RNA Pol II signals or nascent RNA transcripts at specific genomic regions was assessed for each gene between the control and treatment groups. After log_2_‐transformation, a two‐dimensional (2D) Gaussian mixture model was fitted using the sklearn mixture package in Python [34]. Gene regions were annotated using the HOMER software.

### 5.26 Statistical Analysis

All statistical analyses were performed using R or GraphPad Prism. For qPCR, ChIP‐qPCR, SELECT, m^6^A ELISA, AP colony quantification, and immunofluorescence image quantification, etc., data are presented as the mean ± standard deviation (SD) from three independent biological replicates. Comparisons between two groups were performed using a two‐tailed Student's t‐test. Significance thresholds were defined as *p* < 0.05 (^*^), 0.01 (^**^), and 0.001 (^***^).

For RNA‐seq analysis, differential gene expression was analyzed using DESeq2. P values were adjusted for multiple testing using the Benjamini–Hochberg method, and genes with |log_2_fold change| ≥ 1.5 and an adjusted *p*‐value (FDR) < 0.05 were defined as differentially expressed genes. For MeRIP‐seq analysis, m^6^A peaks and differential m^6^A peaks were identified using exomePeak2, with an adjusted *p*‐value < 0.05 and an |log_2_fold change| ≥ 1.5. For GLORI‐seq analysis, sites meeting the thresholds in only one group were defined as unique sites, whereas those meeting the thresholds in both groups were defined as intersection sites. Intersection sites with FC > 2 or FC < 0.5 were considered upregulated or downregulated, respectively. For CUT&Tag and ChIP‐seq analysis, peaks were called using MACS2 with a q‐value cutoff of 0.05, and genome‐wide signal profiles were generated from normalized read coverage. For TR and TM analyses, statistical comparisons between groups were performed using the tests indicated in the corresponding figure legends, and *p* < 0.05 was considered statistically significant. GO enrichment analysis was performed using the clusterProfiler R package *p* values were adjusted for multiple testing using the Benjamini–Hochberg method, and GO terms with adjusted *p*‐value (FDR) < 0.05 were considered significantly enriched.

## Author Contributions

Z.Z. contributed to the conception and design of the experiment, performed the experiments and bioinformatic analysis, analyzed and interpreted the data, and wrote the manuscript. J.X., Y.W. (**Yonghong Wu**), and X.H. performed the experiments. Y.Q. and Y.S. performed the bioinformatic analysis. Y.W. (**Yanshuang Wu**) contributed to the conception and design of the experiment. Z.L. provided financial and administrative support. J.W. contributed to the conception and design of the experiment and provided administrative support. L.L. contributed to the conception and design of the experiment, provided financial and administrative support, and wrote the manuscript. All authors read and approved the final manuscript.

## Funding

This work was supported by the National Natural Science Foundation of China (no. 32671086).

## Ethics Statement

All animal experiments were implemented in line with the Guidelines for Animal Experiments of Harbin Medical University (HMUIRB20190011).

## Conflicts of Interest

The authors declare no conflicts of interest.

## Supporting information




**Supporting File**: advs78131‐sup‐0001‐SuppMat.pdf.

## Data Availability

All the CUT&Tag, RNA‐seq, MeRIP‐seq, GLORI‐seq and other next‐generation sequencing data supporting the conclusions presented in this manuscript can be accessed through the NCBI‐GEO (https://www.ncbi.nlm.nih.gov/geo/) or National Genomics Data Center of the China National Center for Bioinformation (https://ngdc.cncb.ac.cn/gsa‐human/). The accession numbers for the related data that were utilized in this study are listed in Supplemental Files 2.
