# Supplementary material for: SEC Mediates m6A Deposition and Transcription Pause Release to Drive Cell Identity Transition
Source: Adv Sci (Weinh). 2026 Oct 6:e78131. Online ahead of print. doi: 10.1002/advs.78131 (PMC13643097; doi:10.1002/advs.78131)
Supplement: Supplementary file 1 [file ADVS-9999-e78131-s001.pdf]

Supplemental file 1: Supplementary figures

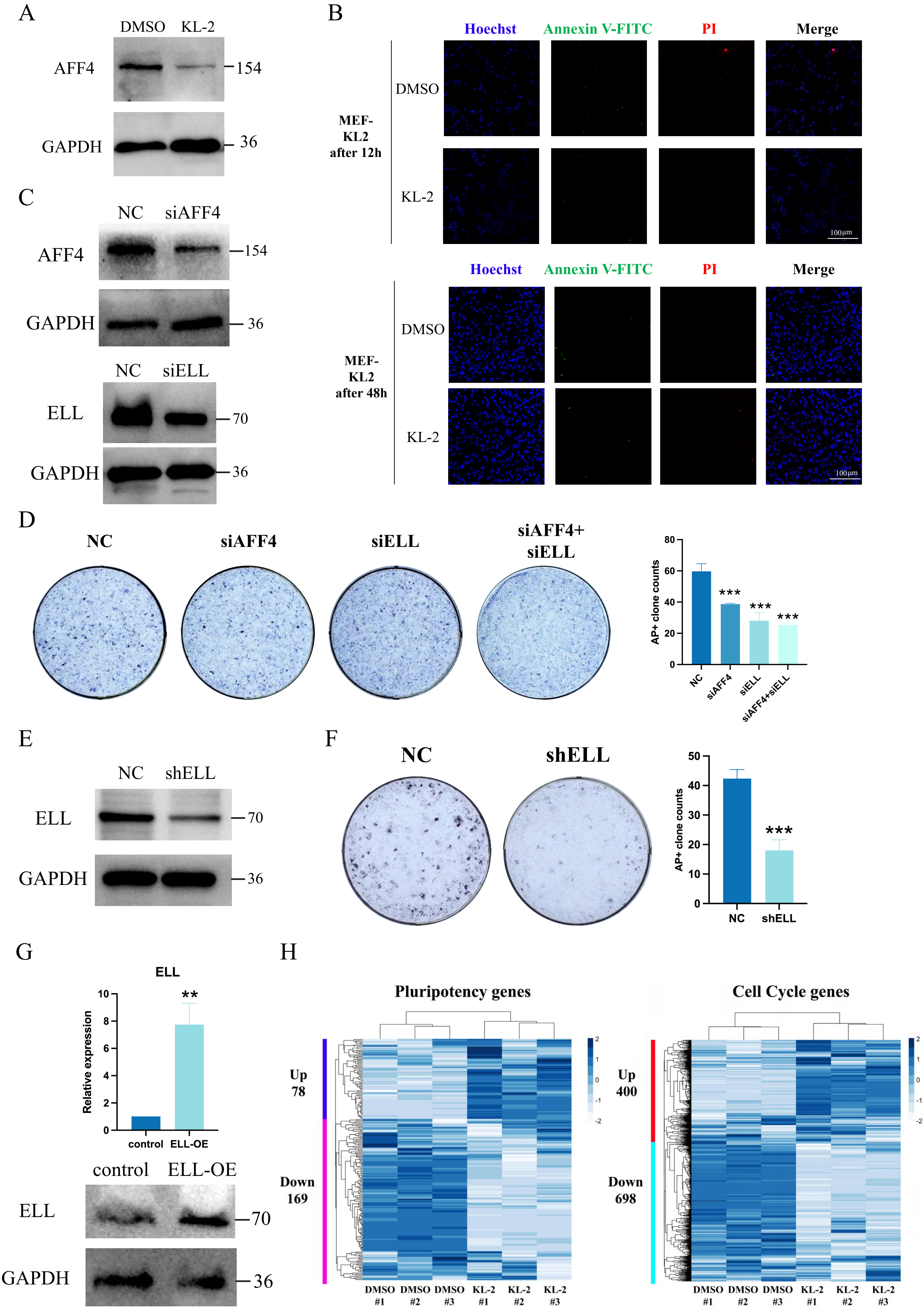

A
DMSO
KL-2
AFF4
154
GAPDH
36
B
Hoechst
Annexin V-FITC
PI
Merge
MEF-
KL2
after 12h
DMSO
KL-2
100μm
Hoechst
Annexin V-FITC
PI
Merge
MEF-
KL2
after 48h
DMSO
KL-2
100μm
C
NC
siAFF4
AFF4
154
GAPDH
36
NC
siELL
ELL
70
GAPDH
36
D
NC
siAFF4
siELL
siAFF4+
siELL
AP+ clone counts
***
NC
siAFF4
siELL
siAFF4+siELL
E
NC
shELL
ELL
70
GAPDH
36
F
NC
shELL
AP+ clone counts
***
NC
shELL
G
ELL
Relative expression
**
control
ELL-OE
control
ELL-OE
ELL
70
GAPDH
36
H
Pluripotency genes
Up
78
Down
169
DMSO #1
DMSO #2
DMSO #3
KL-2 #1
KL-2 #2
KL-2 #3
Cell Cycle genes
Up
400
Down
698
DMSO #1
DMSO #2
DMSO #3
KL-2 #1
KL-2 #2
KL-2 #3

Figure S1: Early inhibition of SEC impairs iPSC reprogramming

A: Western blot analysis showing the expression level of AFF4, a core component of the SEC, in MEFs following KL-2 treatment. The original blots are provided in Supplemental files 3. B: Fluorescence images showing apoptotic cells in MEFs at 12h and 48h after KL-2 treatment. Scale bar = 100 μm. C: Western blot showing knockdown efficiency of siAFF4 and siELL in MEFs. The original blots are provided in Supplemental files 3. D: AP staining on D10 illustrating iPSC colony formation after using siAFF4, siELL, or combined siAFF4+siELL at D0. Quantification of AP^+ colonies are shown in the histogram. E: Western blot showing knockdown efficiency of shELL in MEFs. The original blots are provided in Supplemental files 3. F: AP staining on D10 illustrating iPSC colony formation after using shELL at D0. Quantification of AP^+ colonies are shown in the histogram. G: qPCR (top) and western blot analysis (bottom) showing overexpression efficiency of ELL at the transcript and protein levels, respectively. The original blots are provided in Supplemental files 3. H: Heatmap showing the expression patterns of pluripotency-related (GO:0019827, GO:2000036) and cell cycle–related (GO:0051726) gene sets in DMSO and KL-2 groups. Bars represent the mean ± SD from n = 3 independent experiments. ** $P < 0.01$, *** $P < 0.001$ by two-tailed Student's t-test.

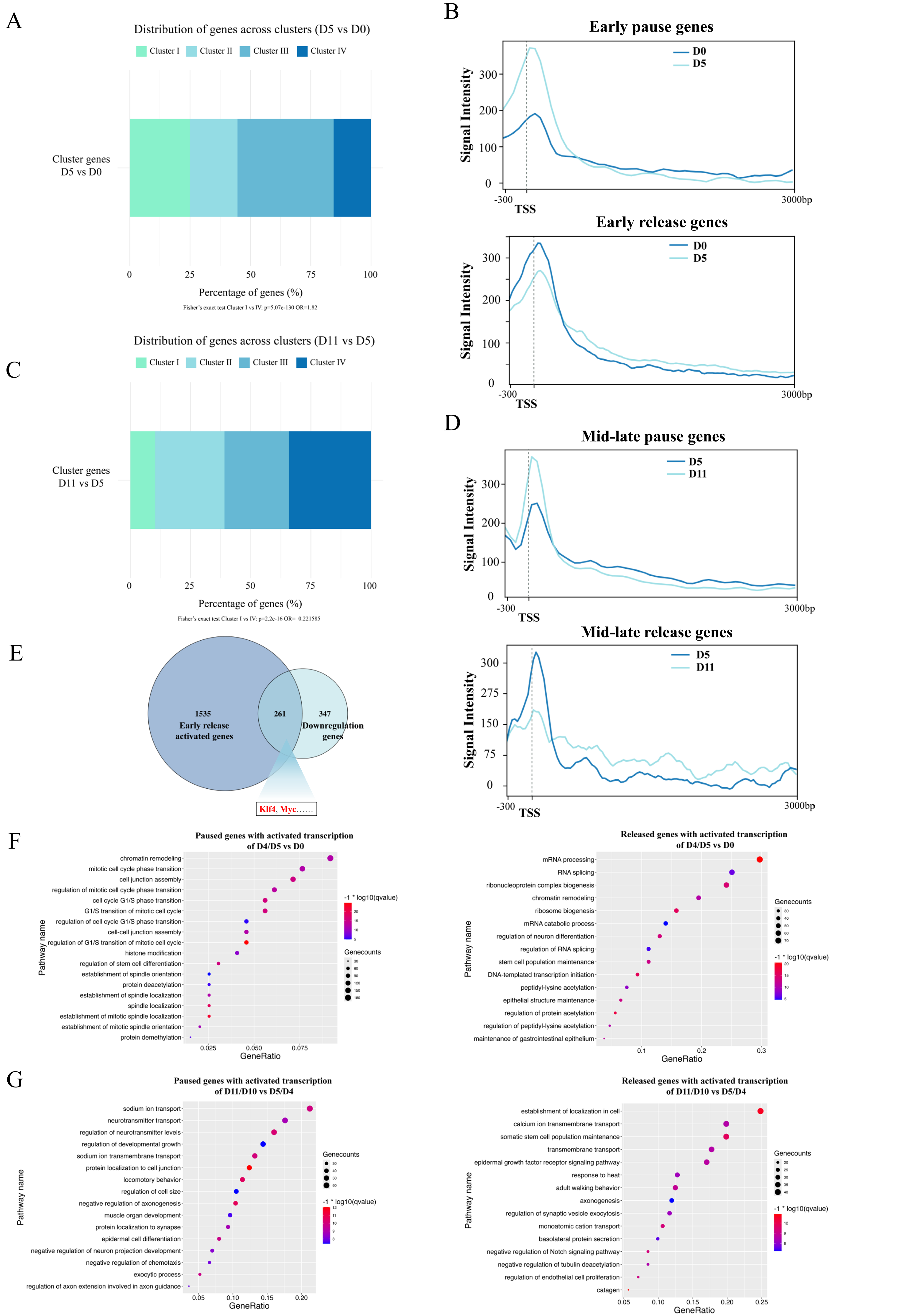

A
Distribution of genes across clusters (D5 vs D0)
Cluster I
Cluster II
Cluster III
Cluster IV
Cluster genes D5 vs D0
0
25
50
75
100
Percentage of genes (%)
Fisher's exact test Cluster I vs IV: p=5.07e-130 OR=1.82
B
Early pause genes
D0
D5
Signal Intensity
300
200
100
0
-300
TSS
3000bp
Early release genes
C
Distribution of genes across clusters (D11 vs D5)
Cluster genes D11 vs D5
Fisher's exact test Cluster I vs IV: p=2.2e-16 OR= 0.221585
D
Mid-late pause genes
D11
Mid-late release genes
275
150
75
E
1535
Early release activated genes
261
347
Downregulation genes
Klf4, Myc......
F
Paused genes with activated transcription of D4/D5 vs D0
chromatin remodeling
mitotic cell cycle phase transition
cell junction assembly
regulation of mitotic cell cycle phase transition
cell cycle G1/S phase transition
G1/S transition of mitotic cell cycle
regulation of cell cycle G1/S phase transition
cell-cell junction assembly
regulation of G1/S transition of mitotic cell cycle
histone modification
regulation of stem cell differentiation
establishment of spindle orientation
protein deacetylation
establishment of spindle localization
spindle localization
establishment of mitotic spindle localization
establishment of mitotic spindle orientation
protein demethylation
Pathway name
GeneRatio
-1 * log10(qvalue)
Genecounts
Released genes with activated transcription of D4/D5 vs D0
mRNA processing
RNA splicing
ribonucleoprotein complex biogenesis
chromatin remodeling
ribosome biogenesis
mRNA catabolic process
regulation of neuron differentiation
regulation of RNA splicing
stem cell population maintenance
DNA-templated transcription initiation
peptidyl-lysine acetylation
epithelial structure maintenance
regulation of protein acetylation
regulation of peptidyl-lysine acetylation
maintenance of gastrointestinal epithelium
G
Paused genes with activated transcription of D11/D10 vs D5/D4
sodium ion transport
neurotransmitter transport
regulation of neurotransmitter levels
regulation of developmental growth
sodium ion transmembrane transport
protein localization to cell junction
locomotory behavior
regulation of cell size
negative regulation of axonogenesis
muscle organ development
protein localization to synapse
epidermal cell differentiation
negative regulation of neuron projection development
negative regulation of chemotaxis
exocytic process
regulation of axon extension involved in axon guidance
Released genes with activated transcription of D11/D10 vs D5/D4
establishment of localization in cell
calcium ion transmembrane transport
somatic stem cell population maintenance
transmembrane transport
epidermal growth factor receptor signaling pathway
response to heat
adult walking behavior
axonogenesis
regulation of synaptic vesicle exocytosis
monoatomic cation transport
basolateral protein secretion
negative regulation of Notch signaling pathway
negative regulation of tubulin deacetylation
regulation of endothelial cell proliferation
catagen

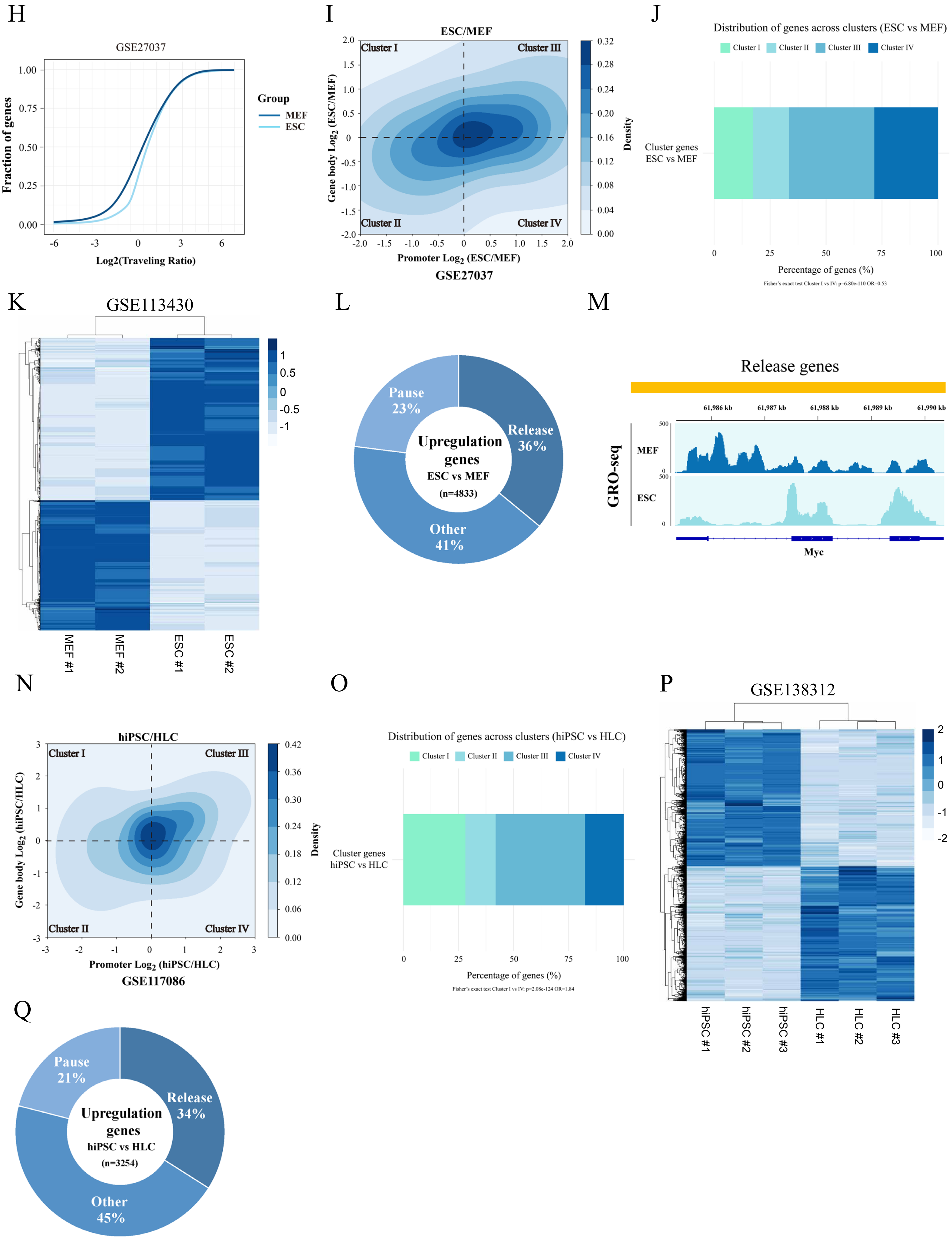

H
GSE27037
Group
MEF
ESC
Fraction of genes
Log2(Traveling Ratio)
I
ESC/MEF
Cluster I
Cluster III
Cluster II
Cluster IV
Gene body Log2 (ESC/MEF)
Promoter Log2 (ESC/MEF)
Density
GSE27037
J
Distribution of genes across clusters (ESC vs MEF)
Cluster I
Cluster II
Cluster III
Cluster IV
Cluster genes
ESC vs MEF
Percentage of genes (%)
Fisher's exact test Cluster I vs IV: p=6.80e-110 OR=0.53
K
GSE113430
MEF #1
MEF #2
ESC #1
ESC #2
L
Pause
23%
Release
36%
Other
41%
Upregulation genes
ESC vs MEF
(n=4833)
M
Release genes
61,986 kb
61,987 kb
61,988 kb
61,989 kb
61,990 kb
GRO-seq
MEF
ESC
Myc
N
hiPSC/HLC
Cluster I
Cluster III
Cluster II
Cluster IV
Gene body Log2 (hiPSC/HLC)
Promoter Log2 (hiPSC/HLC)
Density
GSE117086
O
Distribution of genes across clusters (hiPSC vs HLC)
Cluster I
Cluster II
Cluster III
Cluster IV
Cluster genes
hiPSC vs HLC
Percentage of genes (%)
Fisher's exact test Cluster I vs IV: p=2.08e-124 OR=1.84
P
GSE138312
hiPSC #1
hiPSC #2
hiPSC #3
HLC #1
HLC #2
HLC #3
Q
Pause
21%
Release
34%
Other
45%
Upregulation genes
hiPSC vs HLC
(n=3254)

Figure S2: TR and TM analysis for the transcription pause and release during iPSC reprogramming A: Proportional analysis of genes in each cluster between D5 and D0. Each cluster is represented by the indicated color. Fisher's exact test was conducted to compare Cluster I and Cluster IV. p represents the p-value, and OR indicates the odds ratio. B: RNA Pol II density profiles for early pause genes (Cluster IV from Figure 3D; top) and early release genes (Cluster I from Figure 3D; bottom) comparing D5 versus D0 during reprogramming. C: Proportional analysis of genes in each cluster between D11 and D5. Each cluster is represented by the indicated color. Fisher's exact test was conducted to compare Cluster I and Cluster IV. p represents the p-value, and OR indicates the odds ratio. D: RNA Pol II density profiles for mid-late pause genes (Cluster IV from Figure 3G; top) and mid-late release genes (Cluster I from Figure 3G; bottom) comparing D11 versus D5 during reprogramming. E: Venn diagram showing the overlap between the early transcription released and upregulated genes during reprogramming and the downregulated genes upon D2_KL-2 treatment (Figure 2A). F: GO analysis of upregulated genes with early transcription pause (Cluster IV) and early transcriptional release (Cluster I) in D4/D5 compared to D0. G: GO analysis of upregulated genes with mid-late transcription pause (Cluster IV) and mid-late transcription release (Cluster I) in D11/D10 compared to D5/D4. H: ECDF plot of $\log_2$TR derived from GRO-seq data (GSE27037) in MEFs and ESCs. I: 2D topographic map of TM analysis showing differential nascent RNA at promoter and gene body regions in ESC versus MEF. J: Proportional analysis of genes in each cluster between ESC and MEF. Each cluster is represented by the indicated color. Fisher's exact test was conducted to compare Cluster I and Cluster IV. p represents the p-value, and OR indicates the odds ratio. K: Heatmap displaying differential gene expression between MEF and ESC, based on published RNA-seq data (GSE113430) ($P < 0.05$ and $| \log_2 TR | > 1.5$). L: Pie chart showing the proportions of pause-increased genes, release-increased genes, and other categories between upregulated genes in ESC compared to MEF (n = 4833). M: GRO-seq browser tracks showing nascent RNA at Myc in MEF and ESC, respectively. N: 2D topographic map of TM analysis showing differential nascent RNA at promoter and gene body regions in hiPSC versus HLC (GSE117086). O: Proportional analysis of genes in each cluster between hiPSC versus HLC. Each cluster is represented by the indicated color. Fisher's exact test was conducted to compare Cluster I and Cluster IV. p represents the p-value, and OR indicates the odds ratio. P: Heatmap displaying differential gene expression between hiPSC and HLC, based on published RNA-seq data (GSE138312) ($P < 0.05$ and $| \log_2 TR | > 1.5$). Q: Pie chart showing the proportions of pause-increased genes, release-increased genes, and other categories between upregulated genes in hiPSC compared to HLC (n = 3254).

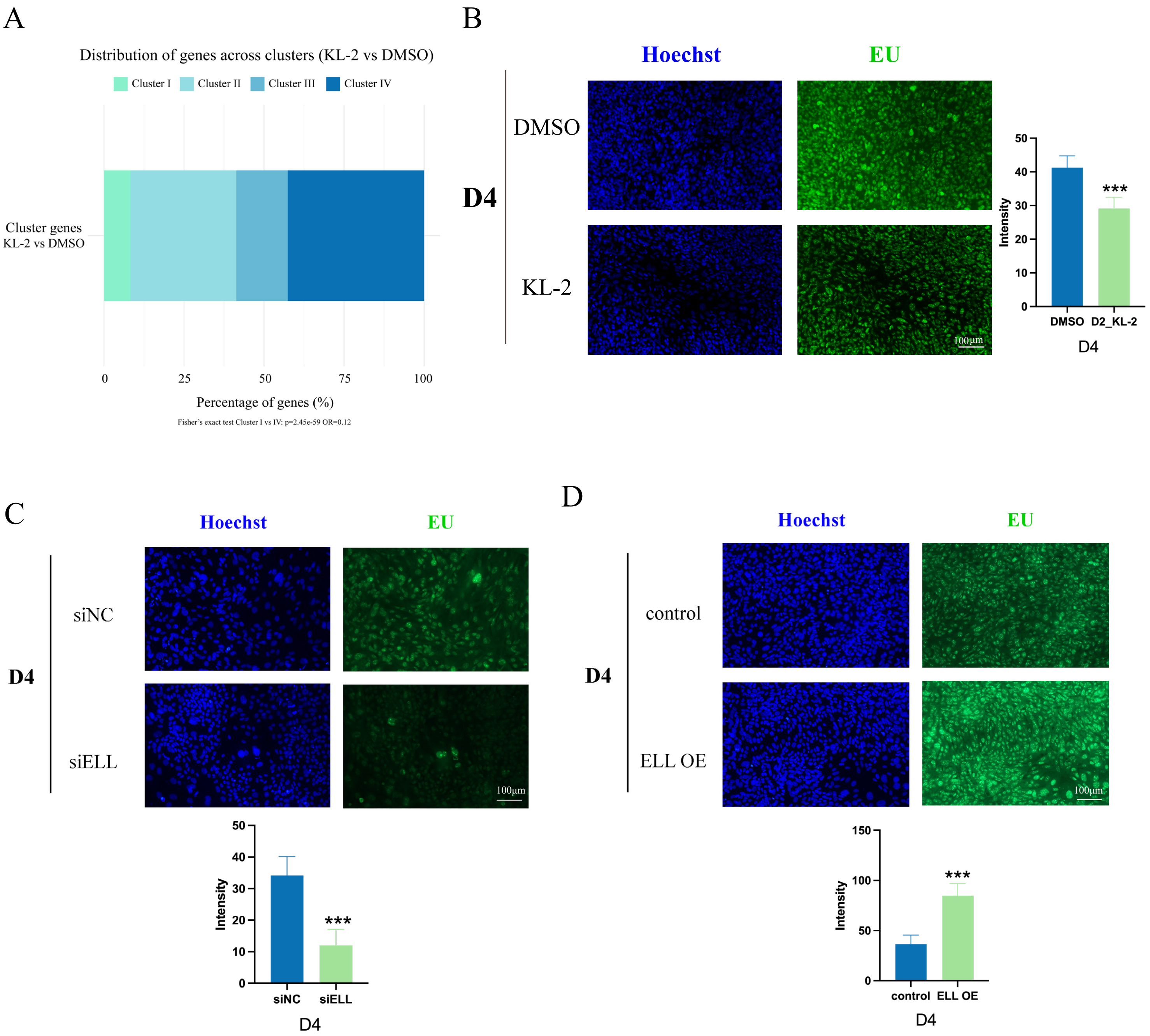

A
Distribution of genes across clusters (KL-2 vs DMSO)
Cluster I
Cluster II
Cluster III
Cluster IV
Cluster genes
KL-2 vs DMSO
0
25
50
75
100
Percentage of genes (%)
Fisher's exact test Cluster I vs IV: p=2.45e-59 OR=0.12
B
Hoechst
EU
DMSO
D4
KL-2
100μm
50
40
30
20
10
0
Intensity
***
DMSO
D2_KL-2
D4
C
Hoechst
EU
siNC
D4
siELL
100μm
50
40
30
20
10
0
Intensity
***
siNC
siELL
D4
D
Hoechst
EU
control
D4
ELL OE
100μm
150
100
50
0
Intensity
***
control
ELL OE
D4

Figure S3: SEC triggers rapid transcription pause release at Klf4 and Myc in early stage

A: Proportional analysis of genes in each cluster between KL-2 and DMSO. Each cluster is represented by the indicated color. Fisher's exact test was conducted to compare Cluster I and Cluster IV. p represents the p-value, and OR indicates the odds ratio. B: Nascent RNA synthesis assay illustrating overall transcriptional activity at D4 under D2_KL-2 treatment. Histogram shows average fluorescence intensity (n > 300 cells). Scale bar=100 μm. C: Nascent RNA synthesis assay illustrating overall transcriptional activity at D4 following siELL interference at day 0 (D0). Histogram shows average fluorescence intensity (n > 300 cells). Scale bar=100 μm. D: Nascent RNA synthesis assay illustrating overall transcriptional activity at D4 following overexpression of ELL. Histogram shows average fluorescence intensity (n > 300 cells). Scale bar=100 μm. Bars represent the mean ± SD from n = 3 independent experiments. *** P < 0.001 by two-tailed Student's t-test.

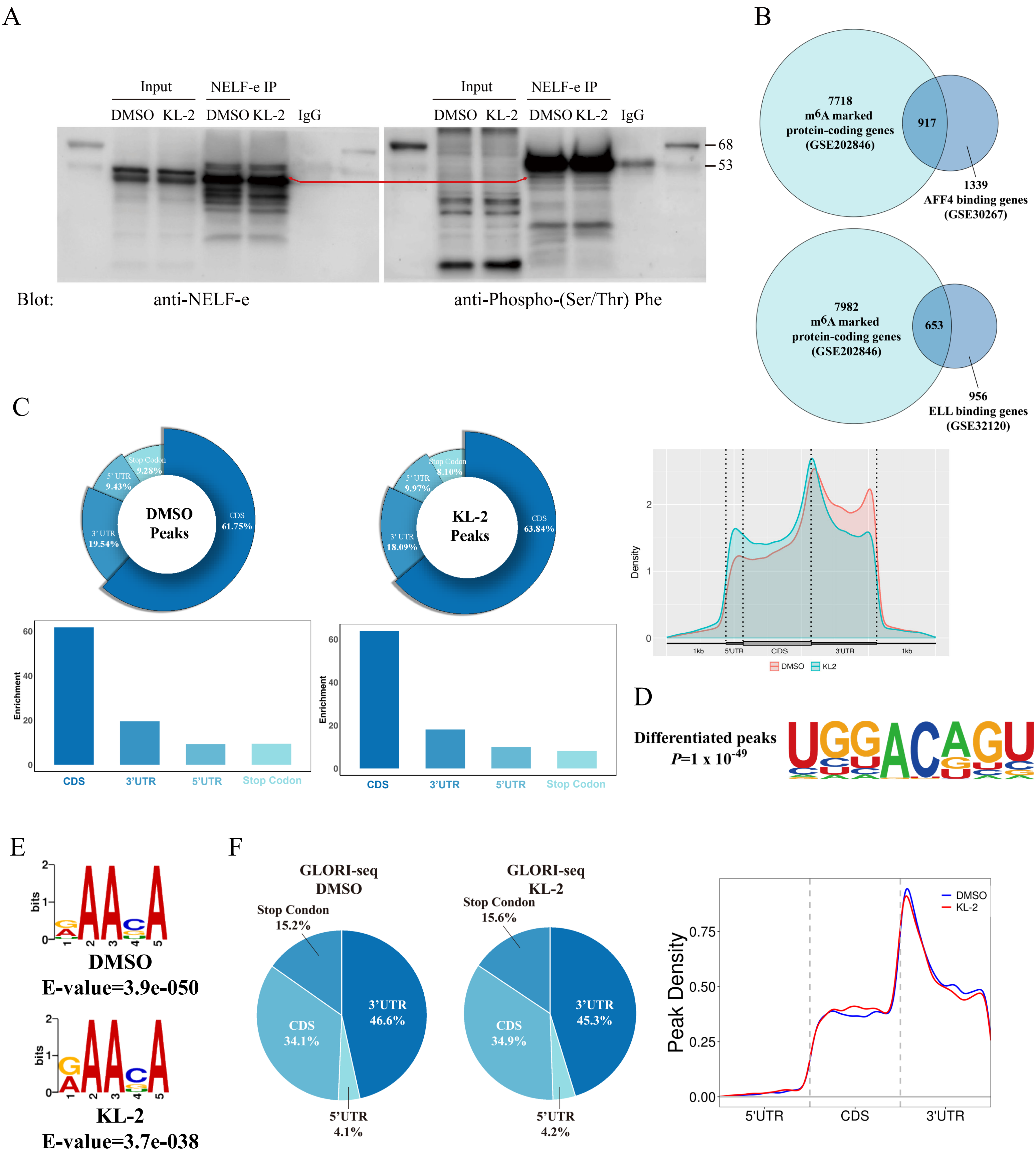
A
Input
NELF-e IP
DMSO KL-2 DMSO KL-2 IgG
Input
NELF-e IP
DMSO KL-2 DMSO KL-2 IgG
68
53
Blot: anti-NELF-e
anti-Phospho-(Ser/Thr) Phe
B
7718 m6A marked protein-coding genes (GSE202846)
917
1339 AFF4 binding genes (GSE30267)
7982 m6A marked protein-coding genes (GSE202846)
653
956 ELL binding genes (GSE32120)
C
DMSO Peaks
CDS 61.75%
3' UTR 19.54%
5' UTR 9.43%
Stop Codon 9.28%
KL-2 Peaks
CDS 63.84%
3' UTR 18.09%
5' UTR 9.97%
Stop Codon 8.10%
Enrichment
CDS
3'UTR
5'UTR
Stop Codon
Density
1kb 5'UTR CDS 3'UTR 1kb
DMSO
KL2
D
Differentiated peaks
P=1 x 10-49
UGGACAGU
E
bits
DMSO
E-value=3.9e-050
KL-2
E-value=3.7e-038
F
GLORI-seq DMSO
Stop Condon 15.2%
3'UTR 46.6%
CDS 34.1%
5'UTR 4.1%
GLORI-seq KL-2
Stop Condon 15.6%
3'UTR 45.3%
CDS 34.9%
5'UTR 4.2%
Peak Density
DMSO
KL-2
5'UTR
CDS
3'UTR

G

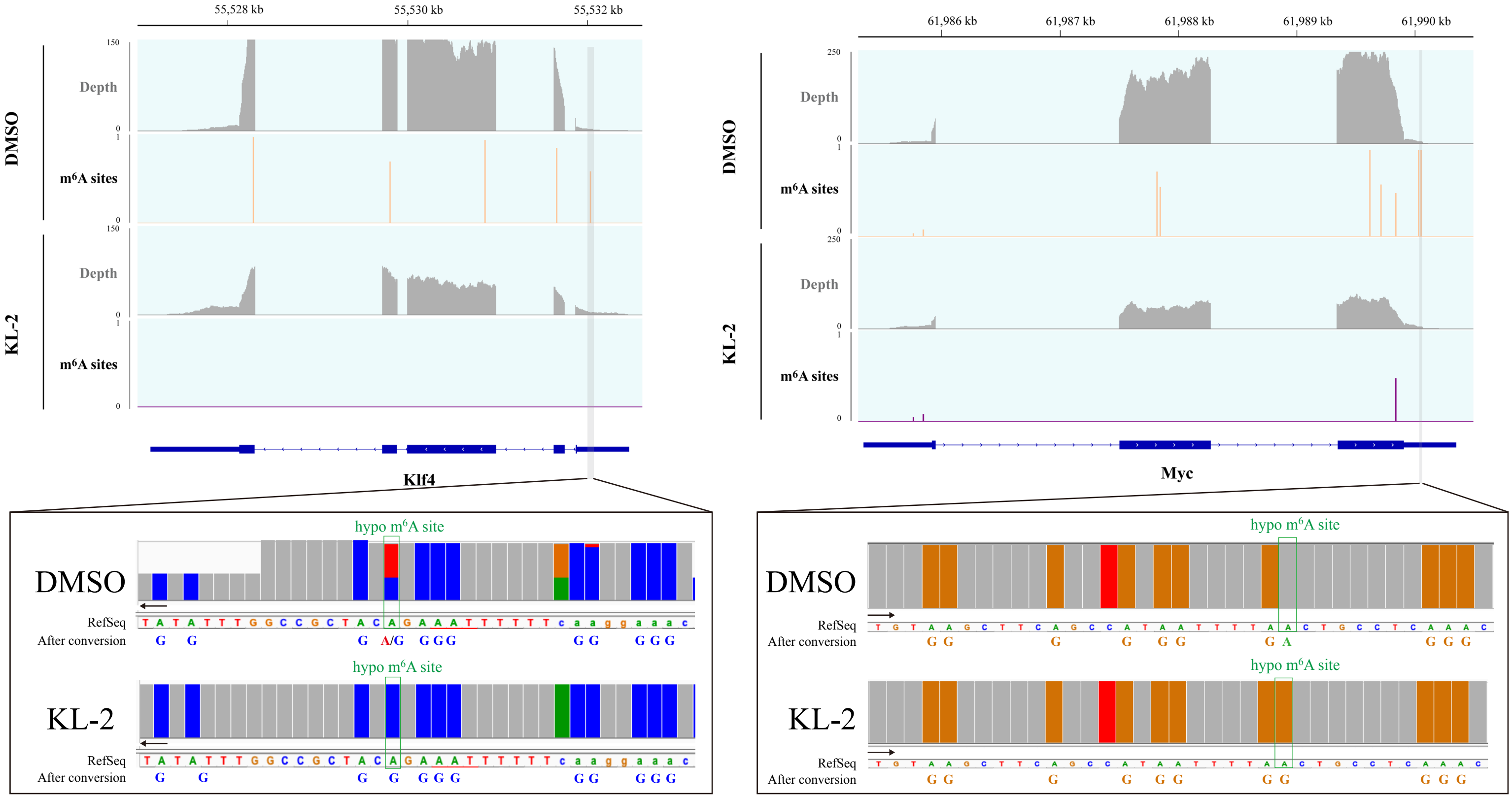

55,528 kb
55,530 kb
55,532 kb
DMSO
KL-2
Depth
m6A sites
Klf4
61,986 kb
61,987 kb
61,988 kb
61,989 kb
61,990 kb
Myc
hypo m6A site
DMSO
KL-2
RefSeq
After conversion

H

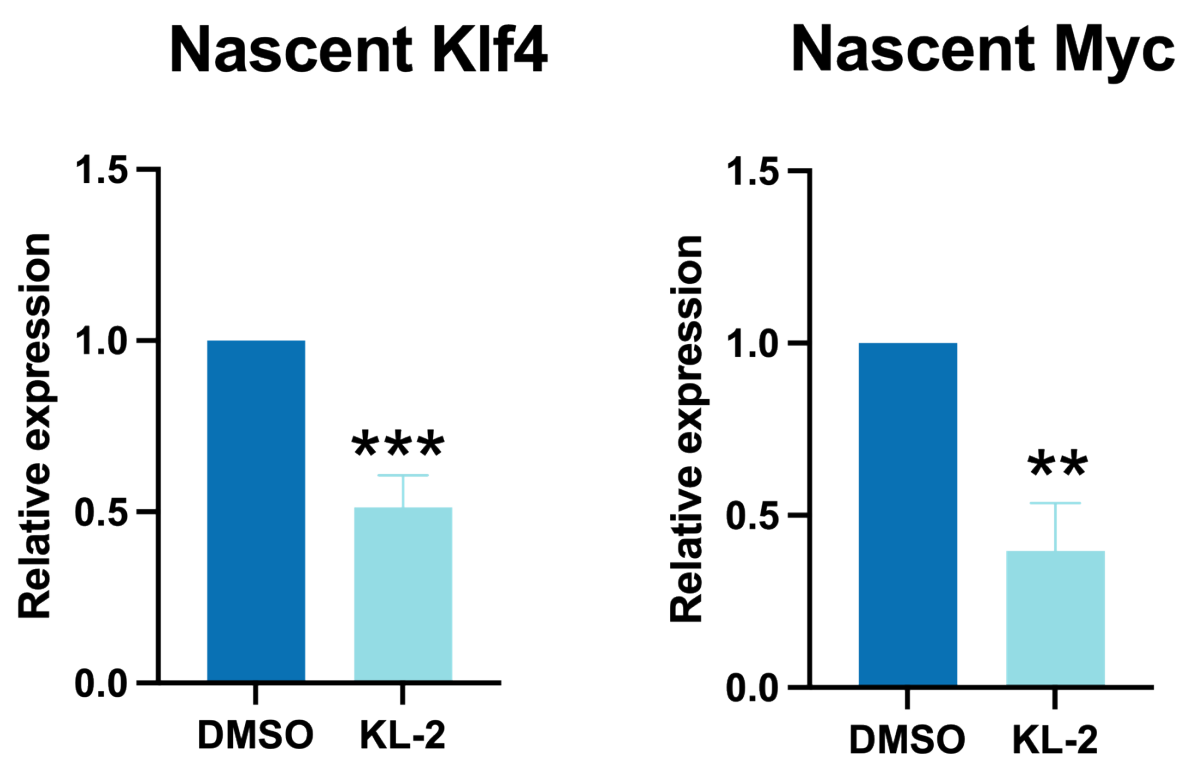

Nascent Klf4
Nascent Myc
Relative expression
1.5
1.0
0.5
0.0
***
**
DMSO
KL-2

Figure S4: SEC regulates m^6A modification during early stage of reprogramming

A: Co-IP was performed to isolate NELF-e–associated proteins. NELF-e–immunoprecipitated proteins from DMSO and KL-2 treated groups were immunoblotted with anti–NELF-e and anti–phospho-(Ser/Thr)-Phe antibodies to assess NELF-e phosphorylation. The red line and arrow indicate the position of NELF-e. The original blots are provided in Supplemental files 3. B: Venn diagrams showing the overlap between m^6A-modified transcripts (MeRIP-seq; GSE202846) and AFF4-bound genes (AFF4 ChIP-seq; GSE30267) (top), and m^6A-modified transcripts and ELL bound genes (ELL ChIP-seq; GSE32120) (bottom). $\log_2$(MeRIP/input) > 1.5 and FDR < 0.05 as significant m^6A-enriched regions. C: (Left) Distribution of m^6A peaks identified by MeRIP-seq at day 4 (D4). Pie charts (top) show the proportions of peaks located in the CDS, 3′ UTR, 5′ UTR, and stop codon regions in the DMSO and D2_KL-2 groups. Bar plots (bottom) display the relative enrichment of m^6A peaks within each region. (Right) Metagene profiles illustrate the genome-wide distribution of m^6A peaks in reprogramming cells (D4) treated with DMSO or D2_KL-2. D: HOMER motif analysis of differentially methylated m^6A peaks following D2_KL-2 treatment. P-value calculated using the HOMER algorithm. E: DREME motif analysis of m^6A sites identified in the DMSO-treated (top) and KL-2-treated (bottom) groups. E-values were calculated using the DREME algorithm. F: Distribution of m^6A sites identified by GLORI-seq on day 4 (D4) of iPSC reprogramming. (Left) Pie charts showing the proportions of m^6A sites located within the CDS, 3′ UTR, 5′ UTR, and stop codon region in the DMSO-treated and KL-2-treated groups. (Right) Metagene profiles showing the transcriptome-wide distribution of m^6A sites in D4 reprogramming cells treated with DMSO or with KL-2 from D2 to D4. G: (Top) GLORI-seq browser tracks illustrating m^6A ratio differences in Klf4 and Myc transcripts between DMSO and D2_KL-2 groups. (Bottom) IGV snapshots showing A-to-G–converted and unconverted adenosines at representative m^6A sites in the Klf4 and Myc transcripts, as highlighted by the gray boxes. Green boxes indicate sites with reduced m^6A modification following SEC inhibition. For Klf4 (negative strand), red and blue denote A and G, respectively, whereas for Myc (positive strand), orange denotes G. In both genes, gray denotes bases matching the reference sequence. H: 4sU-labeled nascent Klf4 and Myc transcript levels in D4 reprogramming cells following early KL-2 treatment initiated. Bars represent the mean ± SD from n = 3 independent experiments. ** $P < 0.01$, *** $P < 0.001$ by two-tailed Student's t-test.

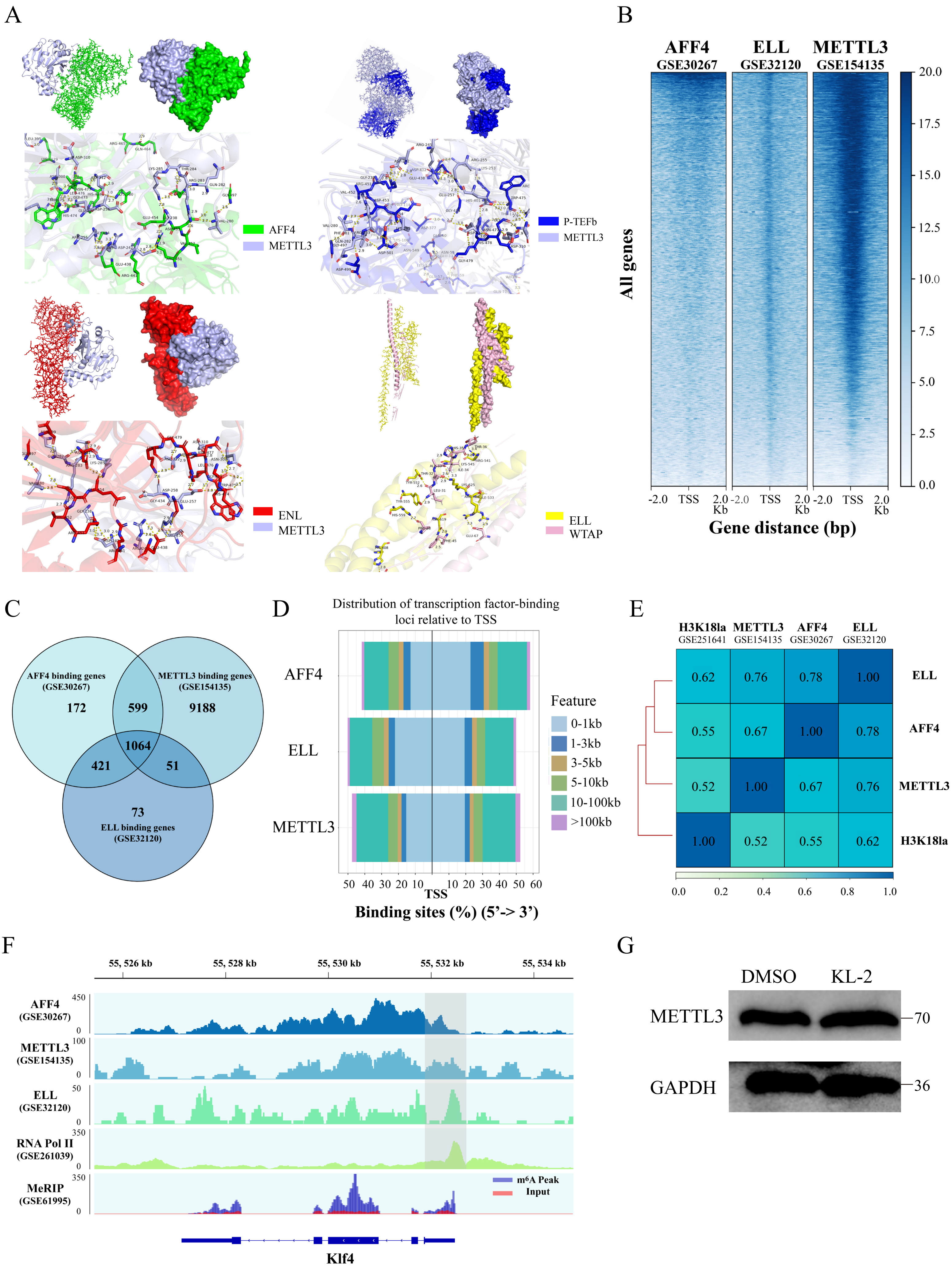
A
AFF4
METTL3
P-TEFb
METTL3
ENL
METTL3
ELL
WTAP
B
AFF4
GSE30267
ELL
GSE32120
METTL3
GSE154135
All genes
20.0
17.5
15.0
12.5
10.0
7.5
5.0
2.5
0.0
-2.0 TSS 2.0 Kb
Gene distance (bp)
C
AFF4 binding genes (GSE30267)
METTL3 binding genes (GSE154135)
ELL binding genes (GSE32120)
172
599
9188
1064
421
51
73
D
Distribution of transcription factor-binding loci relative to TSS
AFF4
ELL
METTL3
Feature
0-1kb
1-3kb
3-5kb
5-10kb
10-100kb
>100kb
50 40 30 20 10 TSS 10 20 30 40 50 60
Binding sites (%) (5'-> 3')
E
H3K18la GSE251641
METTL3 GSE154135
AFF4 GSE30267
ELL GSE32120
ELL 0.62 0.76 0.78 1.00
AFF4 0.55 0.67 1.00 0.78
METTL3 0.52 1.00 0.67 0.76
H3K18la 1.00 0.52 0.55 0.62
0.0 0.2 0.4 0.6 0.8 1.0
F
55, 526 kb
55, 528 kb
55, 530 kb
55, 532 kb
55, 534 kb
AFF4 (GSE30267)
METTL3 (GSE154135)
ELL (GSE32120)
RNA Pol II (GSE261039)
MeRIP (GSE61995)
m6A Peak
Input
Klf4
G
DMSO
KL-2
METTL3
GAPDH
70
36

H

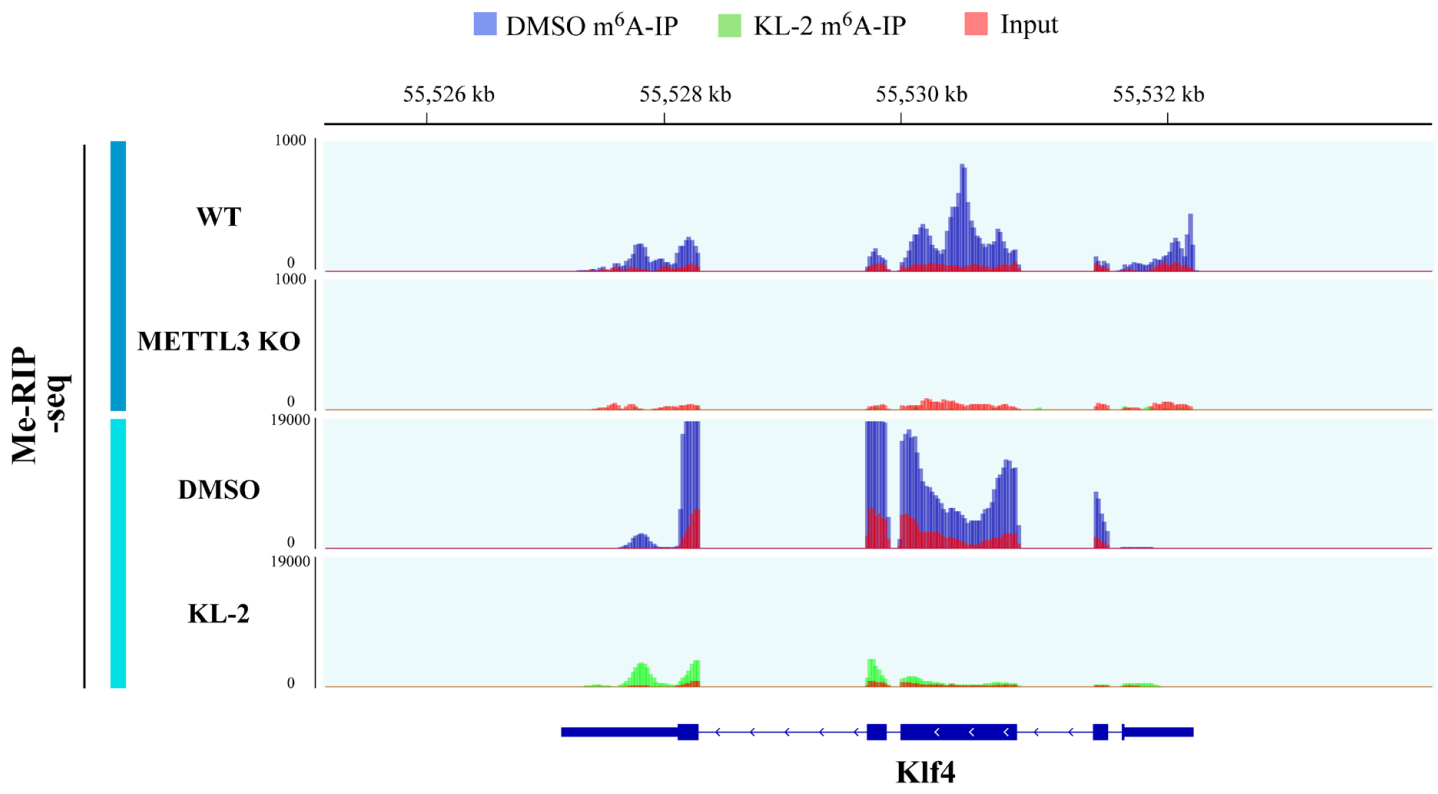
DMSO m6A-IP
KL-2 m6A-IP
Input
55,526 kb
55,528 kb
55,530 kb
55,532 kb
Me-RIP -seq
WT
METTL3 KO
DMSO
KL-2
1000
0
19000
Klf4

Figure S5: SEC facilitates METTL3 recruitment to the Klf4 and Myc loci during reprogramming

A: Molecular docking illustrating potential interaction modes of AFF4-METTL3, P-TEFb-METTL3, ENL-METTL3, ELL-WTAP. Hydrogen bond distances are labeled (below). B: Heatmap showing the genome-wide occupancy of AFF4 (GSE30267), ELL (GSE32120), and METTL3 (GSE154135) centered on transcription start sites (TSS). C: Overlap analysis of target genes bound by AFF4, ELL, and METTL3, respectively. D: Distribution analysis for AFF4, ELL and METTL3-binding loci relative TSS. E: Pearson correlation analysis among ChIP-seq datasets for METTL3, AFF4, and ELL, and an unrelated negative control ChIP sample (H3K18la). F: Genome browser tracks illustrating the co-localization of AFF4, ELL, METTL3, and RNA Pol II ChIP-seq signals, along with MeRIP-seq m^6A signals, at the Klf4 locus. G: Western blot analysis showing METTL3 protein levels in D4 reprogramming cells upon D2_KL-2 treatment. The original blots are provided in Supplemental files 3. H: MeRIP-seq genome browser tracks showing reduced m^6A enrichment across the Klf4 transcript following METTL3 knockdown (GSE61995) and early KL-2 treatment (this study), respectively.

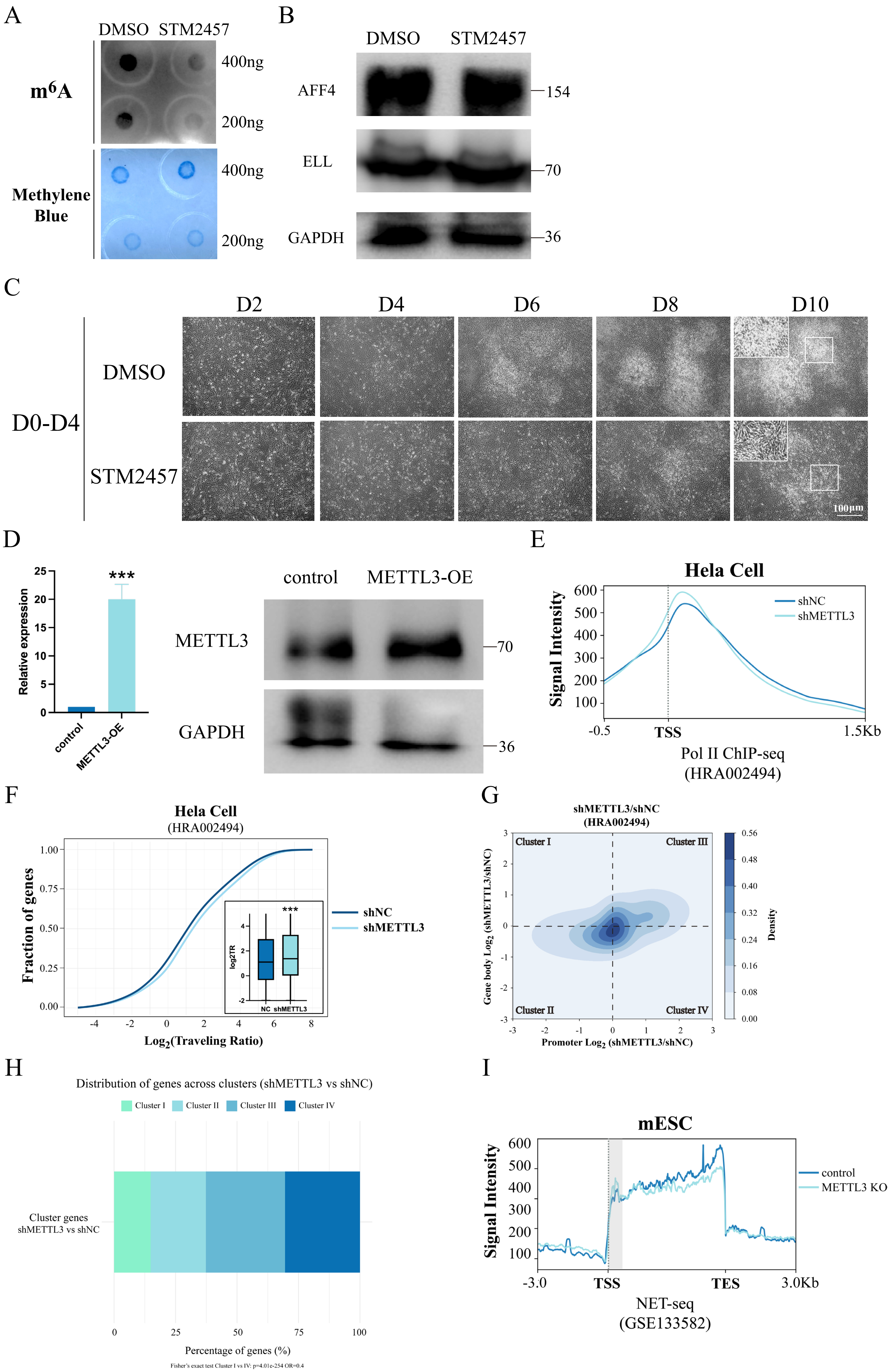

A
DMSO STM2457
m6A
400ng
200ng
Methylene Blue
400ng
200ng
B
DMSO STM2457
AFF4
154
ELL
70
GAPDH
36
C
D2 D4 D6 D8 D10
D0-D4
DMSO
STM2457
100μm
D
Relative expression
***
control
METTL3-OE
control METTL3-OE
METTL3
70
GAPDH
36
E
Hela Cell
Signal Intensity
shNC
shMETTL3
-0.5 TSS 1.5Kb
Pol II ChIP-seq
(HRA002494)
F
Hela Cell
(HRA002494)
Fraction of genes
Log2(Traveling Ratio)
shNC
shMETTL3
log2TR
***
NC shMETTL3
G
shMETTL3/shNC
(HRA002494)
Cluster I
Cluster III
Cluster II
Cluster IV
Gene body Log2 (shMETTL3/shNC)
Promoter Log2 (shMETTL3/shNC)
Density
H
Distribution of genes across clusters (shMETTL3 vs shNC)
Cluster I Cluster II Cluster III Cluster IV
Cluster genes
shMETTL3 vs shNC
Percentage of genes (%)
Fisher's exact test Cluster I vs IV: p=4.01e-254 OR=0.4
I
mESC
Signal Intensity
control
METTL3 KO
-3.0 TSS TES 3.0Kb
NET-seq
(GSE133582)

J

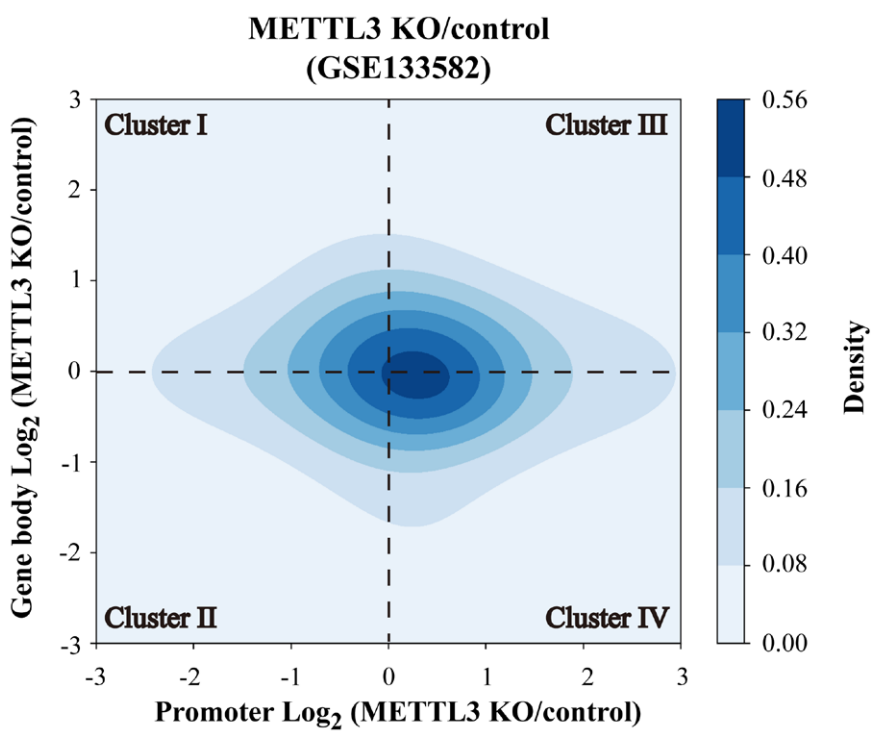

METTL3 KO/control
(GSE133582)
Cluster I
Cluster III
Cluster II
Cluster IV
Gene body Log2 (METTL3 KO/control)
Promoter Log2 (METTL3 KO/control)
Density
3
2
1
0
-1
-2
-3
0.56
0.48
0.40
0.32
0.24
0.16
0.08
0.00

K

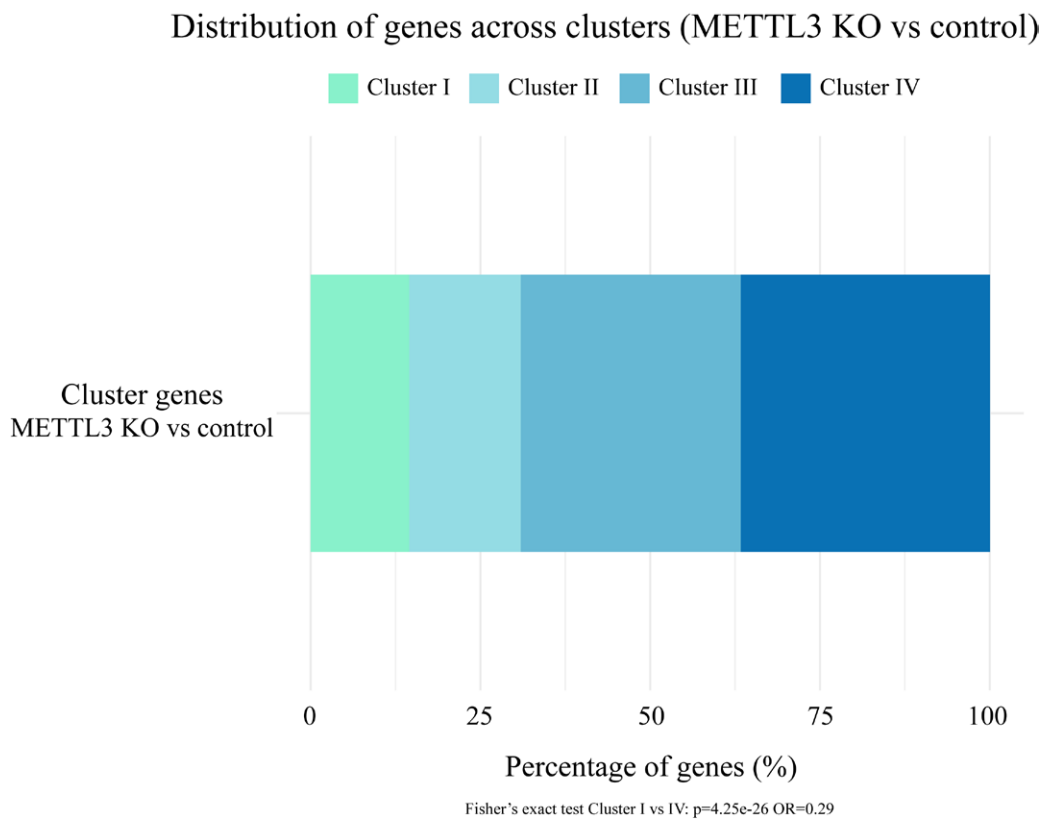

Distribution of genes across clusters (METTL3 KO vs control)
Cluster I
Cluster II
Cluster III
Cluster IV
Cluster genes
METTL3 KO vs control
0
25
50
75
100
Percentage of genes (%)
Fisher's exact test Cluster I vs IV: p=4.25e-26 OR=0.29

L

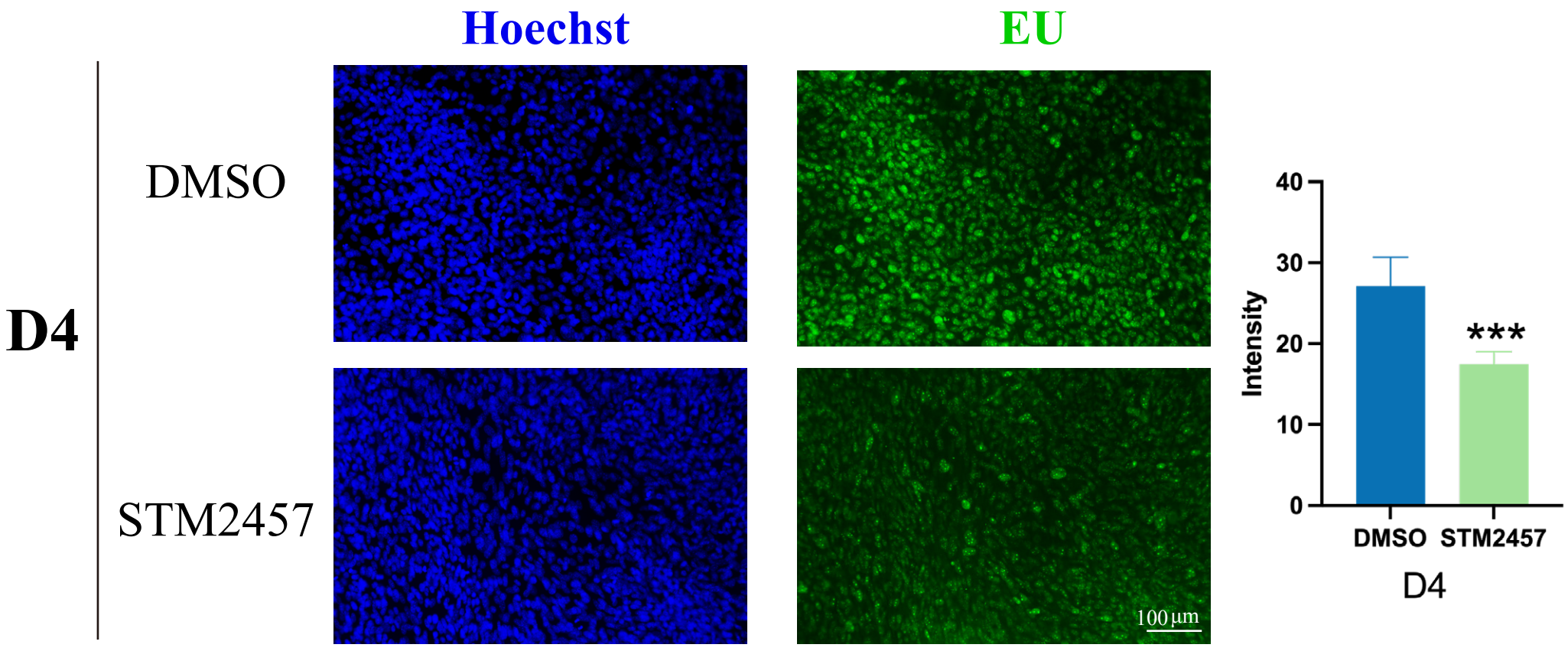

Hoechst
EU
D4
DMSO
STM2457
100 μm
Intensity
40
30
20
10
0
***
DMSO
STM2457
D4

Figure S6: SEC cooperates with METTL3 to promote transcription pause release and activates Klf4 and Myc expression

A: RNA dot blot analysis showing global m^6A levels of total RNA in MEFs treated with STM2457. Methylene blue staining was used as a loading control for RNA input. The original blot is provided in Supplemental files 3. B: Western blot analysis of AFF4 and ELL protein levels in D4 reprogramming cells following early STM2457 treatment (D0–D4). The original blots are provided in Supplemental files 3. C: Phase contrast images showing cell morphology from D2 to D10 of reprogramming under early STM2457 treatment. Scale bar = 100 μm. D: qPCR (left) and western blot (right) analyses showing the transcription and protein overexpression efficiency of METTL3 following METTL3 OE. The original blots are provided in Supplemental files 3. E-H: Analysis based on published ChIP-seq data of RNA Pol II in HeLa cells (HRA002494). Metagene profiles showing average RNA Pol II occupancy in shNC and shMETTL3 groups (E). ECDF and box plots of log_2-transformed TR values comparing shNC and shMETTL3 groups (F). 2D topographic map from TM analysis illustrating differential RNA Pol II enrichment at promoter and gene body regions between shMETTL3 and shNC (G). Proportional analysis of genes in each cluster between shMETTL3 and shNC. Each cluster is represented by the indicated color. Fisher's exact test was conducted to compare Cluster I and Cluster IV. p represents the p-value, and OR indicates the odds ratio (H). I-K: Analysis based on published NET-seq data in mESCs (GSE133582). Metagene profiles of average nascent transcripts occupancies in control and METTL3 KO groups (I). 2D topographic map from TM analysis displaying differential enrichment of nascent transcripts at promoter and gene body regions between METTL3 KO and control groups (J). Proportional analysis of genes in each cluster between METTL3 KO and control. Each cluster is represented by the indicated color. Fisher's exact test was conducted to compare Cluster I and Cluster IV. p represents the p-value, and OR indicates the odds ratio (K). L: Nascent RNA synthesis assay showing overall transcriptional activity at D4 under early STM2457 treatment. Histogram depicts average fluorescence intensity ($n > 300$ cells). Scale bar= 100 μm. Bars represent the mean ± SD from $n = 3$ independent experiments. *** $P < 0.001$ by two-tailed Student's t-test.

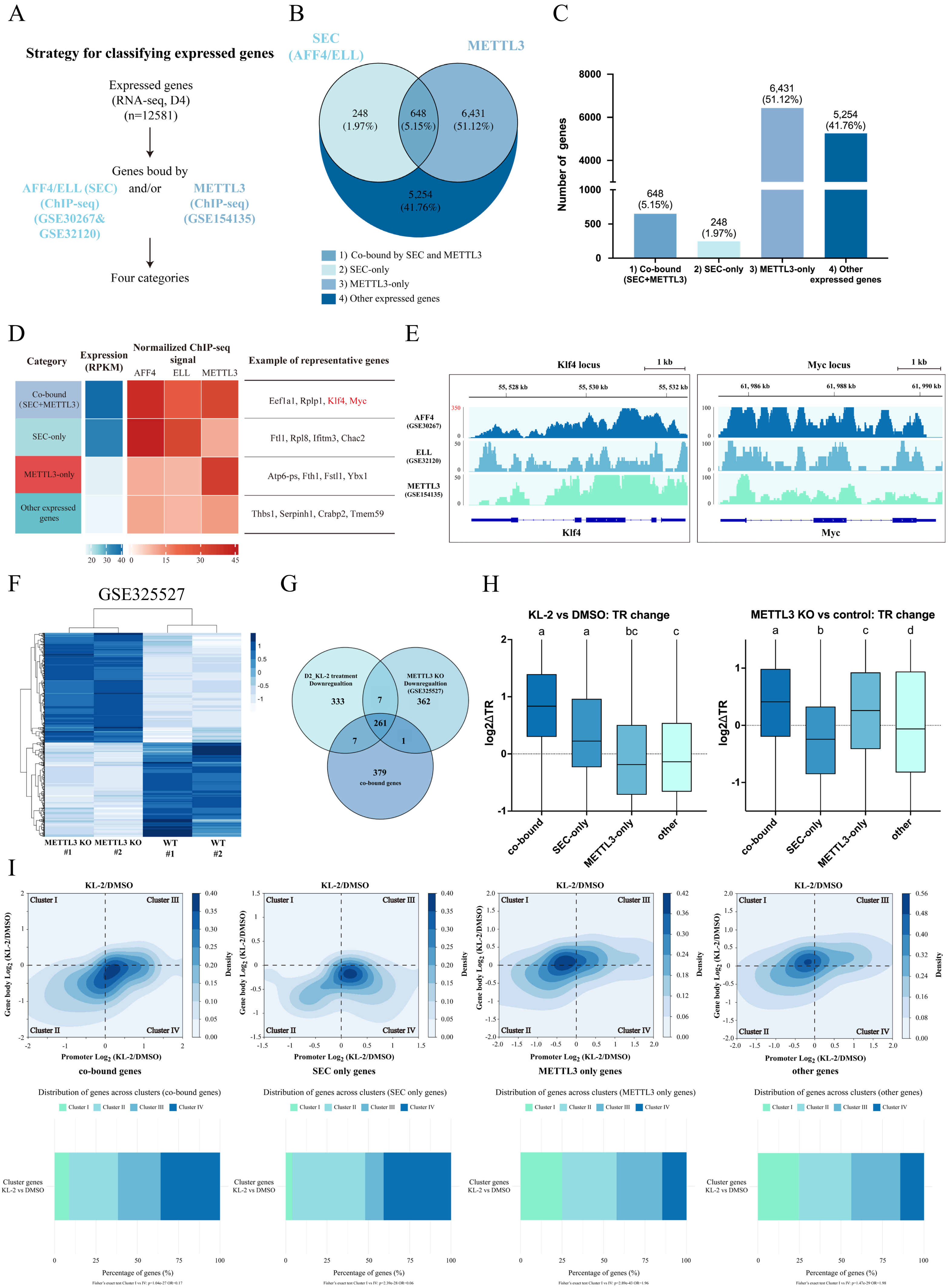
A
Strategy for classifying expressed genes
Expressed genes
(RNA-seq, D4)
(n=12581)
Genes boud by
AFF4/ELL (SEC)
(ChIP-seq)
(GSE30267&
GSE32120)
and/or
METTL3
(ChIP-seq)
(GSE154135)
Four categories
B
SEC
(AFF4/ELL)
METTL3
248
(1.97%)
648
(5.15%)
6,431
(51.12%)
5.254
(41.76%)
1) Co-bound by SEC and METTL3
2) SEC-only
3) METTL3-only
4) Other expressed genes
C
Number of genes
6,431
(51.12%)
5,254
(41.76%)
648
(5.15%)
248
(1.97%)
1) Co-bound
(SEC+METTL3)
2) SEC-only
3) METTL3-only
4) Other
expressed genes
D
Category
Expression
(RPKM)
Normalized ChIP-seq
signal
AFF4
ELL
METTL3
Example of representative genes
Co-bound
(SEC+METTL3)
SEC-only
METTL3-only
Other expressed
genes
Eef1a1, Rplp1, Klf4, Myc
Ftl1, Rpl8, Ifitm3, Chac2
Atp6-ps, Fth1, Fstl1, Ybx1
Thbs1, Serpinh1, Crabp2, Tmem59
E
Klf4 locus
1 kb
55, 528 kb
55, 530 kb
55, 532 kb
AFF4
(GSE30267)
ELL
(GSE32120)
METTL3
(GSE154135)
Klf4
Myc locus
61, 986 kb
61, 988 kb
61, 990 kb
Myc
F
GSE325527
METTL3 KO
#1
METTL3 KO
#2
WT
#1
WT
#2
G
D2_KL-2 treatment
Downregualtion
333
METTL3 KO
Downregualtion
(GSE325527)
362
7
261
7
1
379
co-bound genes
H
KL-2 vs DMSO: TR change
log2ΔTR
a
a
bc
c
co-bound
SEC-only
METTL3-only
other
METTL3 KO vs control: TR change
a
b
c
d
I
KL-2/DMSO
Cluster I
Cluster II
Cluster III
Cluster IV
Gene body Log2 (KL-2/DMSO)
Promoter Log2 (KL-2/DMSO)
Density
co-bound genes
SEC only genes
METTL3 only genes
other genes
Distribution of genes across clusters (co-bound genes)
Distribution of genes across clusters (SEC only genes)
Distribution of genes across clusters (METTL3 only genes)
Distribution of genes across clusters (other genes)
Cluster genes
KL-2 vs DMSO
Percentage of genes (%)

J

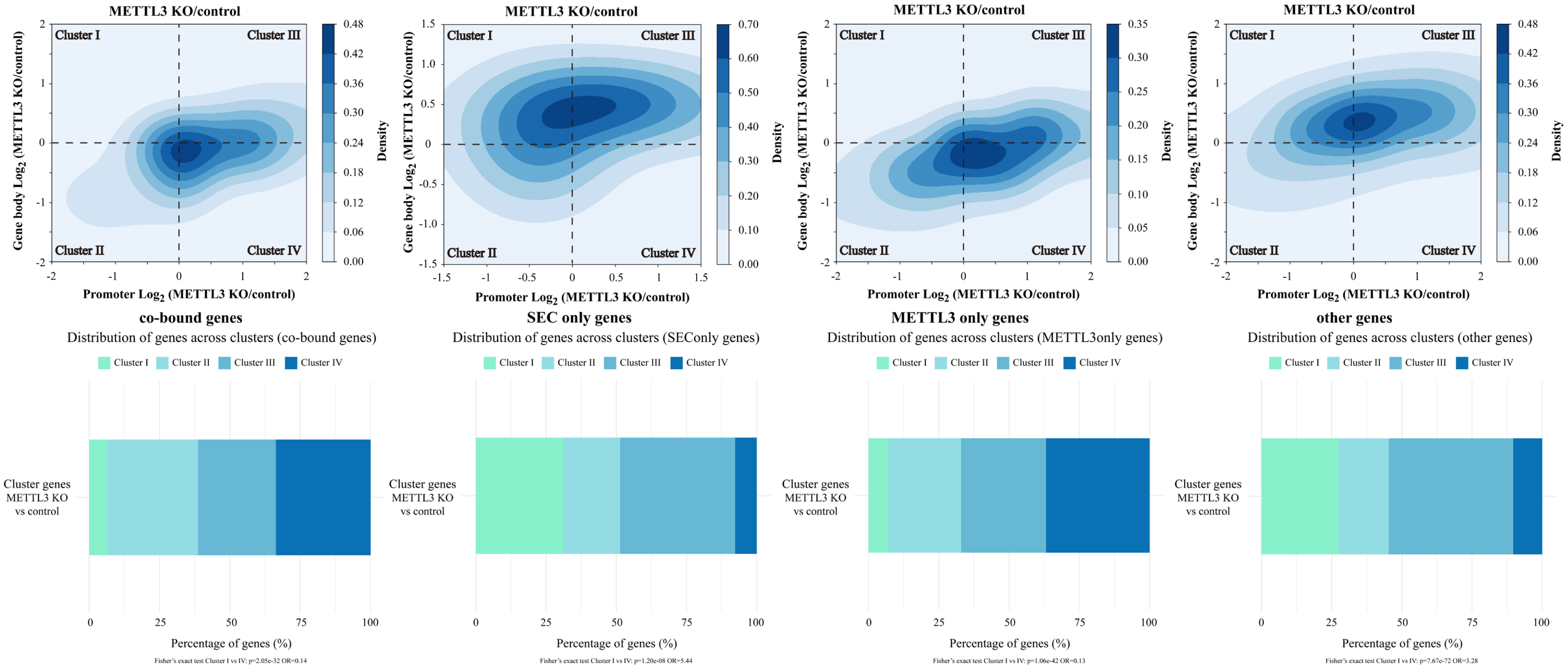

METTL3 KO/control
Cluster I
Cluster II
Cluster III
Cluster IV
Gene body Log2 (METTL3 KO/control)
Promoter Log2 (METTL3 KO/control)
Density
co-bound genes
Distribution of genes across clusters (co-bound genes)
SEC only genes
Distribution of genes across clusters (SEConly genes)
METTL3 only genes
Distribution of genes across clusters (METTL3only genes)
other genes
Distribution of genes across clusters (other genes)
Cluster genes METTL3 KO vs control
Percentage of genes (%)
Fisher's exact test Cluster I vs IV: p=2.05e-32 OR=0.14
Fisher's exact test Cluster I vs IV: p=1.20e-08 OR=5.44
Fisher's exact test Cluster I vs IV: p=1.06e-42 OR=0.13
Fisher's exact test Cluster I vs IV: p=7.67e-72 OR=3.28

Figure S7: SEC and METTL3 may jointly facilitate transcription pause release

A: Schematic strategy for classifying genes expressed at D4 (RPKM>1) into categories based on co-occupancy by SEC and METTL3 or occupancy by either factor alone, using ChIP-seq datasets for AFF4 (GSE30267), ELL (GSE32120), and METTL3 (GSE154135). B: Venn diagram showing the overlap between genes bound by SEC and those bound by METTL3. C: Bar plot showing the number of D4-expressed genes in each category. D: Expression heatmap showing the RPKM values of genes in each category at D4 (left); heatmap of normalized ChIP-seq signals showing AFF4, ELL, and METTL3 occupancy across genes in each category (middle); and table listing representative genes from each category (right). E: ChIP-seq genome browser tracks showing AFF4, ELL, and METTL3 occupancy at the Klf4 and Myc loci. F: Heatmap showing differentially expressed genes between WT and METTL3-knockout cells, based on published RNA-seq data (GSE325527) ($P < 0.05$ and $|log2FC| > 1.5$). G: Overlap analysis of genes downregulated following D2 KL-2 treatment, genes downregulated following METTL3 knockout, and expressed genes co-occupied by SEC and METTL3. H: Box plots showing log_2-transformed changes in the TR across the indicated gene groups in KL-2 vs DMSO (left) and METTL3 KO versus WT (right), based on RNA Pol II CUT&Tag and published NET-seq data (GSE133582), respectively. P values were calculated using Student's t-test. Groups labeled with the same letter are not significantly different, whereas groups labeled with different letters are significantly different. I: 2D topographic map of TM analysis showing changes in RNA Pol II occupancy across promoter and gene-body regions for each gene group in KL-2 versus DMSO cells (top). The proportions of genes assigned to each cluster within each gene group are shown at the (bottom). Each cluster is represented by the indicated color. Fisher's exact test was conducted to compare Cluster I and Cluster IV. p represents the p-value, and OR indicates the odds ratio. J: 2D topographic map of TM analysis showing changes in nascent RNA across promoter and gene-body regions for each gene group in METTL3 KO versus control cells (top). The proportions of genes assigned to each cluster within each gene group are shown at the (bottom). Each cluster is represented by the indicated color. Fisher's exact test was conducted to compare Cluster I and Cluster IV. p represents the p-value, and OR indicates the odds ratio.

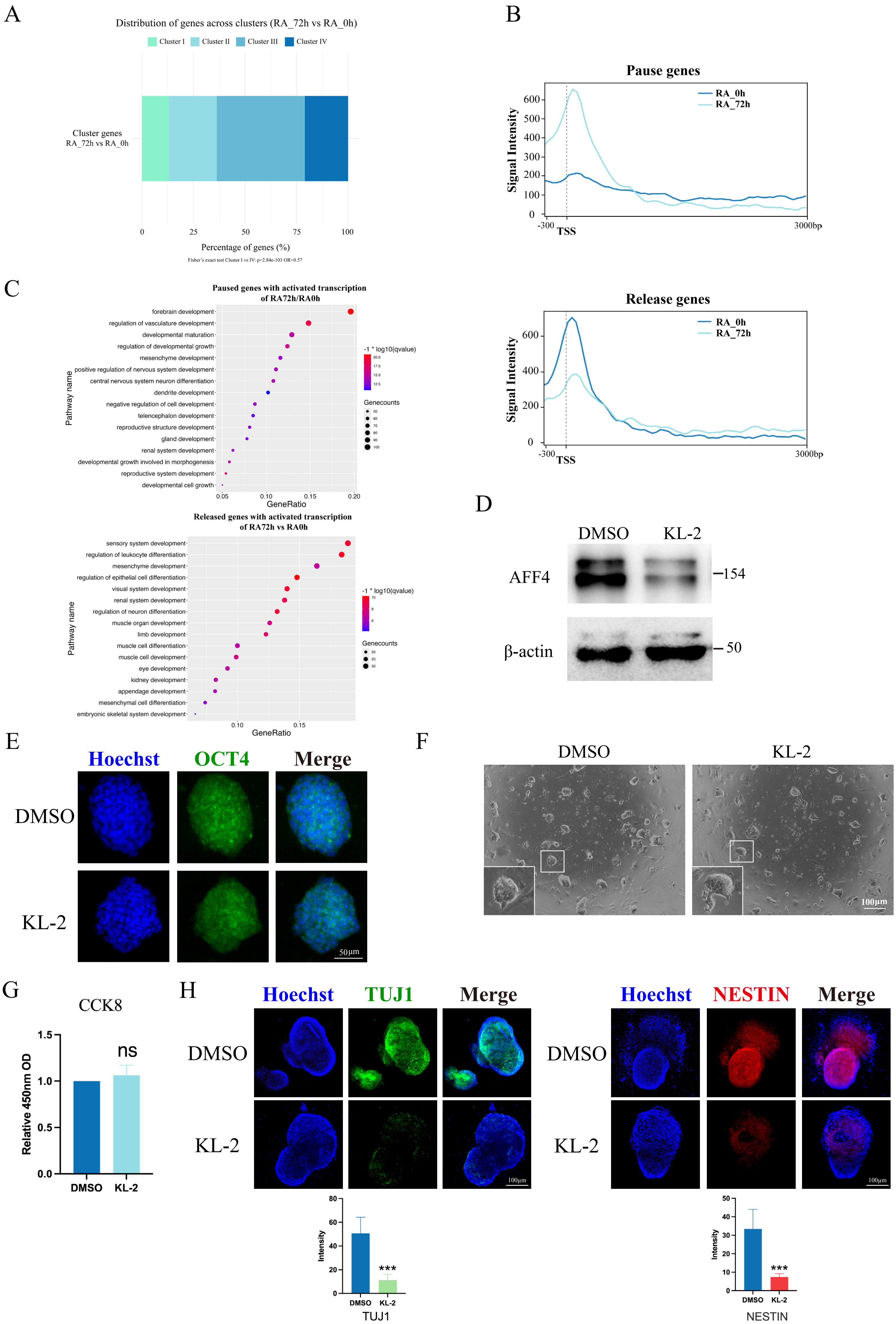
A
Distribution of genes across clusters (RA_72h vs RA_0h)
Cluster I
Cluster II
Cluster III
Cluster IV
Cluster genes RA_72h vs RA_0h
Percentage of genes (%)
B
Pause genes
Release genes
Signal Intensity
RA_0h
RA_72h
TSS
-300
3000bp
C
Paused genes with activated transcription of RA72h/RA0h
forebrain development
regulation of vasculature development
developmental maturation
regulation of developmental growth
mesenchyme development
positive regulation of nervous system development
central nervous system neuron differentiation
dendrite development
negative regulation of cell development
telencephalon development
reproductive structure development
gland development
renal system development
developmental growth involved in morphogenesis
reproductive system development
developmental cell growth
Pathway name
GeneRatio
-1 * log10(qvalue)
Genecounts
Released genes with activated transcription of RA72h vs RA0h
sensory system development
regulation of leukocyte differentiation
mesenchyme development
regulation of epithelial cell differentiation
visual system development
renal system development
regulation of neuron differentiation
muscle organ development
limb development
muscle cell differentiation
muscle cell development
eye development
kidney development
appendage development
mesenchymal cell differentiation
embryonic skeletal system development
D
DMSO
KL-2
AFF4
β-actin
154
50
E
Hoechst
OCT4
Merge
DMSO
KL-2
50μm
F
DMSO
KL-2
100μm
G
CCK8
ns
Relative 450nm OD
DMSO
KL-2
H
Hoechst
TUJ1
Merge
NESTIN
DMSO
KL-2
100μm
Intensity
***
TUJ1
NESTIN

I

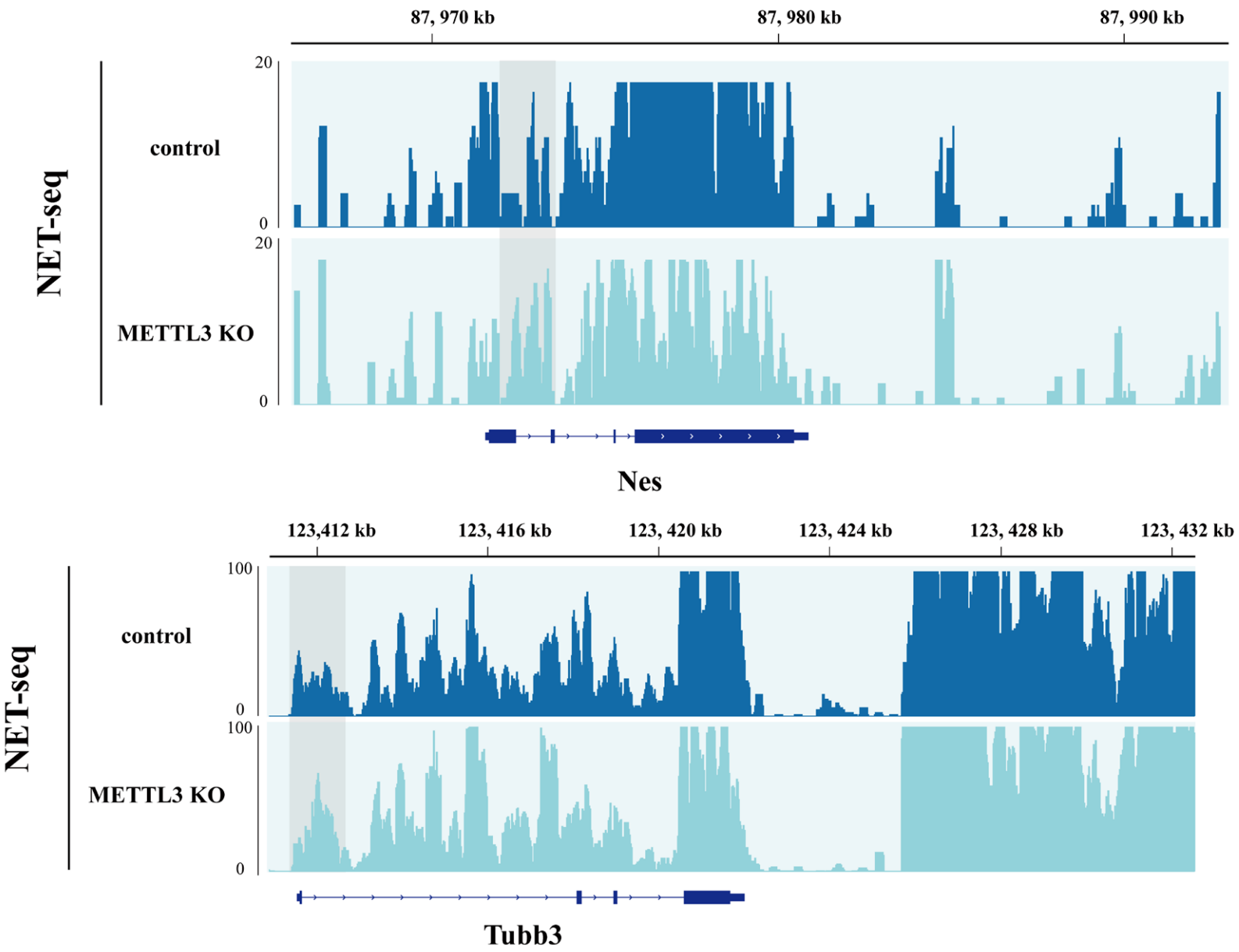

87, 970 kb
87, 980 kb
87, 990 kb
NET-seq
control
METTL3 KO
20
0
Nes
123,412 kb
123, 416 kb
123, 420 kb
123, 424 kb
123, 428 kb
123, 432 kb
100
Tubb3

Figure S8: Inhibition of SEC specifically impair the differentiation of ESC to neuroectoderm lineage A: Proportional analysis of genes in each cluster between RA_72h and RA_0h. Each cluster is represented by the indicated color. Fisher's exact test was conducted to compare Cluster I and Cluster IV. p represents the p-value, and OR indicates the odds ratio. B: Comparison of RNA Pol II density at paused genes (Cluster IV from Figure 8C; top) and released genes (Cluster I from Figure 8C; bottom) between RA_72h and RA_0h. C: GO analysis of genes exhibiting transcription pause with upregulation and transcription release with upregulation in RA_72h versus RA_0h. D: Western blot showing AFF4 protein levels, component of SEC, in undifferentiated mESCs upon KL-2 treatment. The original blots are provided in Supplemental files 3. E: Immunofluorescence showing the expression of OCT4, a pluripotency marker, in undifferentiated mESCs treated with KL-2. Scale bar = 50 μm. F: Phase-contrast images showing colony morphology of undifferentiated mESCs following KL-2 treatment. Scale bar = 100 μm. G: CCK-8 assay measuring proliferation of undifferentiated mESCs after KL-2 treatment. H: Immunofluorescence images showing TUJ1 and NESTIN expression following directed neuroectodermal differentiation of established miPSCs in the DMSO- and KL-2-treated groups. Histograms depict average fluorescence intensity for each marker. Scale bar = 100 μm. I: NET-seq browser tracks depicting nascent transcript occupancy at Nes and Tubb3 loci in METTL3 knockout mESCs. Bars represent the mean ± SD from n = 3 independent experiments. ns: not significant, *** $P < 0.001$ by two-tailed Student's t-test.

Supplemental file 2: Information of antibody, primers, siRNA/shRNA, probes and data accession

Section 1: Antibodies information:

Antibodies	Source	Identifier
Mouse monoclonal anti-Alpha Smooth muscle actin	proteintech	67735-1-Ig
Mouse monoclonal anti-alpha Tubulin	proteintech	39527
Mouse monoclonal anti-alpha Tubulin	proteintech	61157
Mouse monoclonal anti-Beta Actin	proteintech	66009-1-Ig
Mouse monoclonal anti-GAPDH	proteintech	60004-1-Ig
Mouse monoclonal anti-OCT4	CST	#75463
Mouse monoclonal anti-TUBB3/TUJ1	proteintech	66375-1-Ig
Multi-rAb HRP-Goat Anti-Mouse Recombinant Secondary Antibody (H+L)	proteintech	RGAM001
Multi-rAb HRP-Goat Anti-Rabbit Recombinant Secondary Antibody (H+L)	proteintech	RGAR001
Phospho-(Ser/Thr) Phe Antibody	CST	#9631
Rabbit monoclonal anti-ELL	CST	#14468
Rabbit monoclonal anti-m6A	CST	#56593
Rabbit monoclonal anti-METTL3	CST	#86132
Rabbit monoclonal anti-SOX2	CST	#23064
Rabbit monoclonal RNA polymerase IICTD repeat YSPTSPS-ChIP Grade	Abcam	ab300575
Rabbit Recombinant Monoclonal NELFE antibody	Abcam	ab170104
Rabbit Polyclonal anti-AFF4	proteintech	14662-1-AP

Section 2: Primer information (for qPCR):

ELL-F	TCCATCCGATTTGAAGGAAGC
ELL-R	ACATCCCCATAGCTGGATACAT
Gapdh-F	AGGTCGGTGTGAACGGATTTG

Gapdh-R	TGTAGACCATGTAGTTGAGGTCA
Klf4-F	CTATGCAGGCTGTGGCAAAACC
Klf4-R	TTGCGGTAGTGCCTGGTCAGTT
Mettl3-F	CTGGGCACTTGGATTTAAGGAA
Mettl3-R	TGAGAGGTGGTGTAGCAACTT
Myc-F	CCCTATTTCATCTGCGACGAG
Myc-R	GAGAAGGACGTAGCGACCG
Nanog-F	TCTTCCTGGTCCCCACAGTTT
Nanog-R	GCAAGAATAGTTCTCGGGATGAA
Nes-F	AGGAGAAGCAGGGTCTACAGAG
Nes-R	AGTTCTCAGCCTCCAGCAGAGT
Oct4-F	CGGAAGAGAAAGCGAACTAGC
Oct4-R	ATTGGCGATGTGAGTGATCTG
Orc1-F	TCATCGTGCCACTCCTCAGATC
Orc1-R	GAGAGAGTCAGGCACAGCAGAA
Pax6-F	CTGAGGAACCAGAGAAGACAGG
Pax6-R	CATGGAACCTGATGTGAAGGAGG
Sall1-F	CTCAACATTTCCAATCCGACCC
Sall1-R	GGCATCCTTGCTCTTAGTGGG
SELECT -F	TAGCCAGTACCGTAGTGCGTG
SELECT-R	ATGCAGCGACTCAGCCTCTG
Sox2-F	AACGGCAGCTACAGCATGATGC

Sox2-R	CGAGCTGGTCATGGAGTTGTAC
Tubb3-F	TAGACCCCAGCGGCAACTAT
Tubb3-R	GTTCCAGGTTCCAAGTCCACC

Section 3: Primer information (for ChIP-qPCR):

Klf4-body-F	CGGGACTCAGTGTAGGGGTA
Klf4-body-R	CCCTAGAGGCCCATTTGAGC
Klf4-promoter-F	CATGTCAGACTCGCCAGGTG
Klf4-promoter-R	GGGAACAGAAAGGGGTGAGG
Myc-body-F	AGTGCATTGACCCCTCAGTG
Myc-body-R	GTGTCTCCTCATGCAGCACT
Myc-promoter-F	GGTTGGAAGAGCCGTGTGT
Myc-promoter-R	TGATGTTGGGTCAGTCGCAG
Nes-body-F	TTGCTCAGGGAATGCCAGAG
Nes-body-R	TTGGGTCCTCTAGCCCTACC
Nes-promoter-F	CGGCGGGAGTATGAATACCC
Nes-promoter-R	GCACTAGAGAAGGGAGTGGC
Tubb3-body-F	TGCATCGACAATGAAGCCCT
Tubb3-body-R	CATCGAACATCTGCTGCGTG
Tubb3-promoter-F	CAGACTTGCTCCGCATTGTG
Tubb3-promoter-R	CCTTGGCCCCTATCTGGTTG

Section 4: siRNA/shRNA information:

siAFF4-F	GCAACGAAUGUUCUCUCCUAUdTdT
siAFF4-R	AUAGGAGAGAACAUUCGUUGCdTdT
siELL-F	GCUCUGAUGAGUACGAGACAAdTdT
siELL-R	UUGUCUCGUACUCAUCAGAGCdTdT
pFB-9-shELL(mouse)	TGAGACCTTCCATCCGATTTG

Section 5: SELECT probes

Klf4-A701-Down	**5phos**/CTTCTCCCTTCCCGCCGGGCCAGAGGCTGAGTCGCTGCAT
Klf4-A701-Up	TAGCCAGTACCGTAGTGCGTGGGCACCTGCTGGACGCAGTG
Klf4-A855-Down	**5phos**/TACTGCTGCAAGCTGCACCACAGAGGCTGAGTCGCTGCAT
Klf4-A855-Up	TAGCCAGTACCGTAGTGCGTGGGGCTAGGAGGGCCGGGTTG
Klf4-A1710-Down	**5phos**/AATGGAGAGAGGGGACTTGTCAGAGGCTGAGTCGCTGCAT
Klf4-A1710-Up	TAGCCAGTACCGTAGTGCGTGCTTCCCCCCACTAACCTTGA
Myc-A517-Down	**5phos**/CCCCAAATAGACAAAAATTCCAGAGGCTGAGTCGCTGCAT
Myc-A517-Up	TAGCCAGTACCGTAGTGCGTGGGCAGAGGCAGAGAACACTG
Myc-A1672- Down	**5phos**/CTGAGGACCTGGGGCTGGAGCAGAGGCTGAGTCGCTGCAT
Myc-A1672-Up	TAGCCAGTACCGTAGTGCGTGTCTTGTCGTTTTCCTCCGTG
Myc-A1410- Down	**5phos**/TTCTTCCTCATCTTCTTGCTCAGAGGCTGAGTCGCTGCAT
Myc-A1410-Up	TAGCCAGTACCGTAGTGCGTGCTCCACAGACACCACATCAA

Section 6: Data avaliability:

RNA-seq:

D2KL-2_D4 reprogramming	GSE297812

iPSC reprogramming	GSE70022
ESC_RA_differentiation	GSE185889
MEF vs ESC RNA-seq	GSE113430
HLC vs hiPSC RNA-seq	GSE138312
METTL3 KO vs WT	GSE325527
**ChIP-seq/CUT&Tag:**	
D4 _RNA Pol2 CUT&Tag_D2KL-2	GSE297813
E14_ES_RA0h_ xChIP.NTD	GSE261039
E14_ES_RA24h_ xChIP.NTD	GSE261039
E14_ES_RA48h_ xChIP.NTD	GSE261039
E14_ES_RA4h_ xChIP.NTD	GSE261039
E14_ES_RA6h_ xChIP.NTD	GSE261039
E14_ES_RA72h_ xChIP.NTD	GSE261039
H3K18la_CUT&Tag	GSE251641
Hela_RNAPII_ChIP_shCtrl	HRA002494 (National Genomics Data Center of the China National Center for Bioinformation)
Hela_RNAPII_ChIP_shMETTL3	HRA002494 (National Genomics Data Center of the China National Center for Bioinformation)
mES ELL ChIP-Seq	GSE32120
mESC_AFF4_0hr_ChIPSeq	GSE30267
METTL3_WT_METTL3_ChIP	GSE154135
RNAPII_ChipSeq_day0 (reprogramming)	GSE67520

RNAPII_ChipSeq_day5 (reprogramming)	GSE67520
RNAPII_ChipSeq_day11 (reprogramming)	GSE67520
RNAPII_ChipSeq_iPSC (reprogramming)	GSE67520

NET-seq:

ESC_METTL3_Control_10min	GSE133582
ESC_METTL3_KO_10min	GSE133582

GRO-seq:

HLCs_M1_11104	GSE117086
iPSC_11104_	GSE117086
MESC_GROseq	GSE27037
MEF_GROseq	GSE27037

MeRIP-seq:

D4 _MeRIP_D2KL-2	GSE297999
FBS, MeRIP m6A-IP	GSE202846
m6A-seq in ESCs	GSE61995
m6A-seq in Mettl3-KO ESCs	GSE61995

GLORI-seq

D4 _GLROI_D2KL-2	GSE341965

PDB:

AFF4	6K7P

ELL	2DOA
ENL	6HQ0
METTL3	5IL1
CDK9/cyclinT1	3BLH
WTAP	7YFJ

Supplemental file 3: Original blots images in this study

Figure 4F

Sample legend:

Lane	1	2	3	4
Sample	Not mentioned in manuscript	DMSO	KL-2	Not mentioned in manuscript

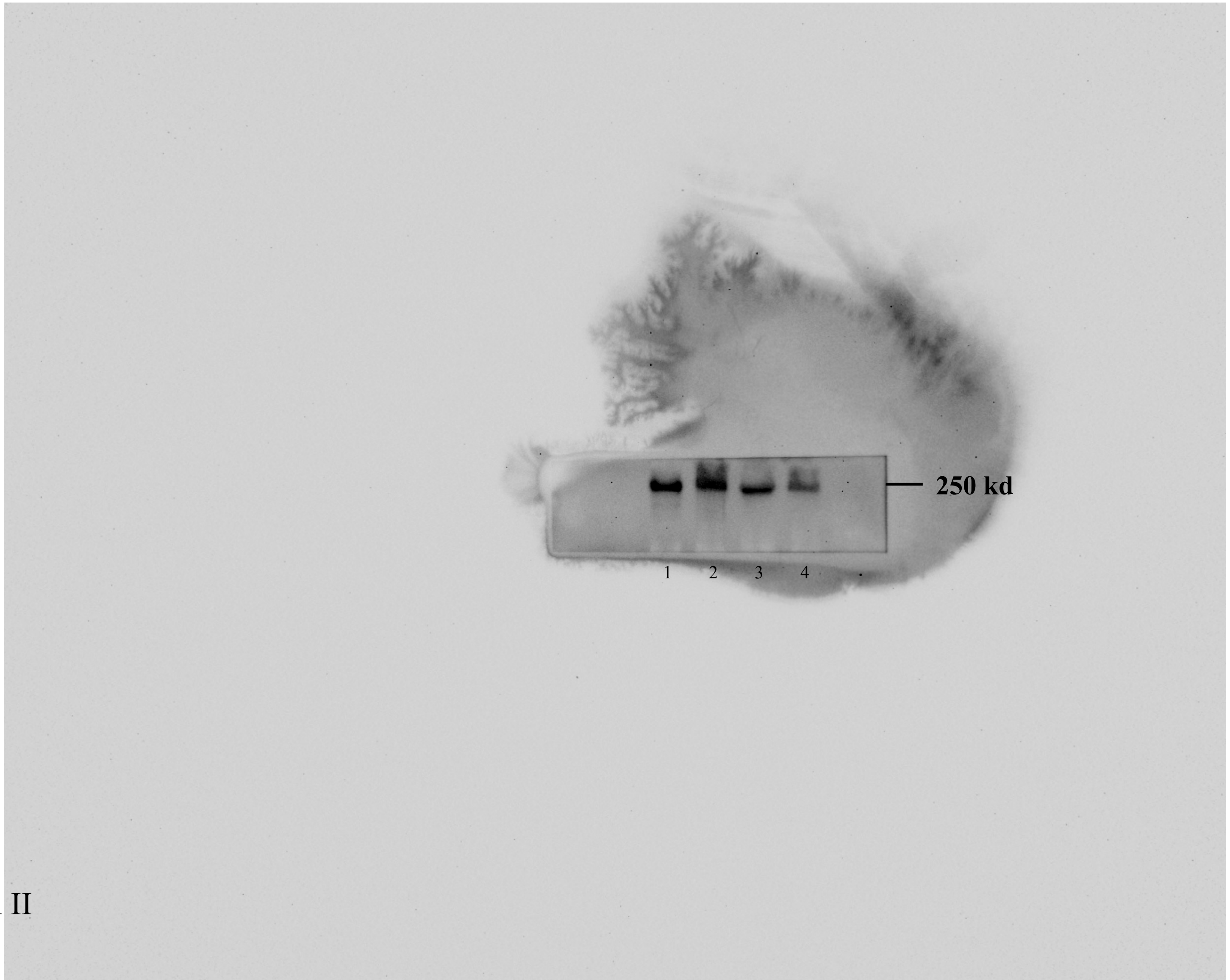

pSer2 RNA Pol II

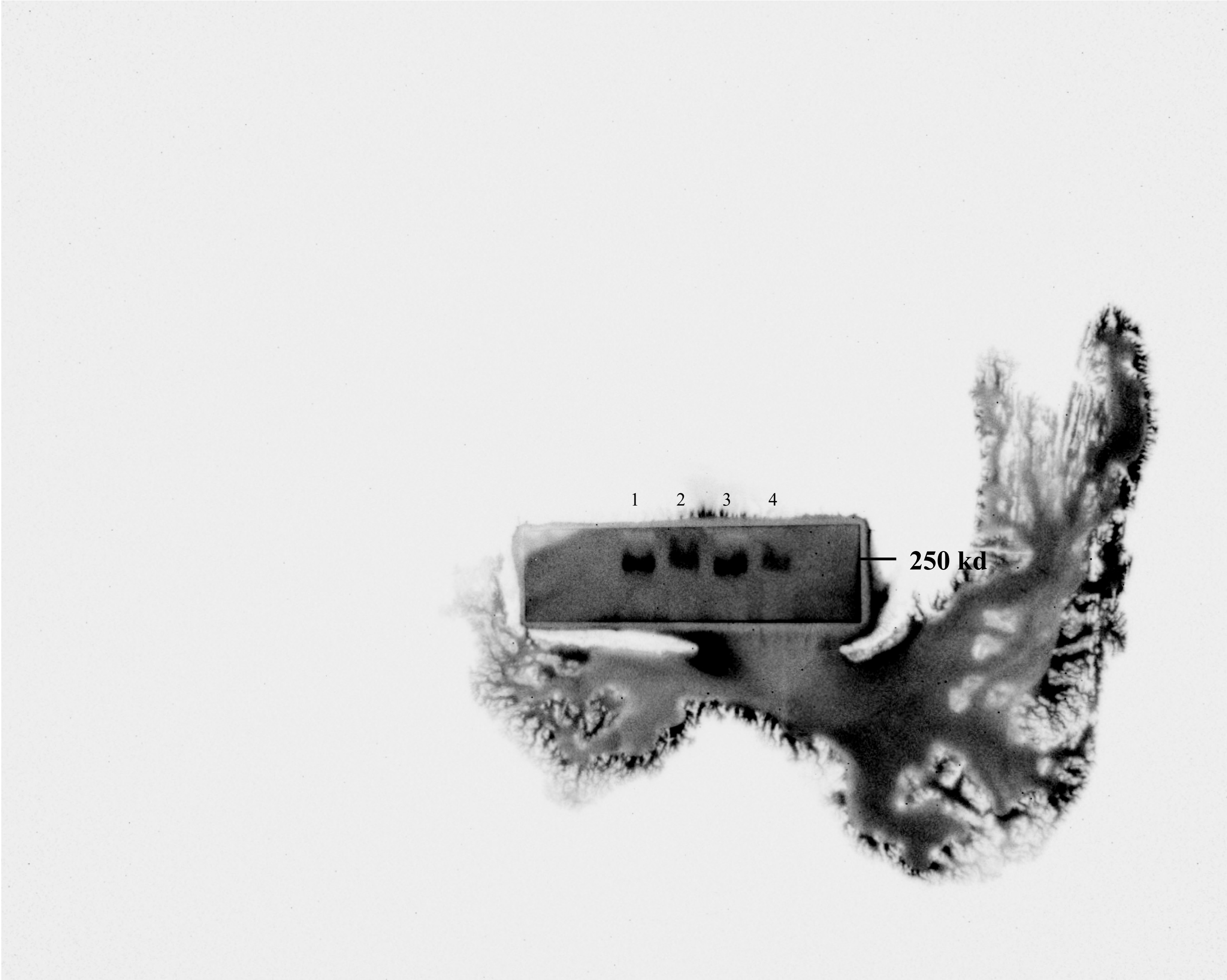

RNA Pol II

α-tubulin

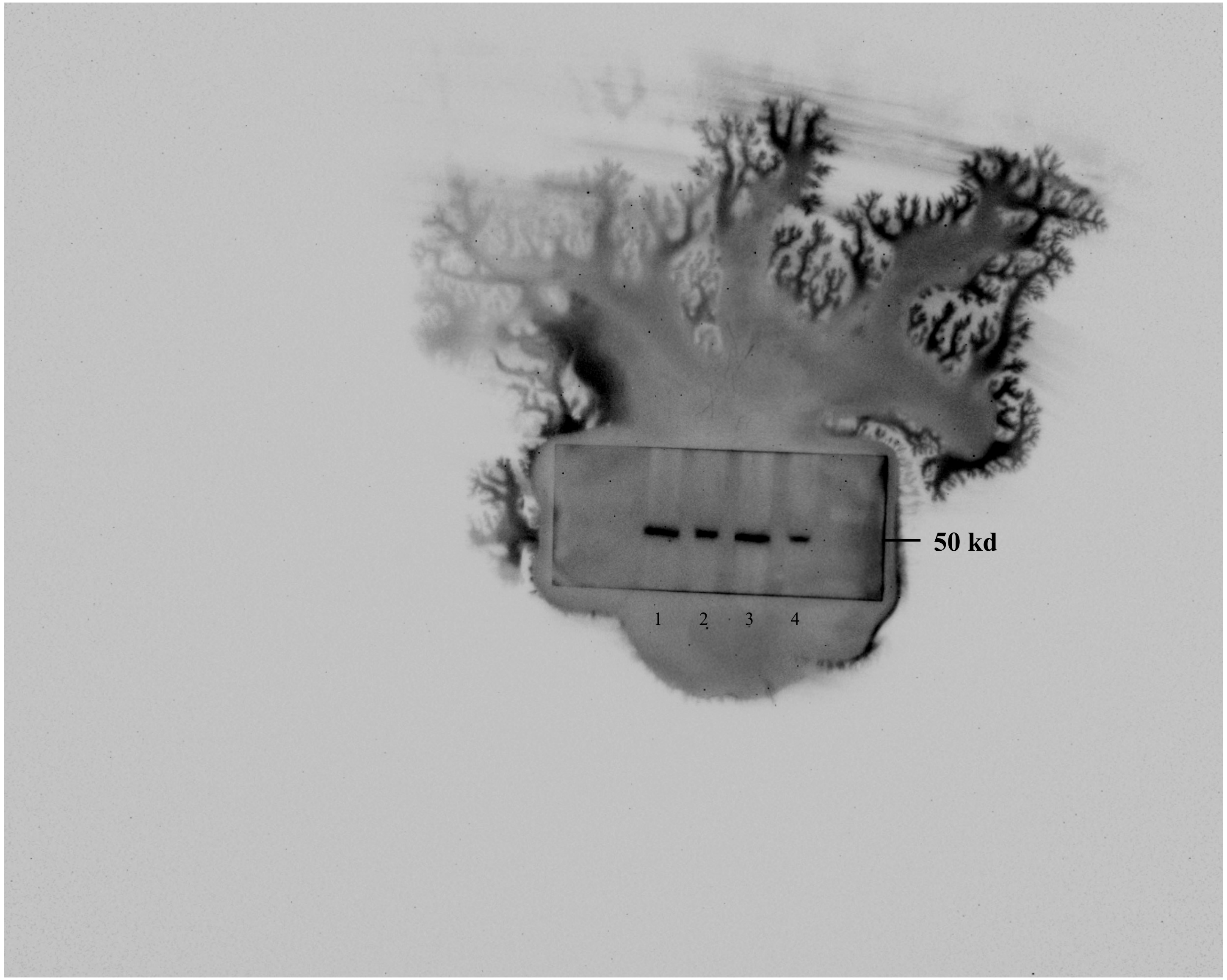

Figure 5A

Sample legend:

Lane	1	2
Sample	DMSO (1 μg)	KL-2 (1 μg)
	DMSO (0.5 μg)	KL-2 (0.5 μg)

m^6A

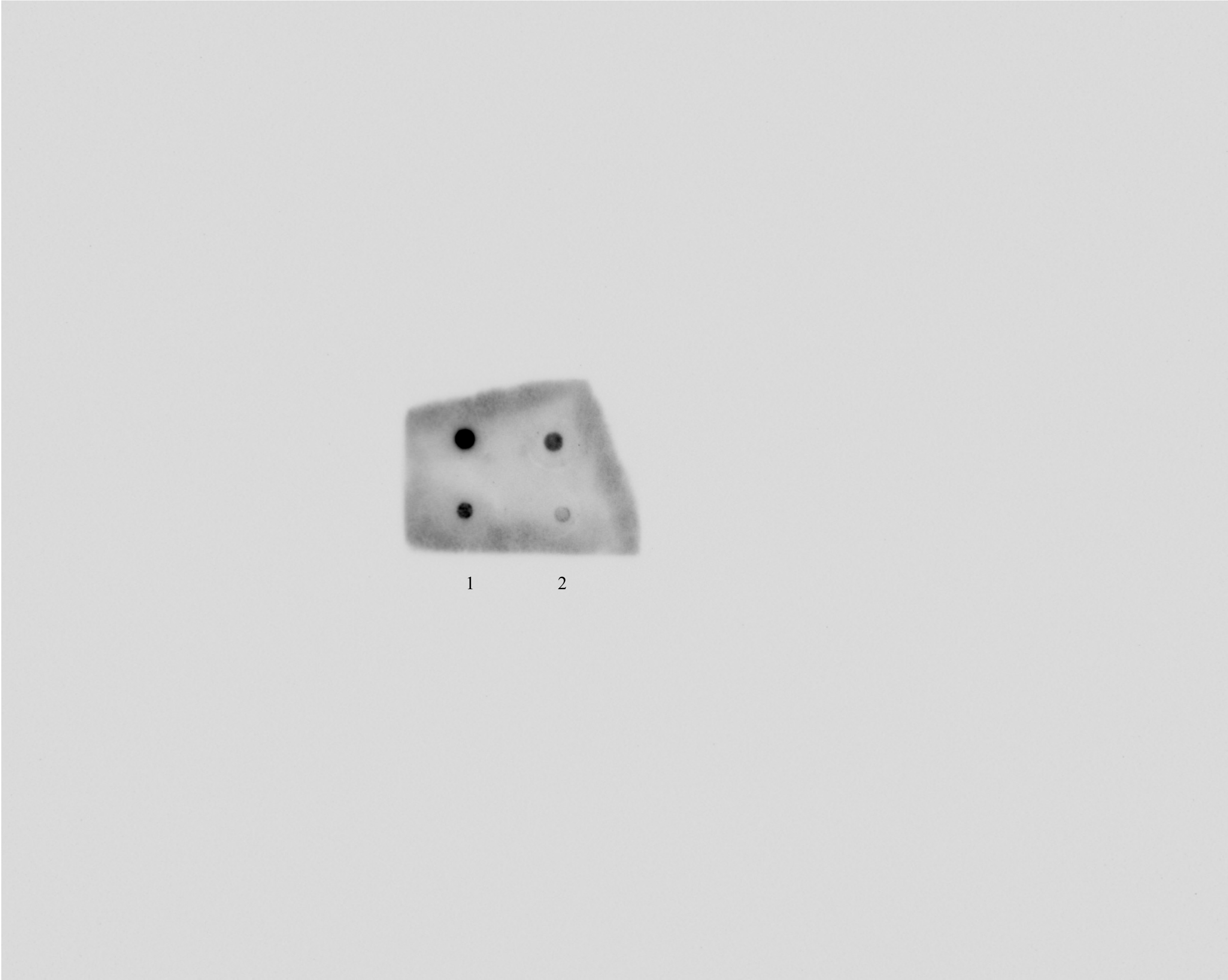

Figure 6C (METTL3 IP)

Sample legend:

Lane	1	2	3
Sample	Input	METTL3 IP	IgG

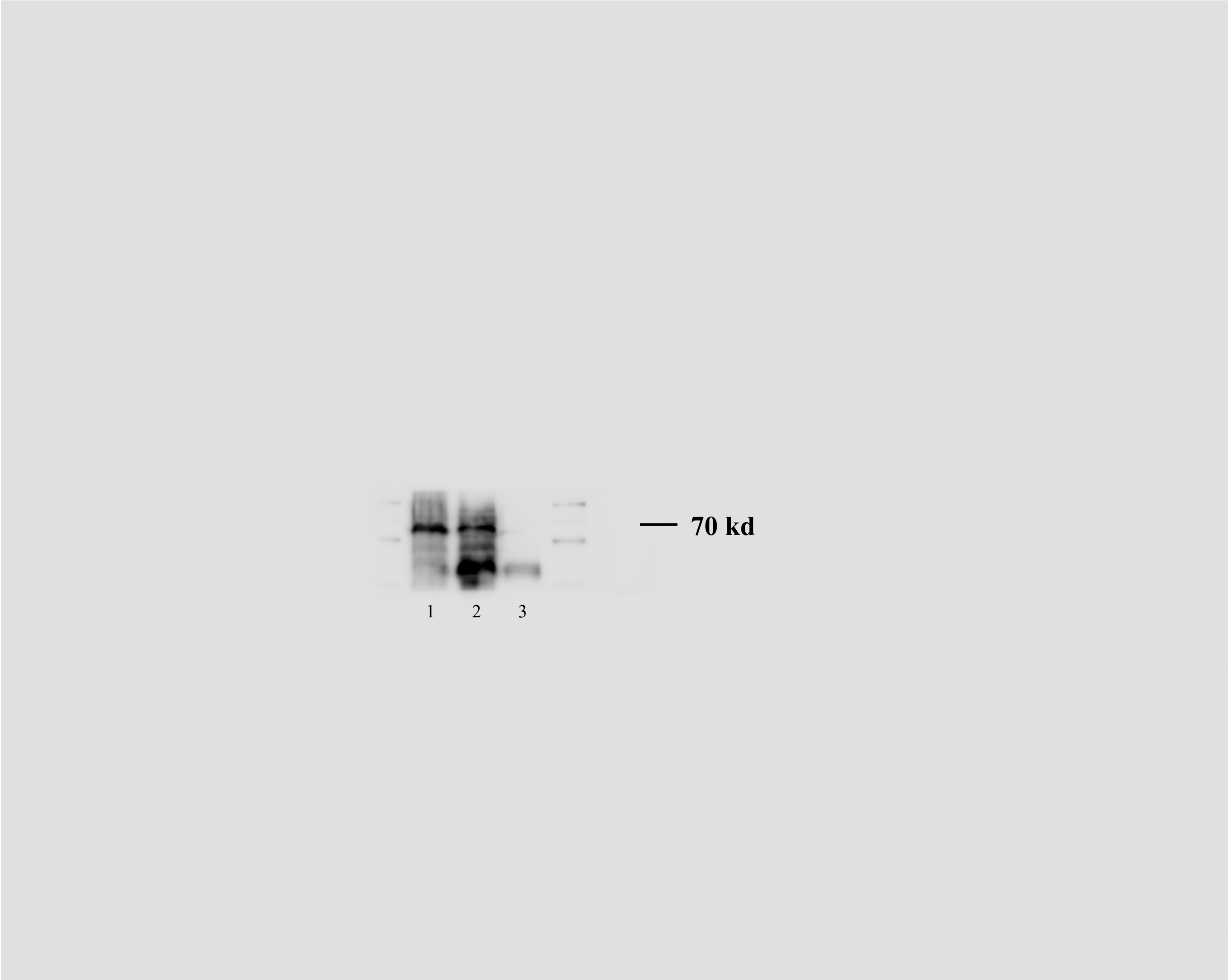

ELL

METTL3

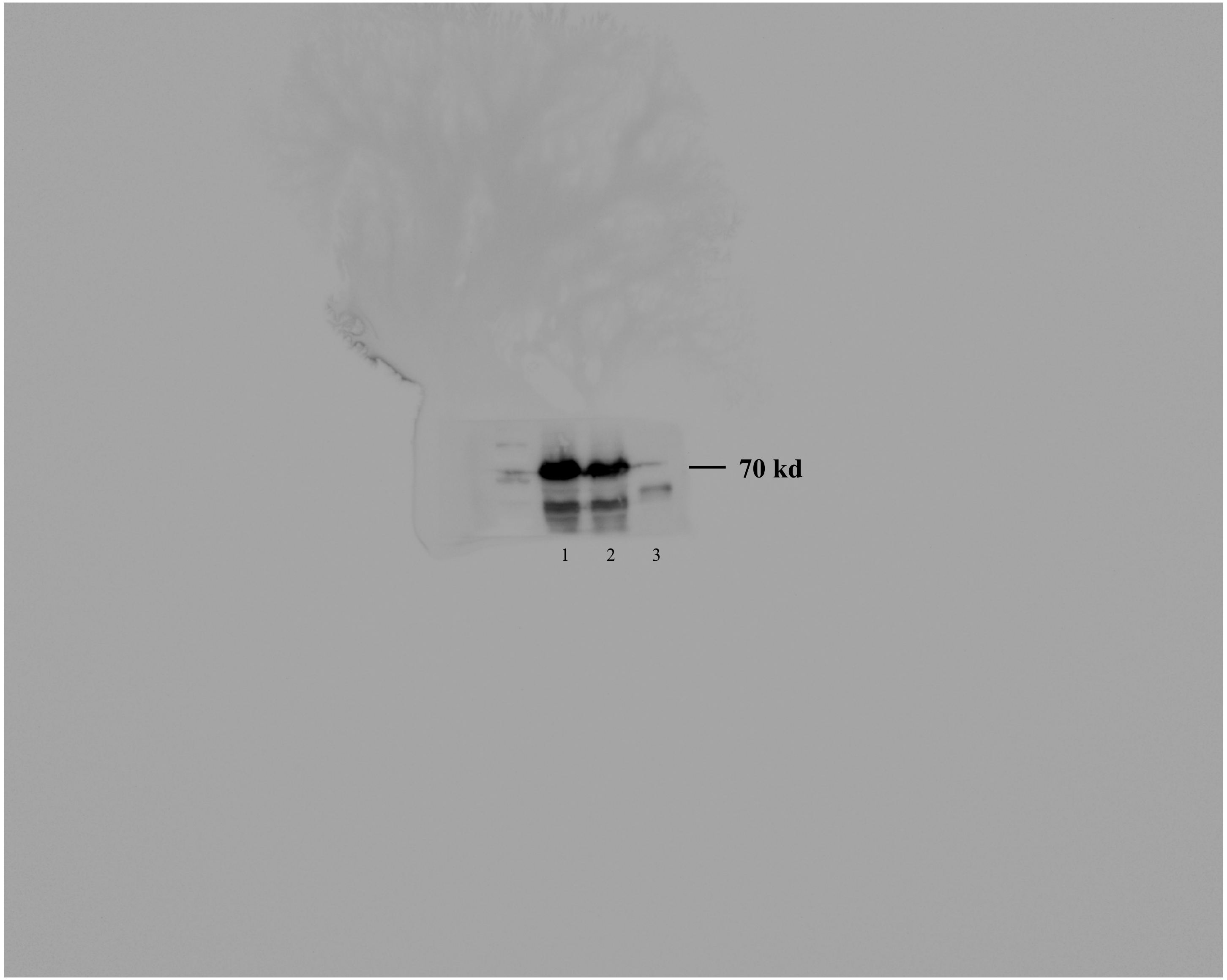

Figure 6C (ELL IP)

Sample legend:

Lane	1	2	3	4	5	6
Sample	Input	ELL IP	IgG	Not mentioned in manuscript	Not mentioned in manuscript	Not mentioned in manuscript

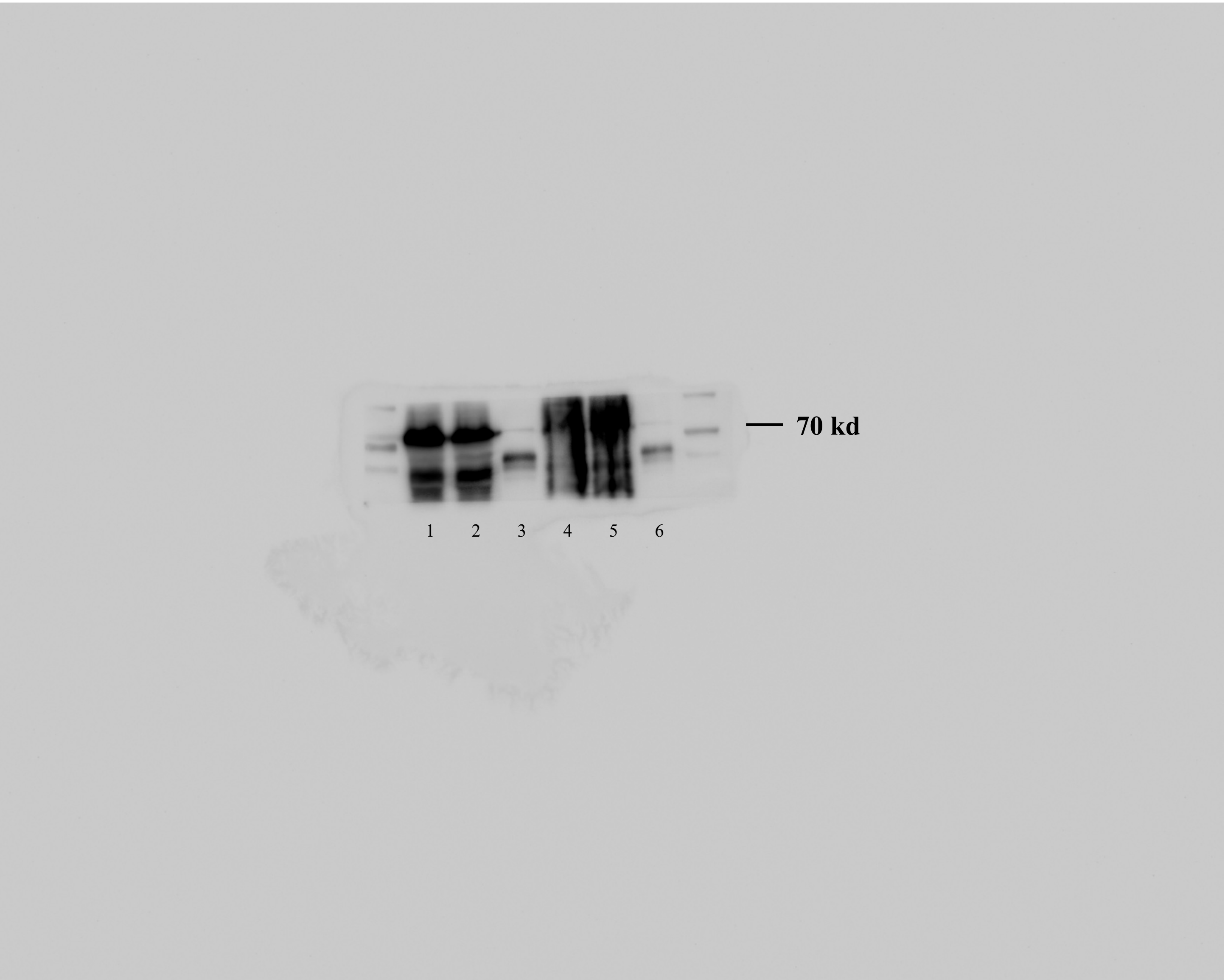

METTL3

Figure 6C (ELL IP)

Sample legend:

Lane	1	2	3
Sample	Input	ELL IP	IgG

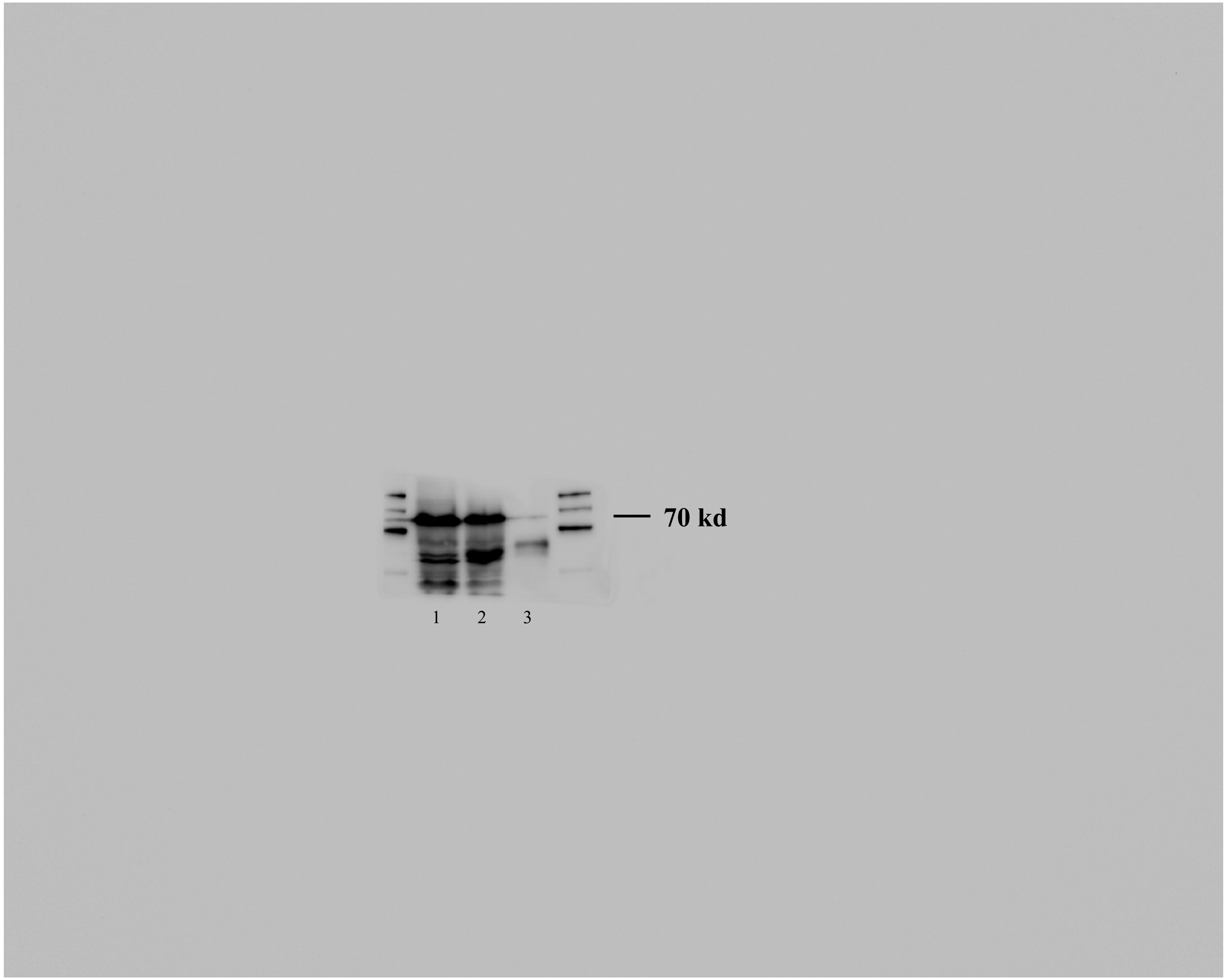

ELL

Figure 6D (ELL OE)

Sample legend:

Lane	1	2
Sample	control (400 ng)	ELL-OE (400ng)
	control (200ng)	ELL-OE (200ng)

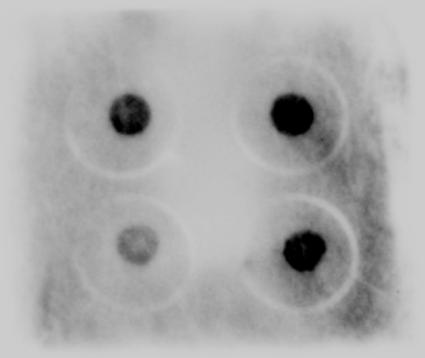

1 2

m^{6}A

Figure 6D (siELL)

Sample legend:

Lane	1	2	3	4
Sample	Not mentioned in manuscript	Not mentioned in manuscript	NC (1 μg)	siELL (1 μg)
	Not mentioned in manuscript	Not mentioned in manuscript	NC (0.5 μg)	siELL (0.5 μg)

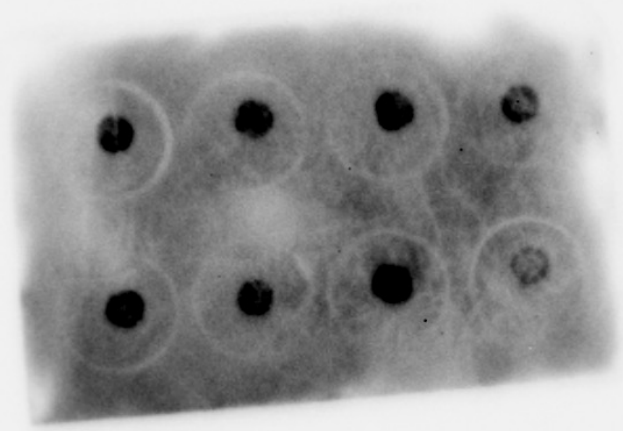

1 2 3 4

m^{6}A

Figure 6E

Sample legend:

Lane	1	2	3	4	5
Sample	DMSO (Input)	KL-2 (Input)	DMSO (Pol II IP)	KL-2 (Pol II IP)	IgG

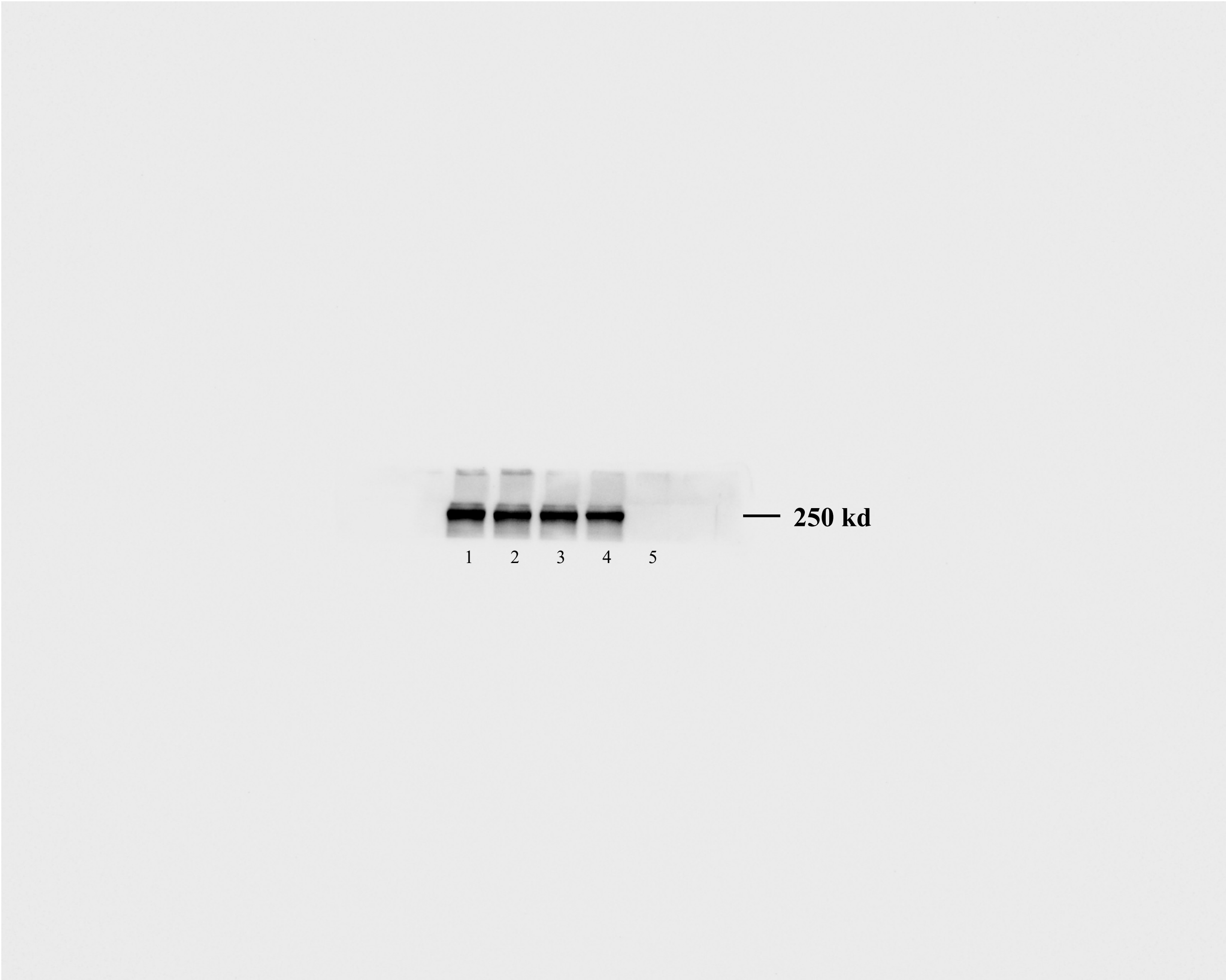

RNA Pol II

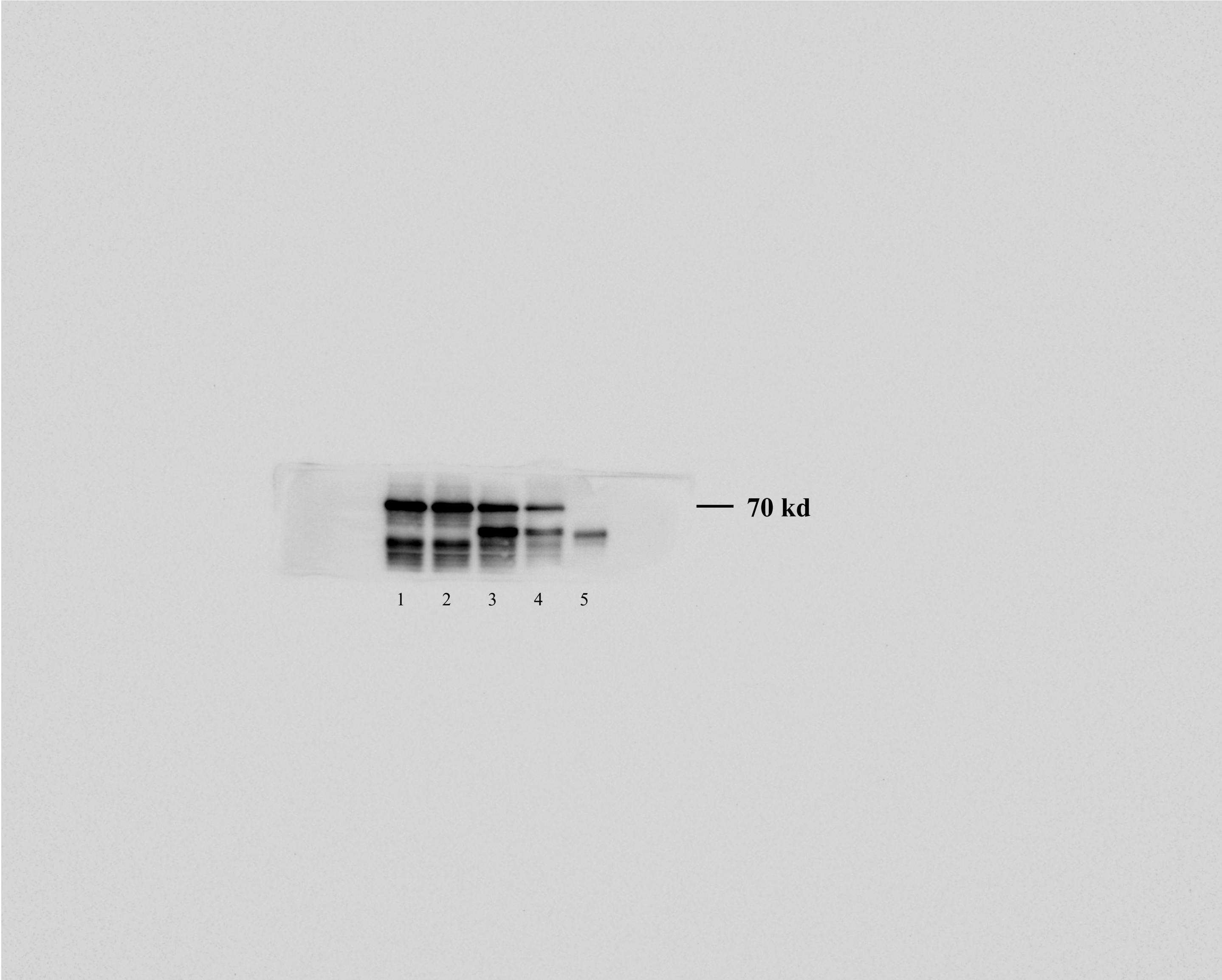

METTL3

Figure 7D

Sample legend:

Lane	1	2	3	4
Sample	control (DMSO, 400ng)	ELL-OE (DMSO, 400ng)	control (STM2457, 400ng)	ELL-OE (STM2457, 400ng)
	control (DMSO, 200ng)	ELL-OE (DMSO, 200ng)	control (STM2457, 200ng)	ELL-OE (STM2457, 200ng)

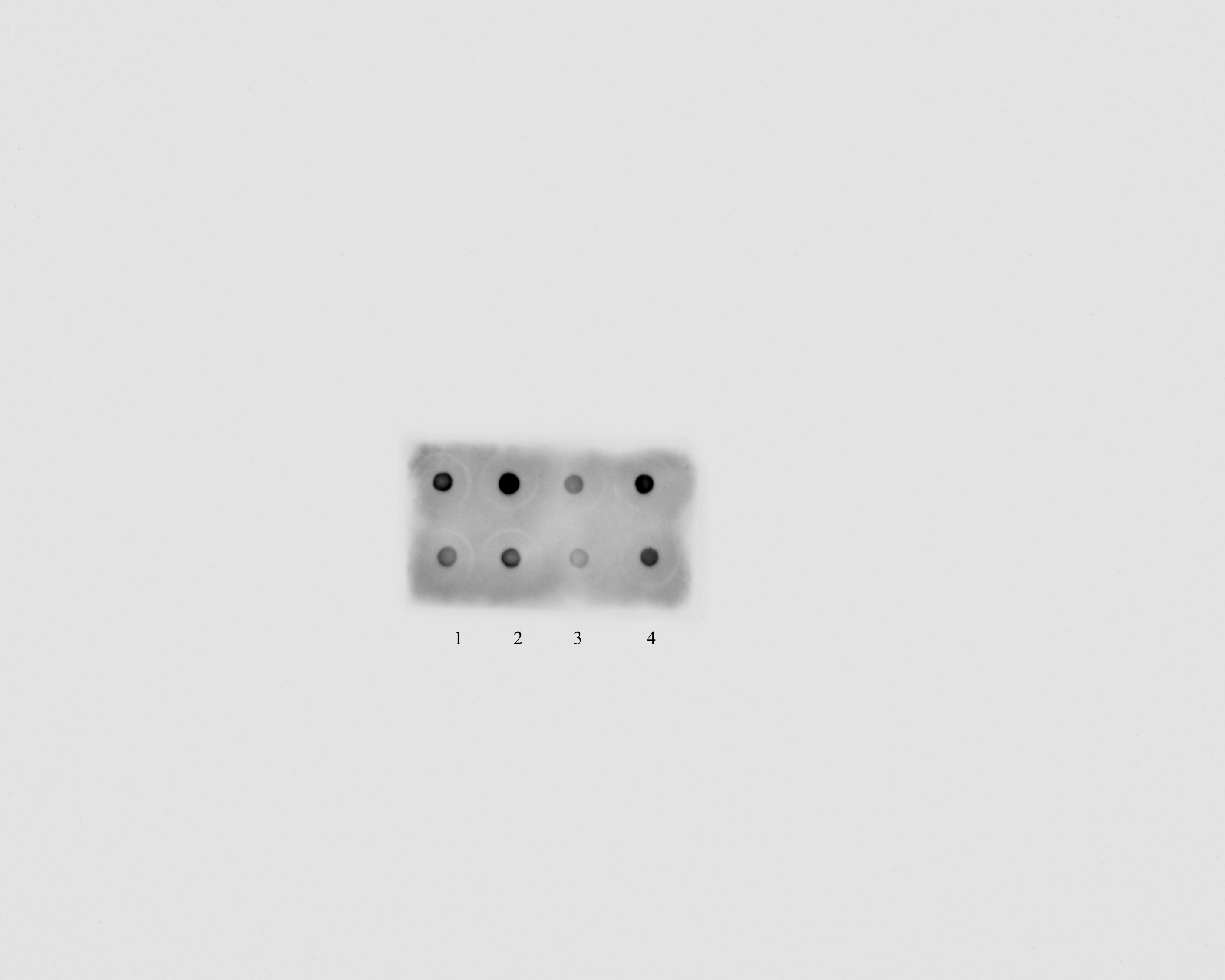

m6A

Figure 7E

Sample legend:

Lane	1	2	3	4
Sample	control (DMSO, 400ng)	METTL3-OE (DMSO, 400ng)	control (KL-2, 400ng)	METTL3-OE (KL-2, 400ng)
	control (DMSO, 200ng)	METTL3-OE (DMSO, 200ng)	control (KL-2, 200ng)	METTL3-OE (KL-2, 200ng)

m^{6}A

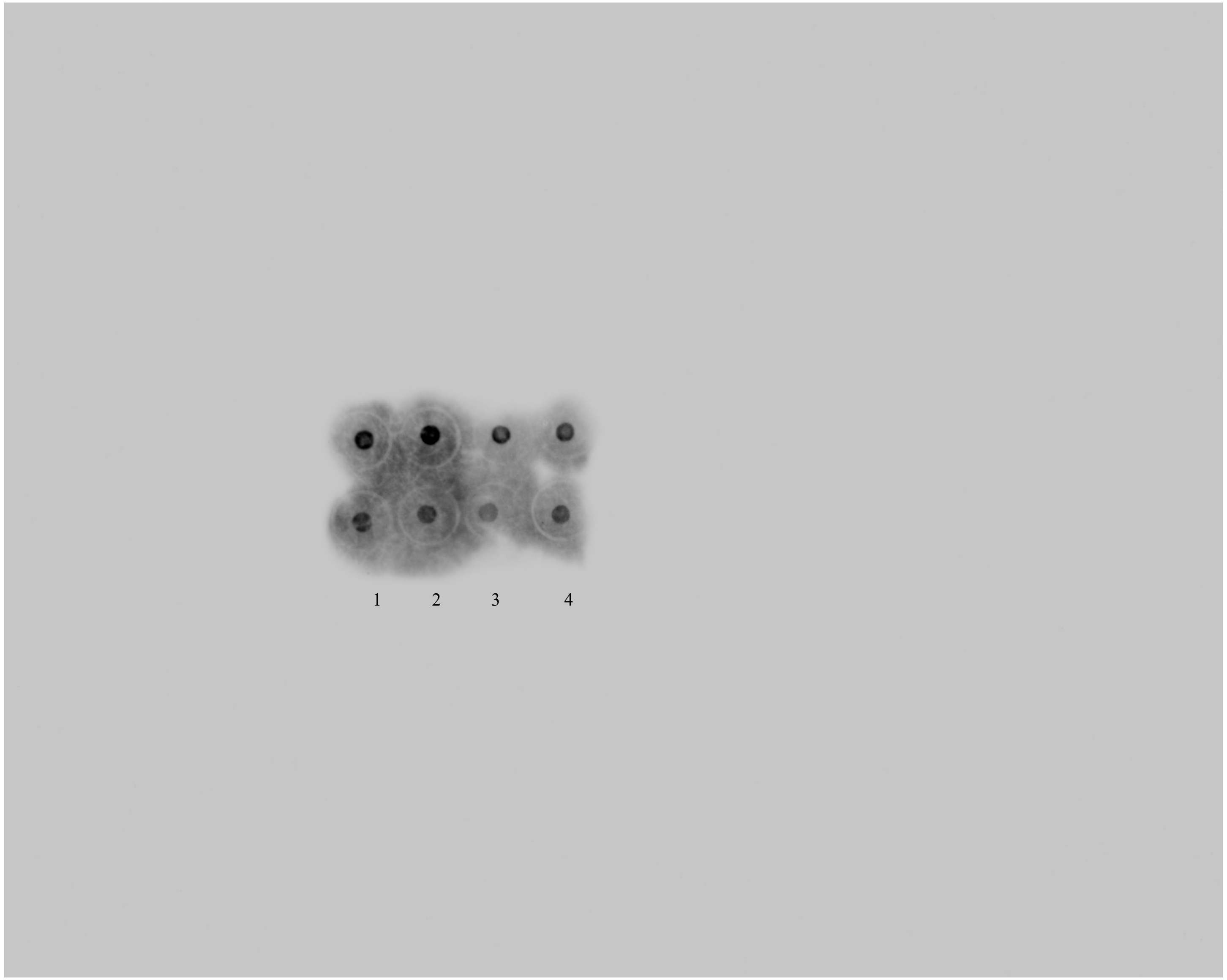

Figure 7I (Left)

Sample legend:

Lane	1	2	3	4	5	6
Sample	Not mentioned in manuscript	Not mentioned in manuscript	Not mentioned in manuscript	DMSO	STM2457	Not mentioned in manuscript

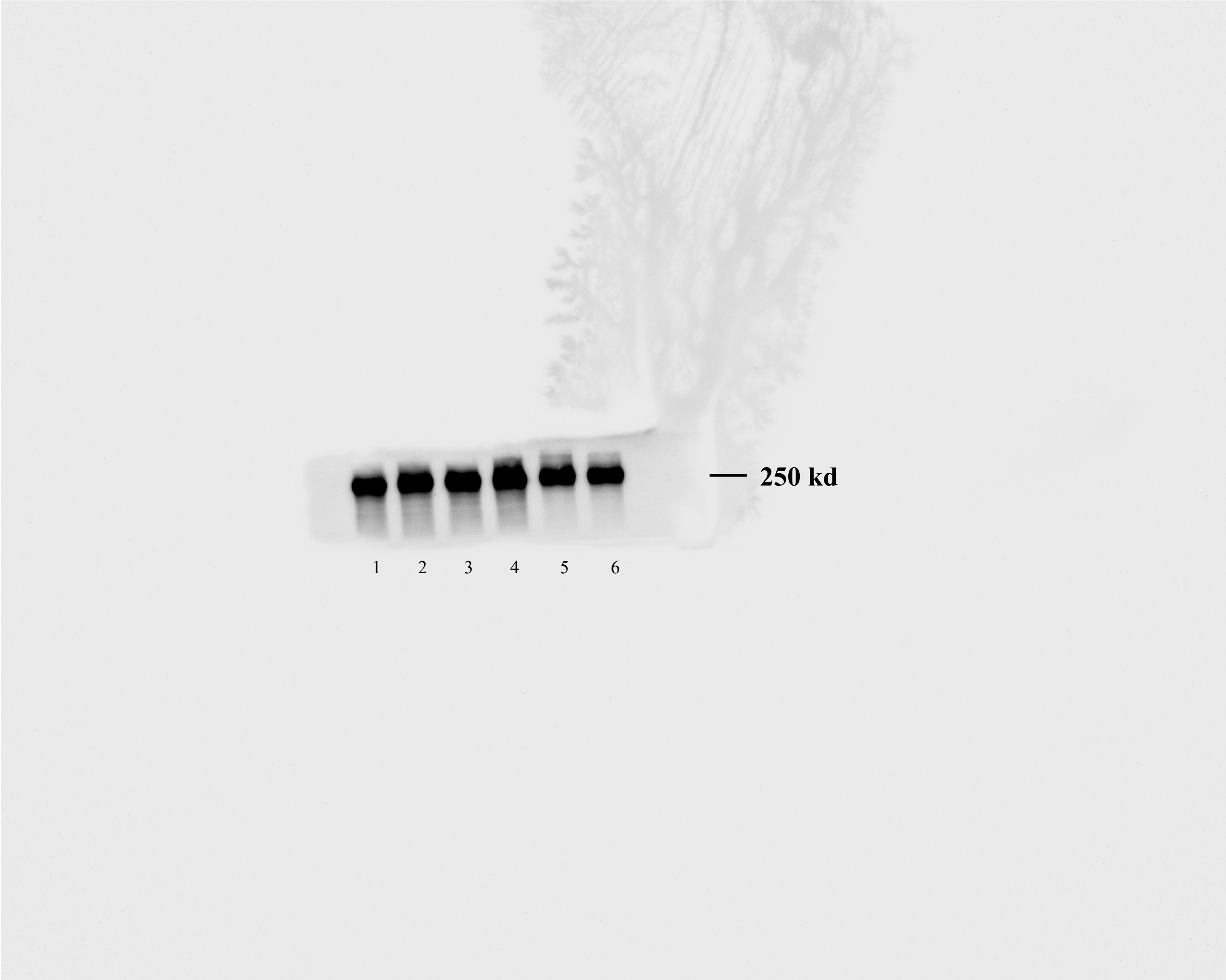

pSer2 RNA Pol II

β-actin

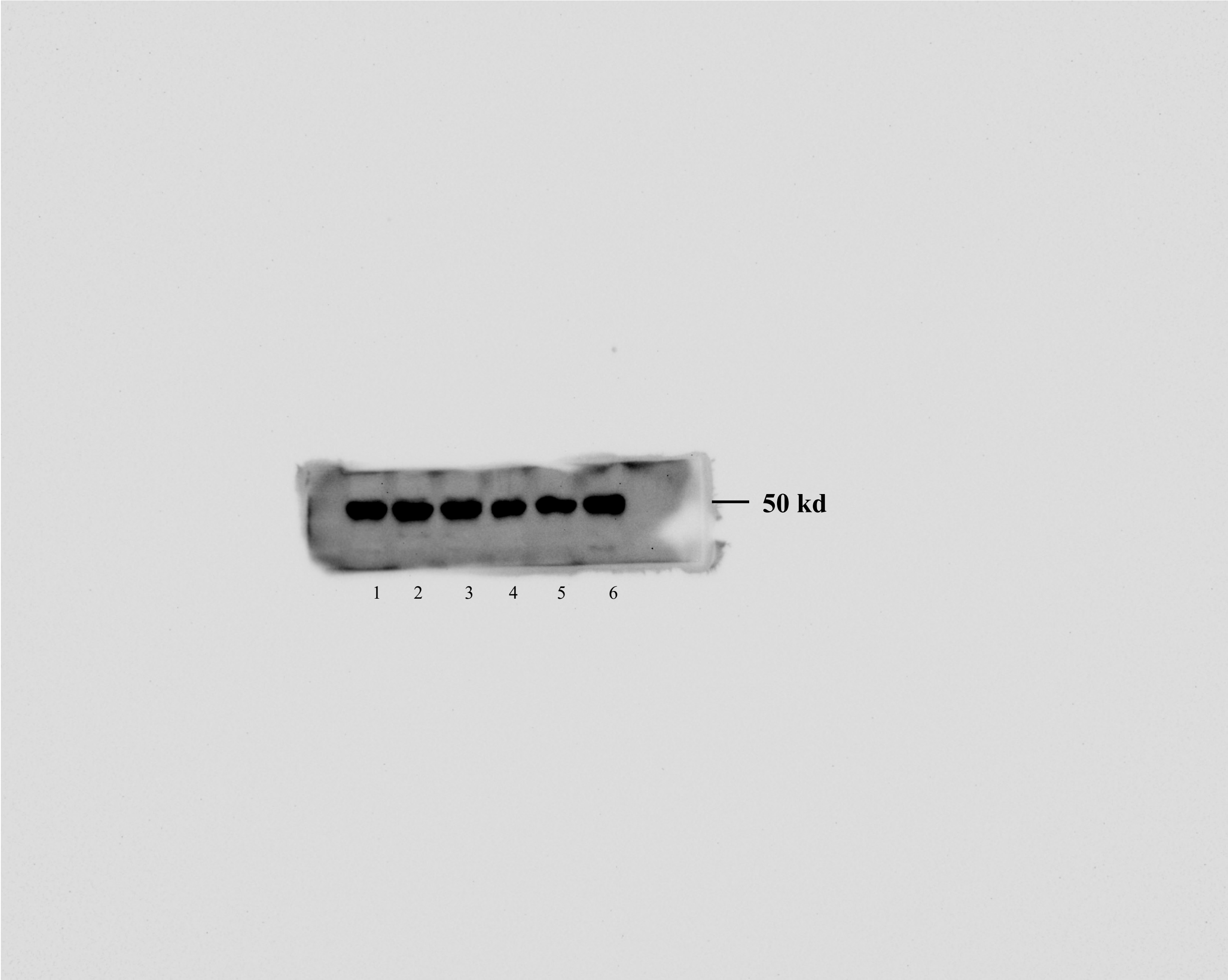

Figure 7I (Right)

Sample legend:

Lane	1	2
Sample	DMSO	STM2457

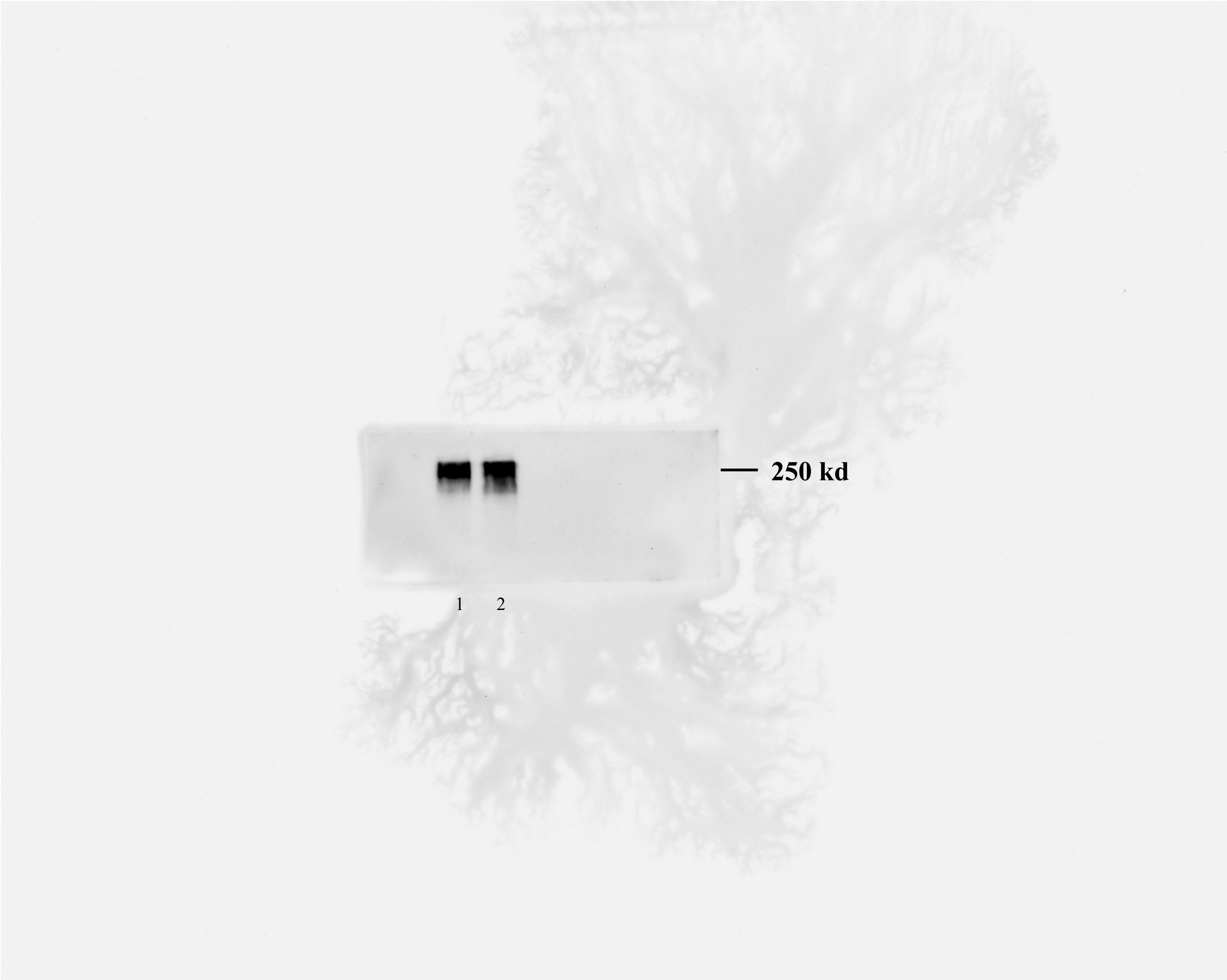

RNA Pol II

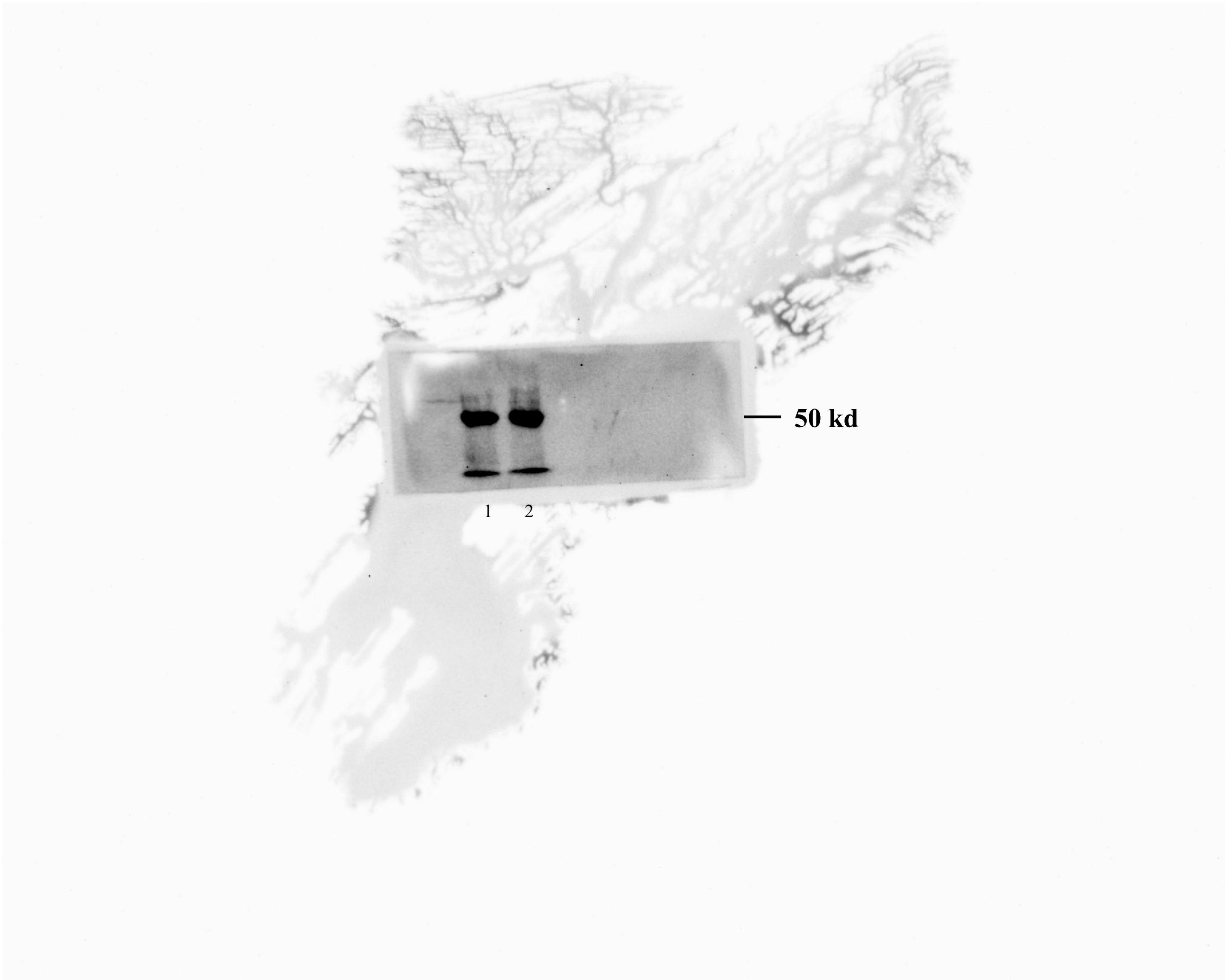

β-actin

Figure 8K

Sample legend:

Lane	1	2	3	4
Sample	Not mentioned in manuscript	Not mentioned in manuscript	DMSO	KL-2

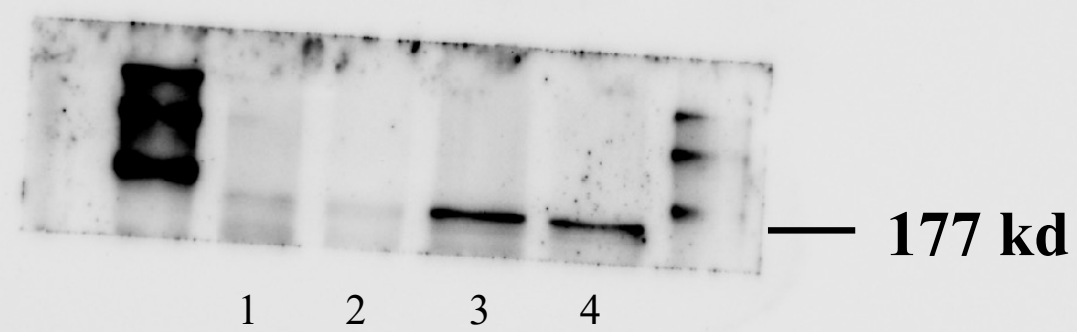

NESTIN

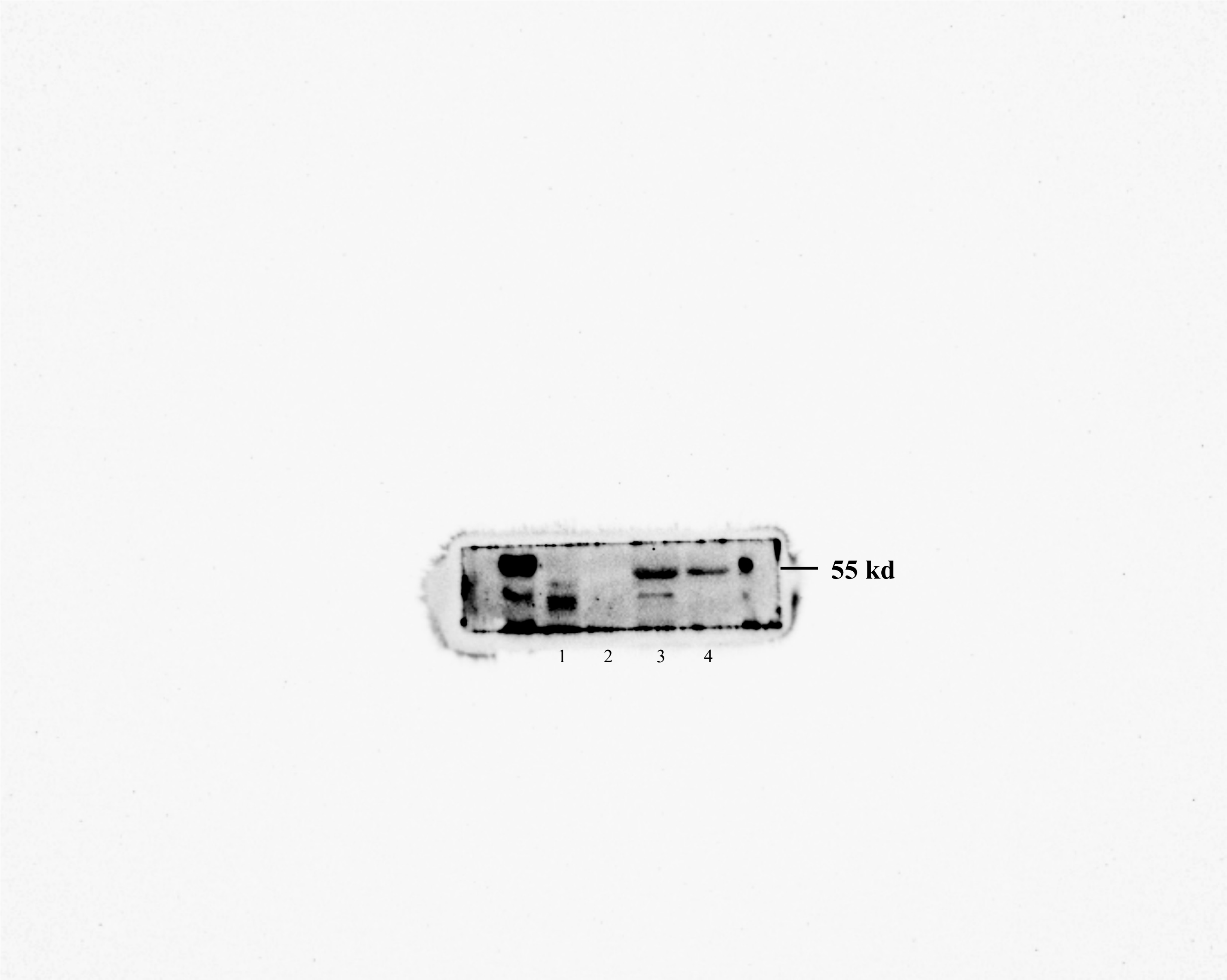

TUJ1

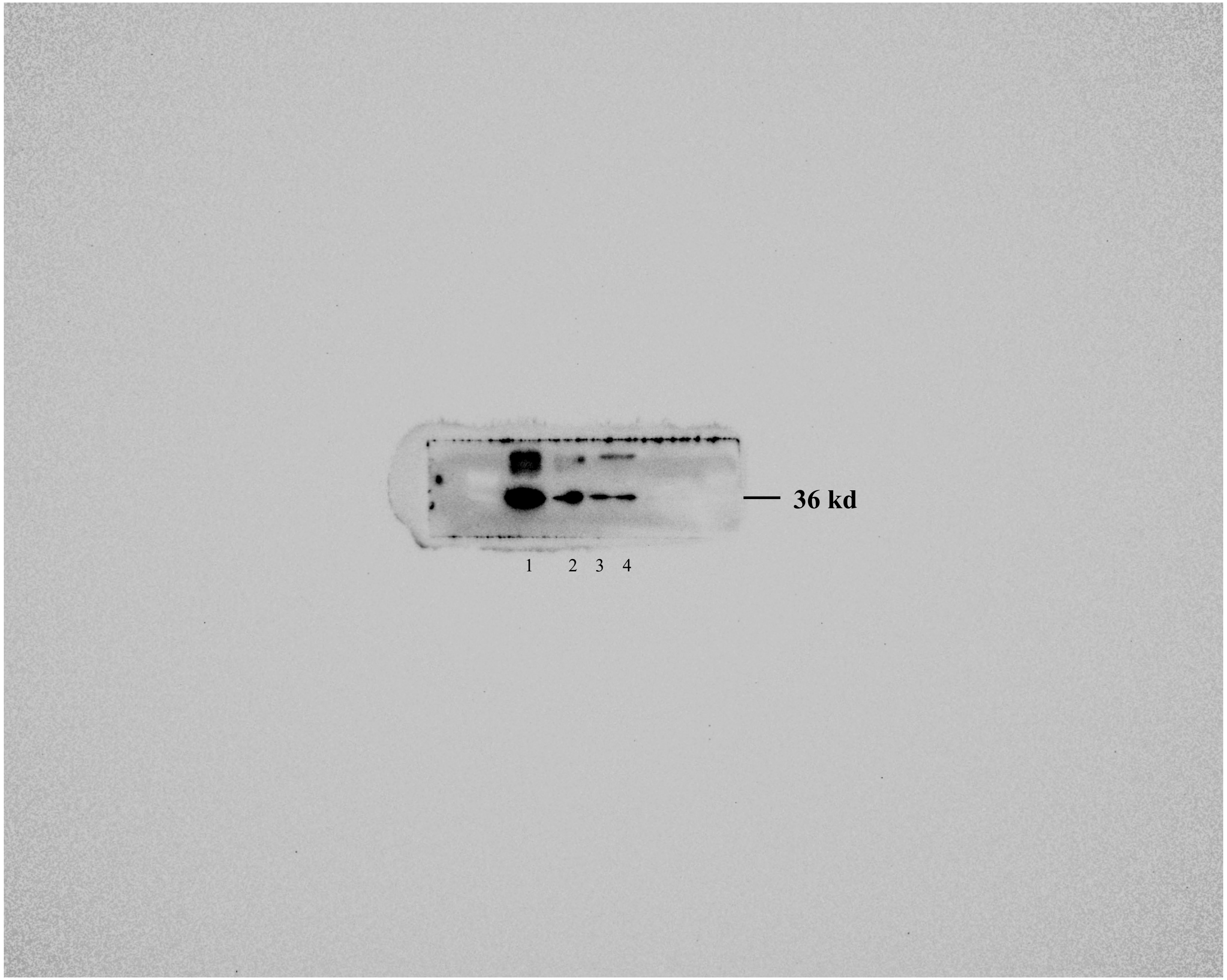

GAPDH

Figure 8N

Sample legend:

Lane	1	2	3	4
Sample	Not mentioned in manuscript	Not mentioned in manuscript	DMSO (400 ng)	KL-2 (400 ng)
	Not mentioned in manuscript	Not mentioned in manuscript	DMSO (200 ng)	KL-2 (200 ng)

m^6A

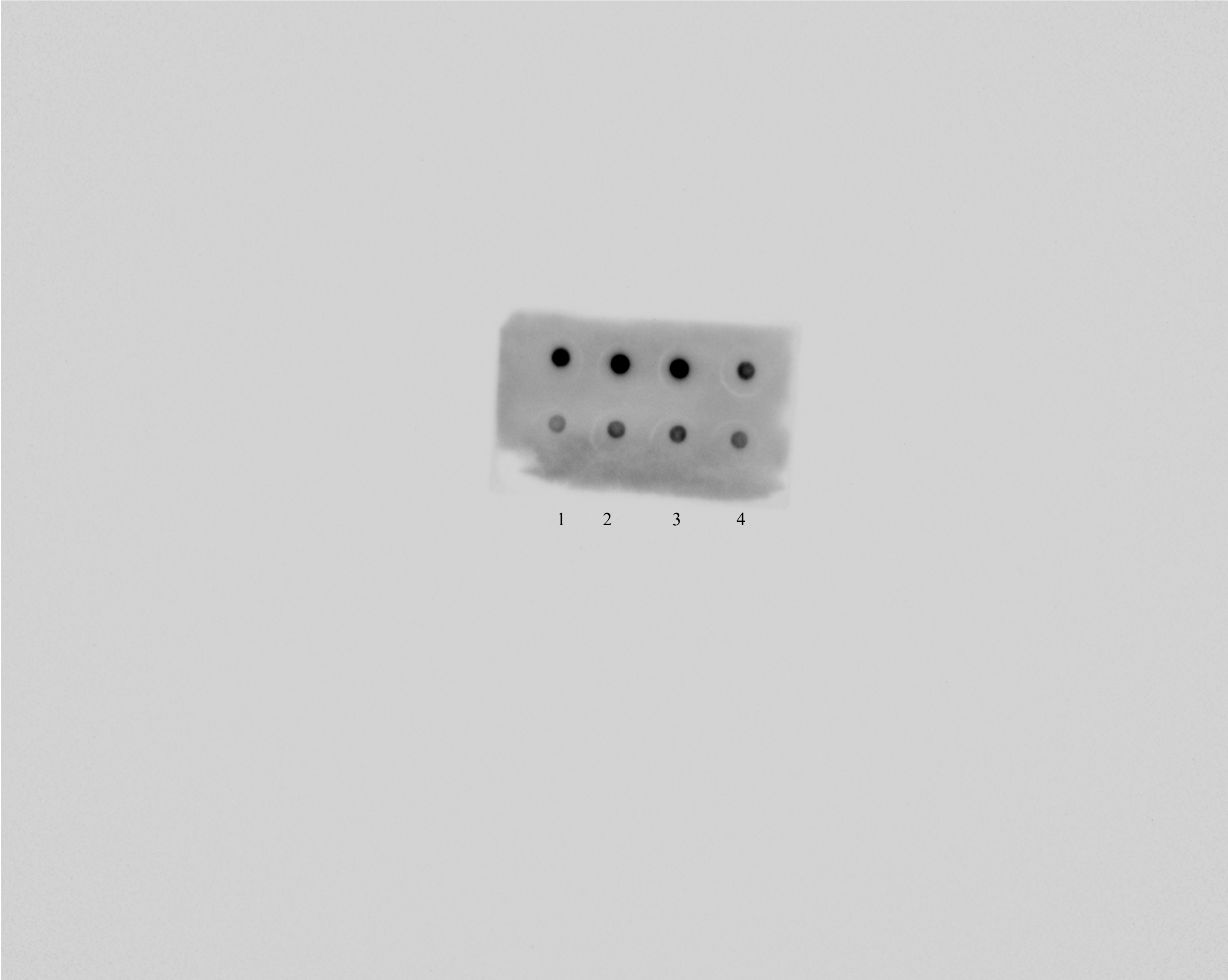

Figure S1A

Sample legend:

Lane	1	2	3	4	5	6
Sample	Not mentioned in manuscript	Not mentioned in manuscript	DMSO	KL-2	Not mentioned in manuscript	Not mentioned in manuscript

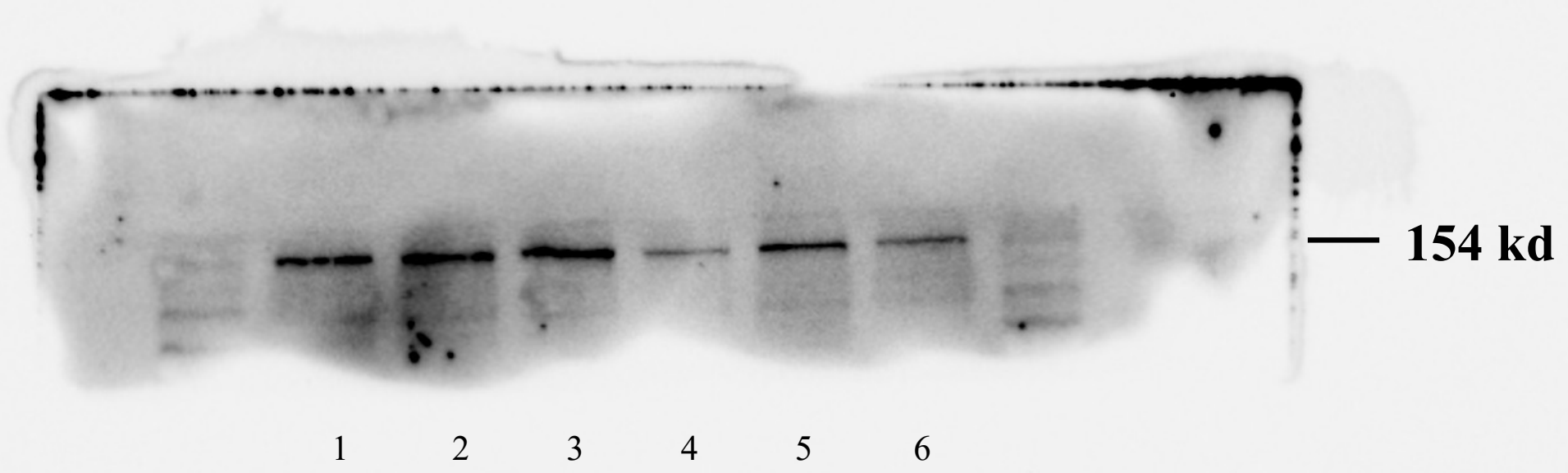

AFF4

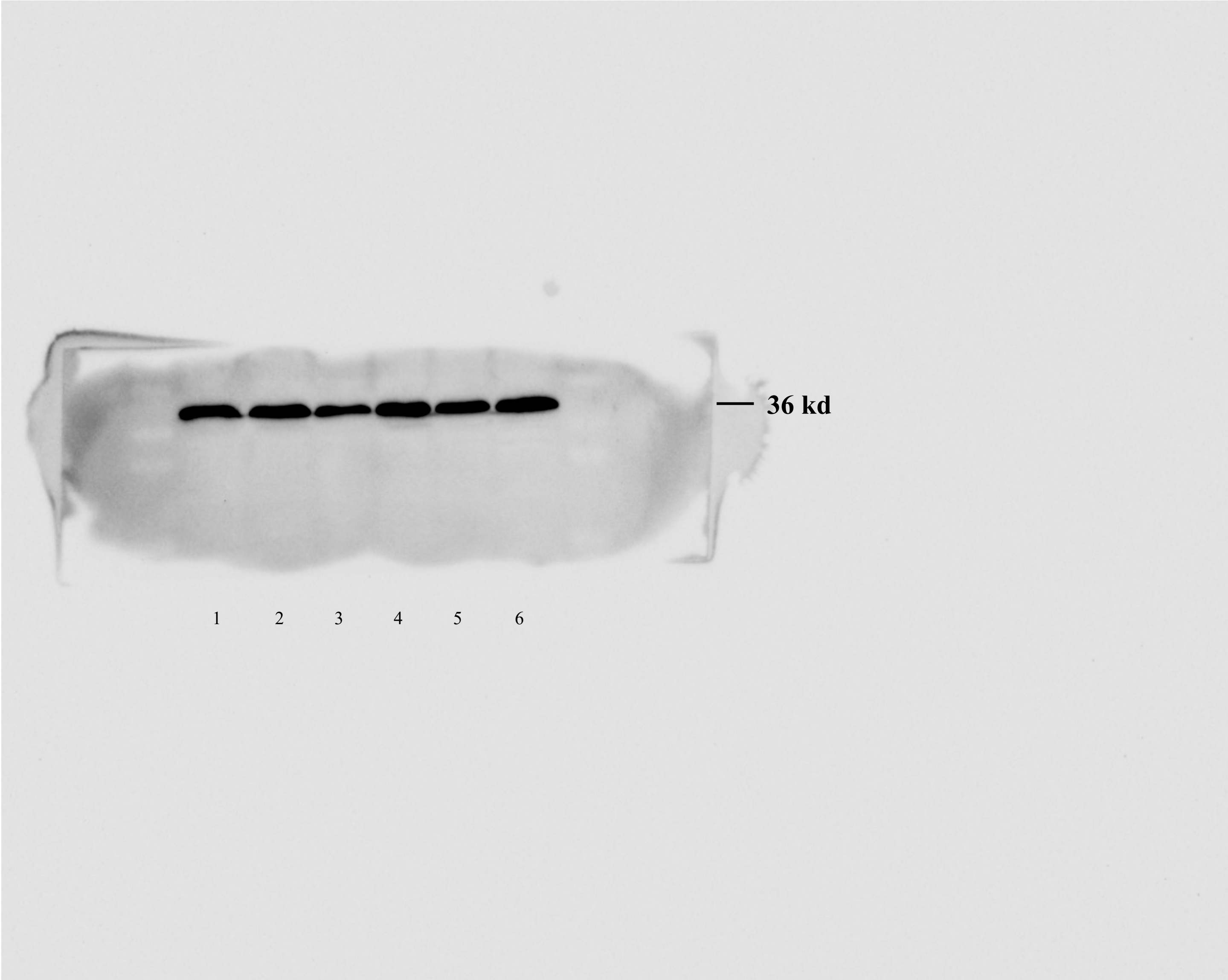

GAPDH

Figure S1C (siAFF4)

Sample legend:

Lane	1	2	3	4	5	6
Sample	Not mentioned in manuscript	Not mentioned in manuscript	Not mentioned in manuscript	NC	siAFF4	Not mentioned in manuscript

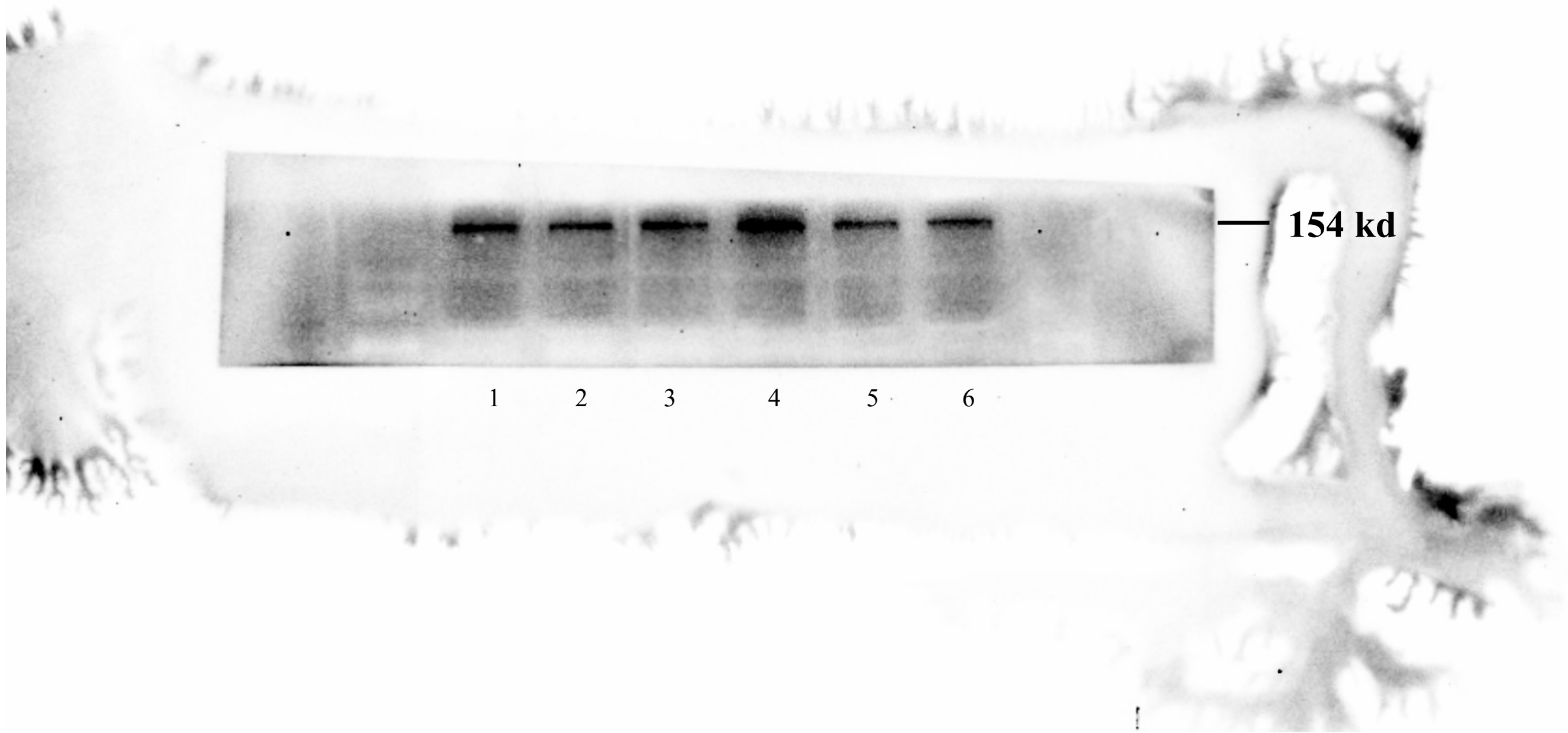

AFF4

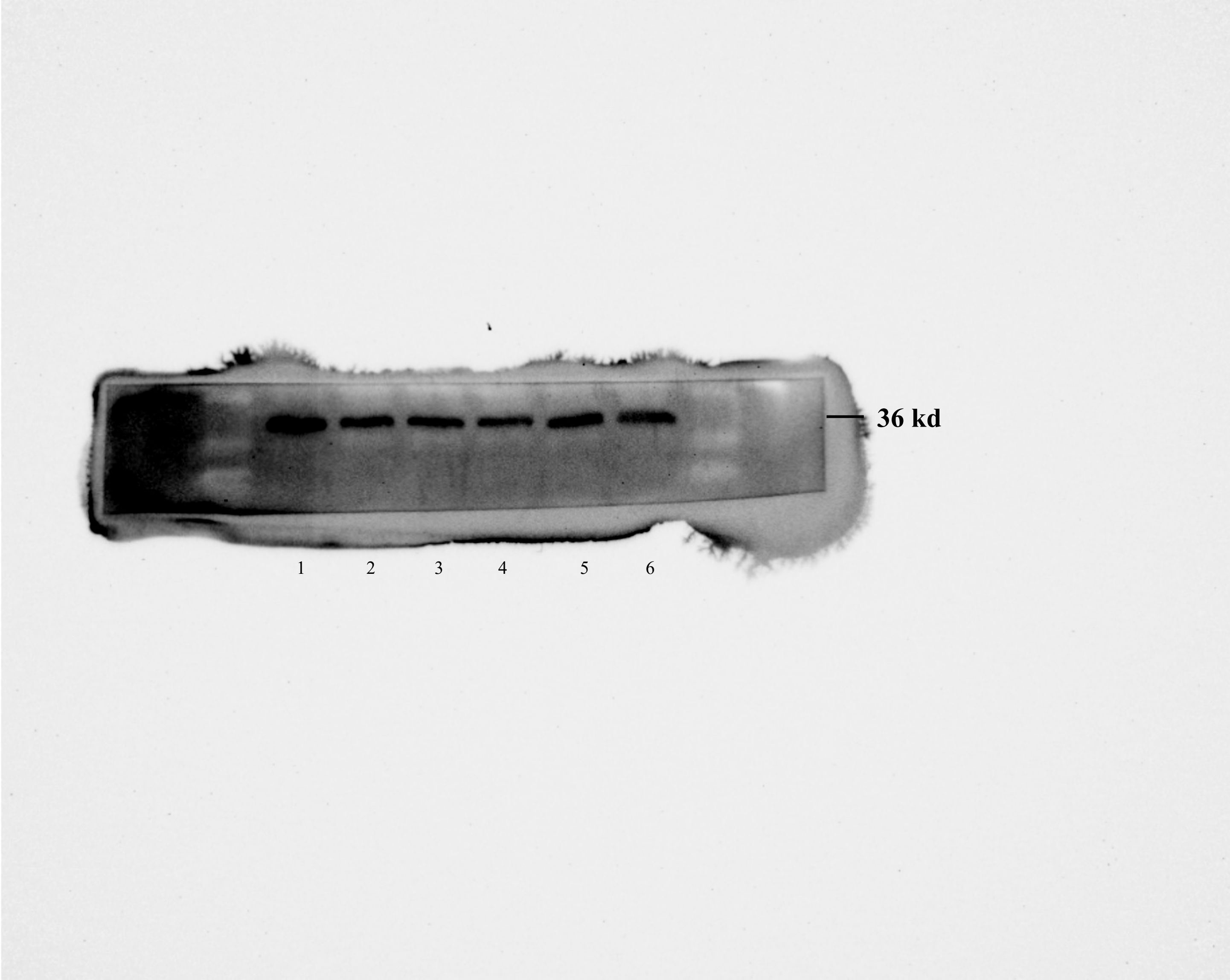

GAPDH

Figure S1C (siELL)

Sample legend:

Lane	1	2	3	4	5
Sample	Not mentioned in manuscript	Not mentioned in manuscript	NC	siELL	Not mentioned in manuscript

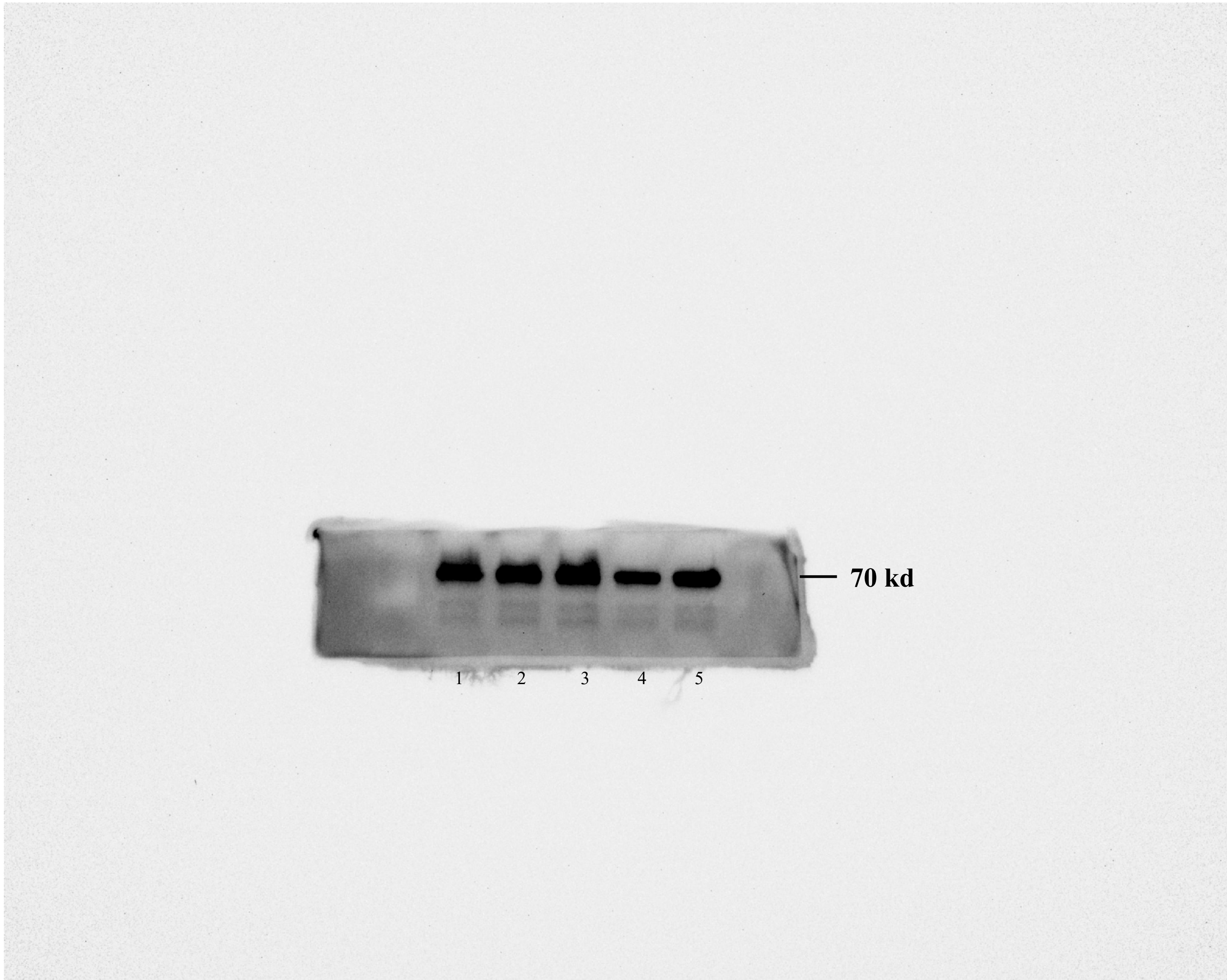

ELL

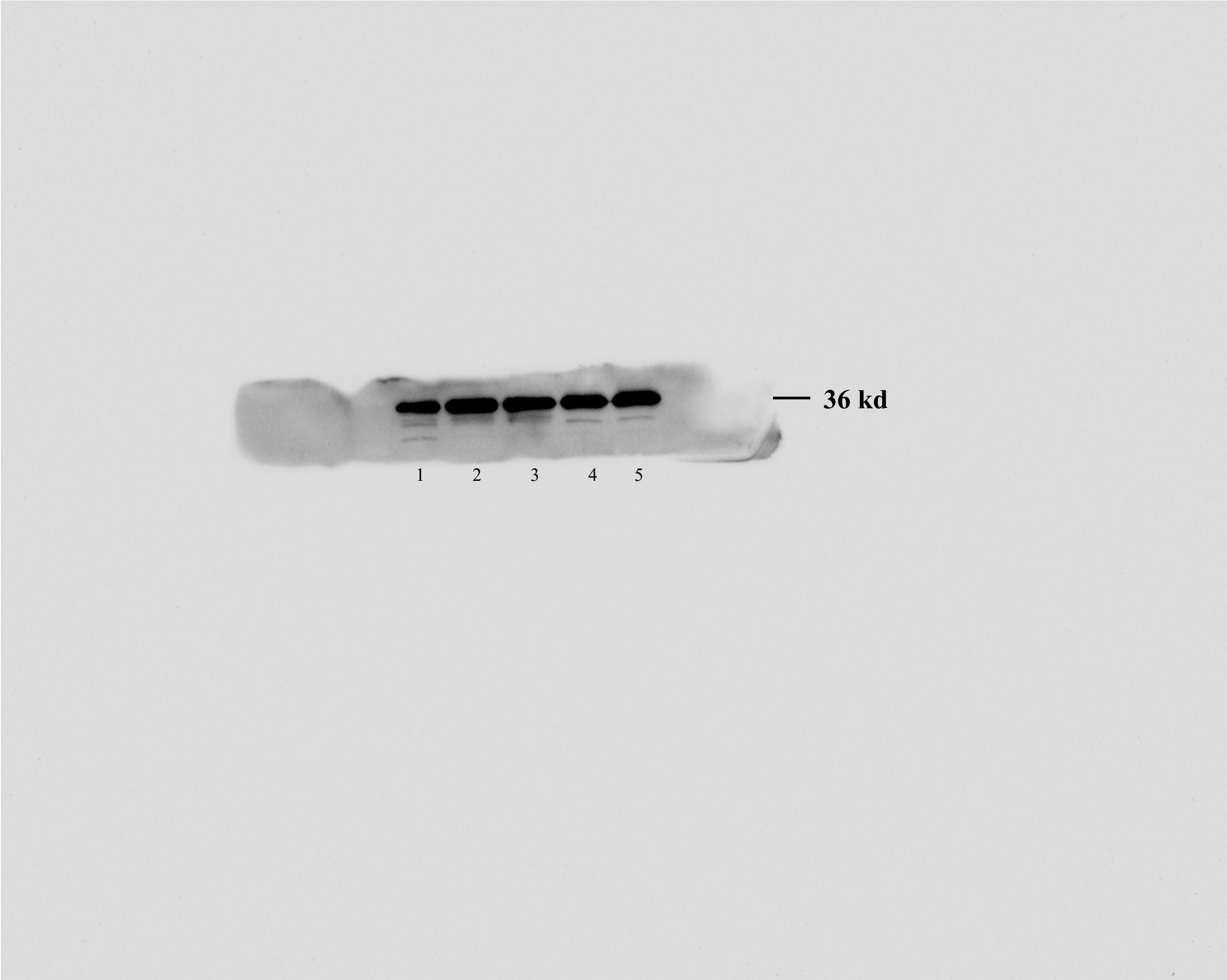

GAPDH

Figure S1E

Sample legend:

Lane	1	2	3	4	5
Sample	Not mentioned in manuscript	Not mentioned in manuscript	marker	NC	shELL

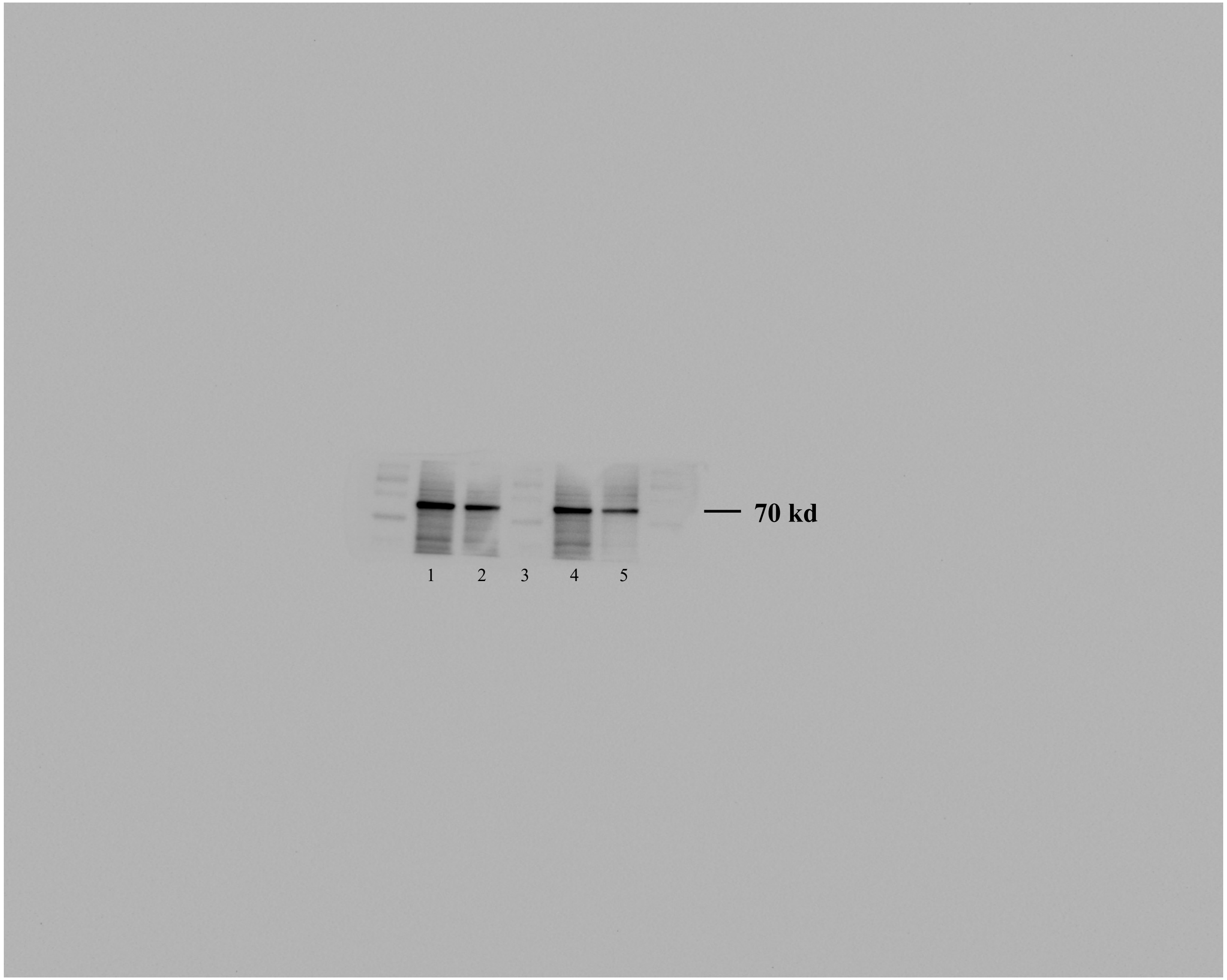

ELL

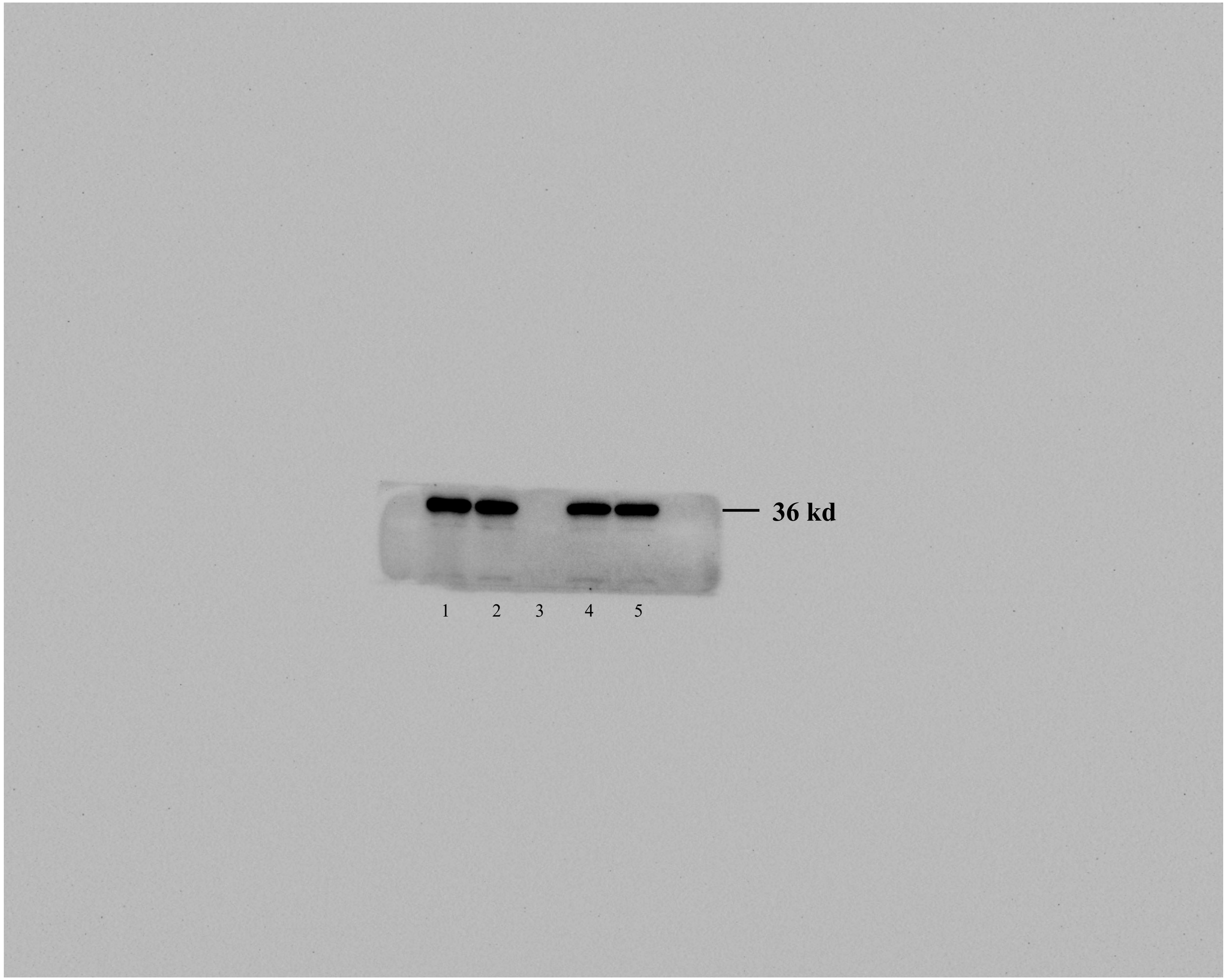

GAPDH

Figure S1G

Sample legend:

Lane	1	2
Sample	control	ELL OE

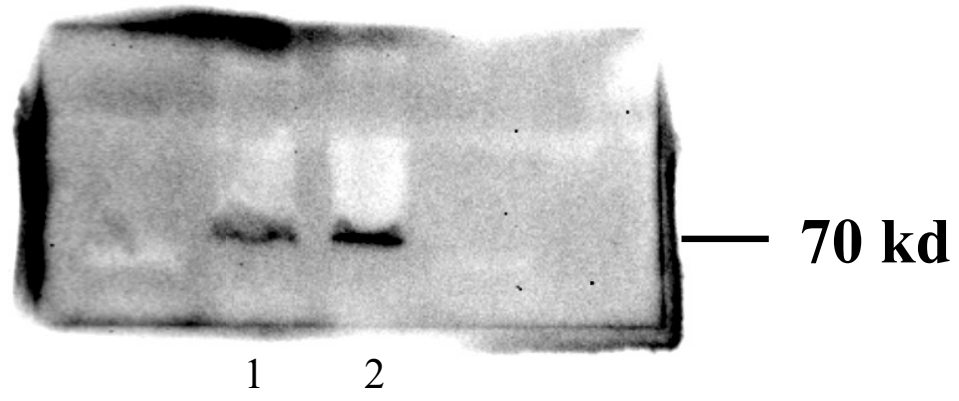

ELL

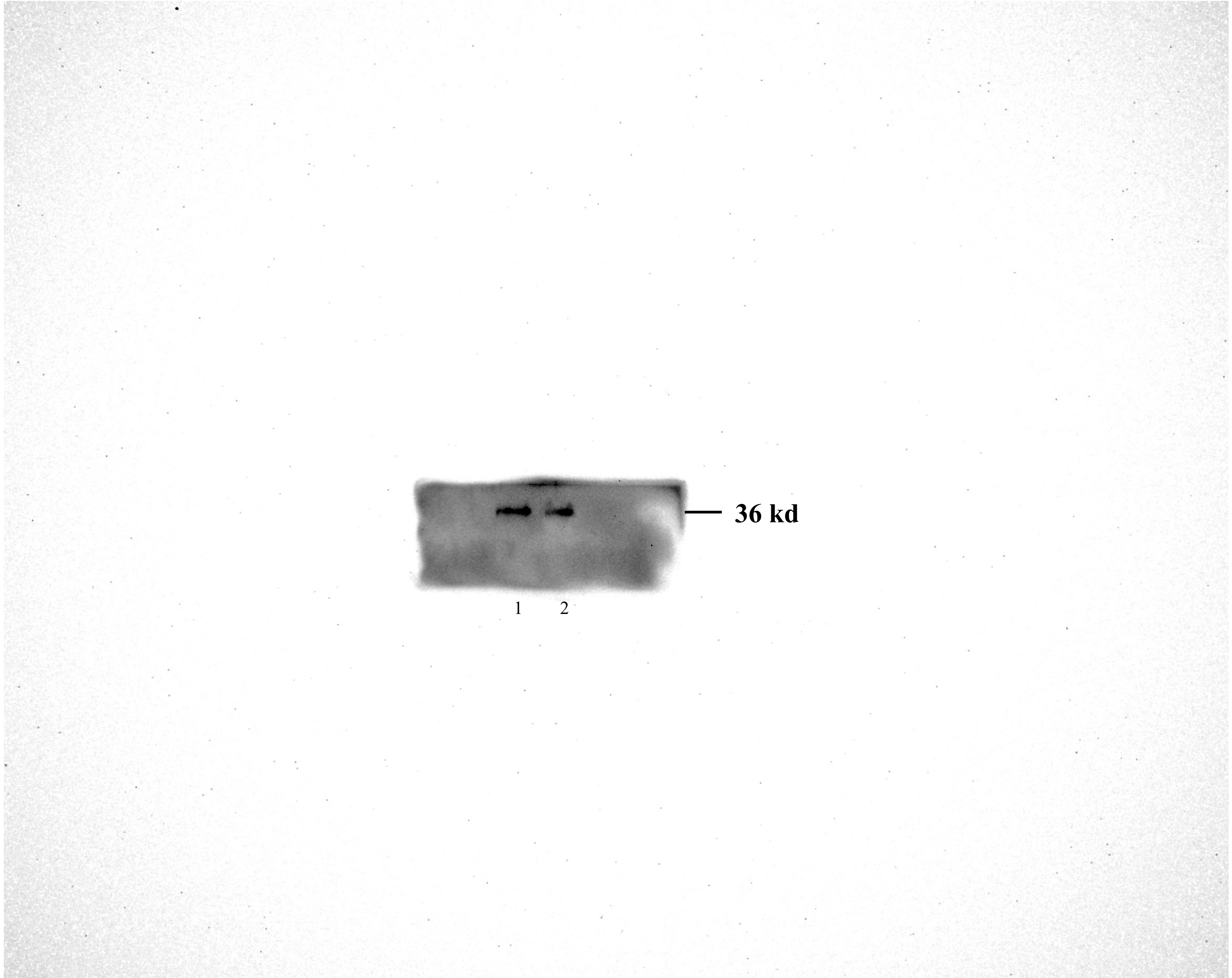

GAPDH

Figure S4A

Sample legend:

Lane	1	2	3	4	5
Sample	DMSO (Input)	KL-2 (Input)	DMSO (NELF-e IP)	KL-2 (NELF-e IP)	IgG

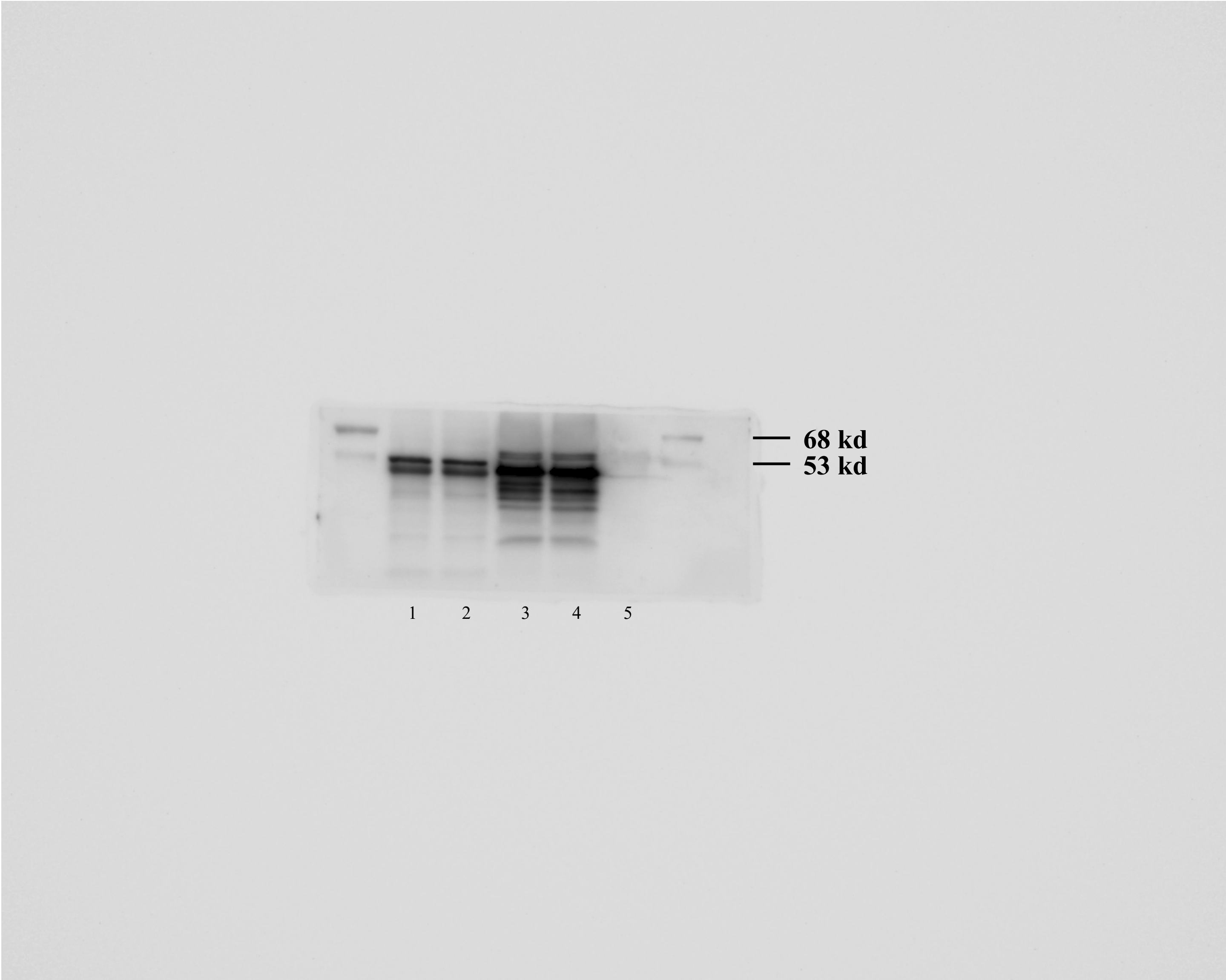

NELF-e

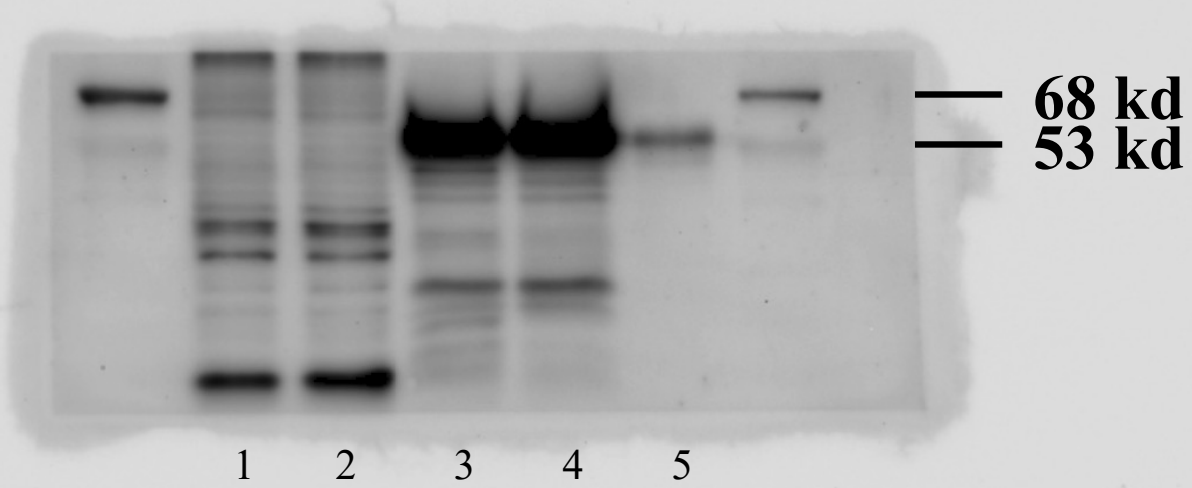

Phospho-(Ser/Thr) Phe

Figure S5G

Sample legend:

Lane	1	2	3	4
Sample	DMSO	KL-2	Not mentioned in manuscript	Not mentioned in manuscript

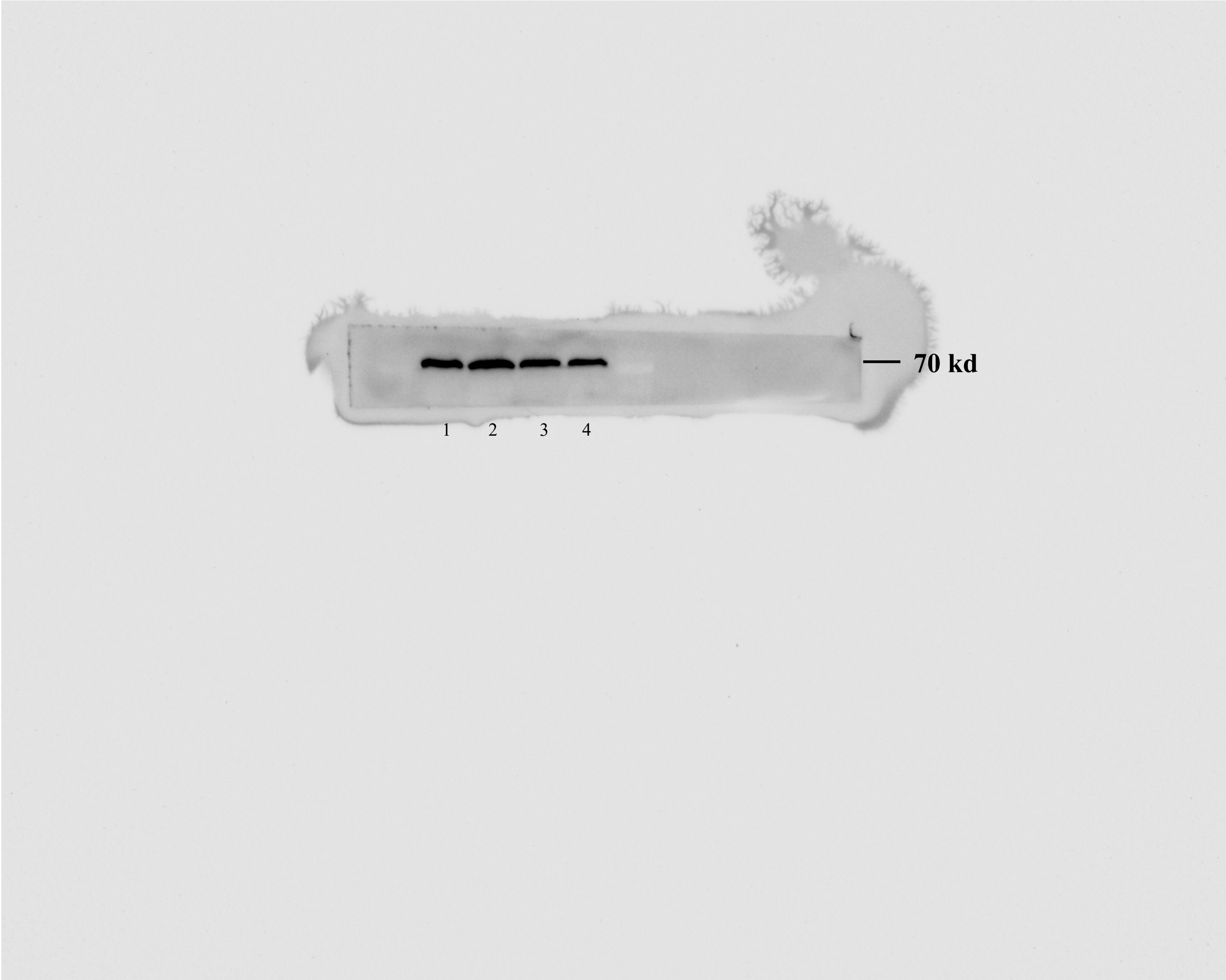

METTL3

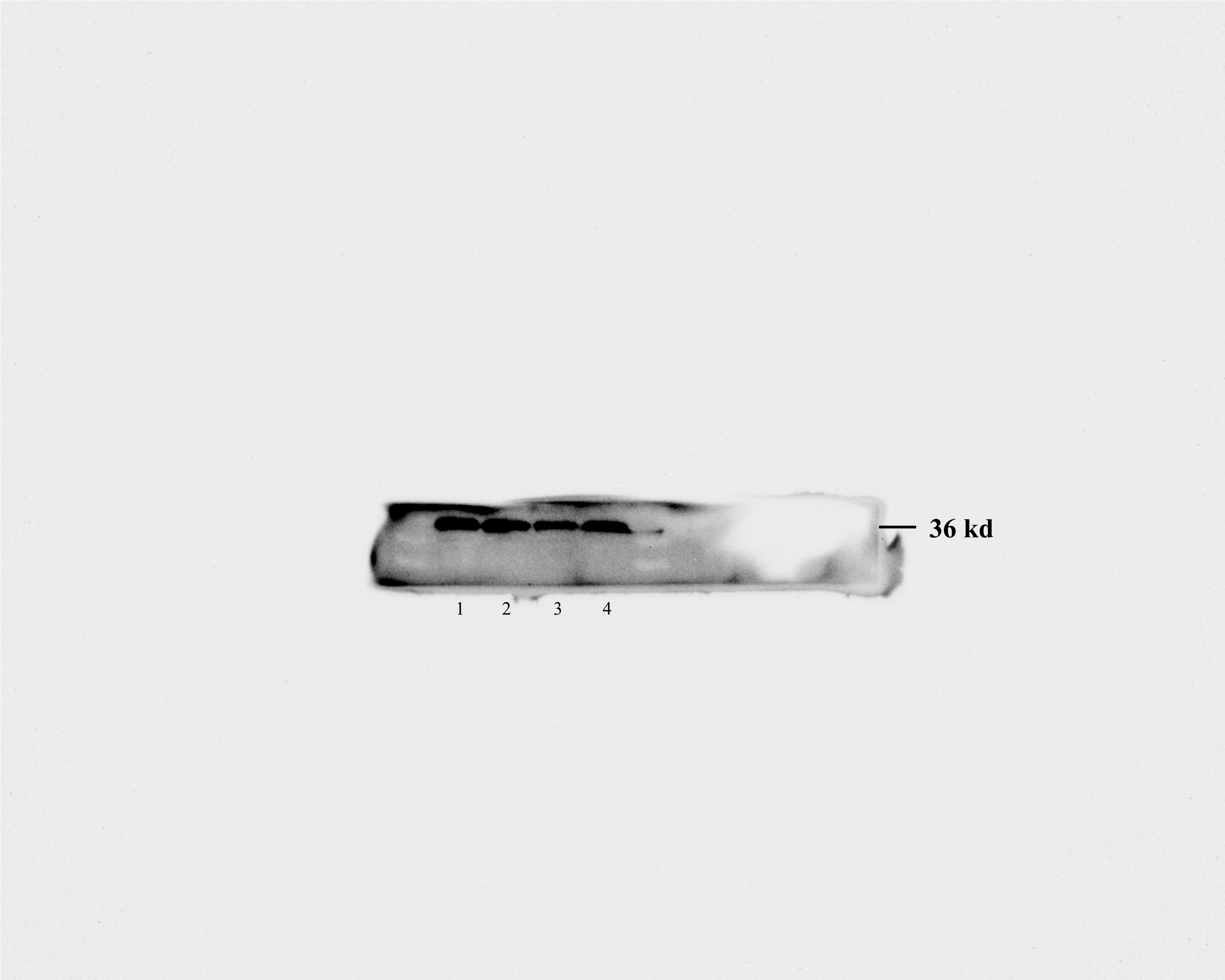

GAPDH

Figure S6A

Sample legend:

Lane	1	2	3	4
Sample	Not mentioned in manuscript	Not mentioned in manuscript	DMSO (400 ng)	STM2457 (400 ng)
	Not mentioned in manuscript	Not mentioned in manuscript	DMSO (200 ng)	STM2457 (200 ng)

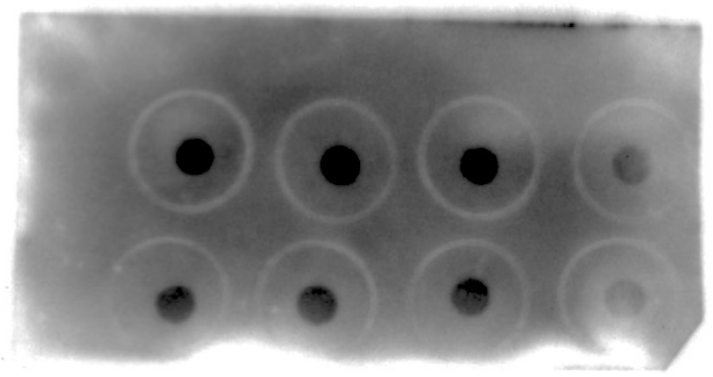

m^{6}A

Figure S6B

Sample legend:

Lane	1	2	3	4
Sample	DMSO	STM2457	Not mentioned in manuscript	Not mentioned in manuscript

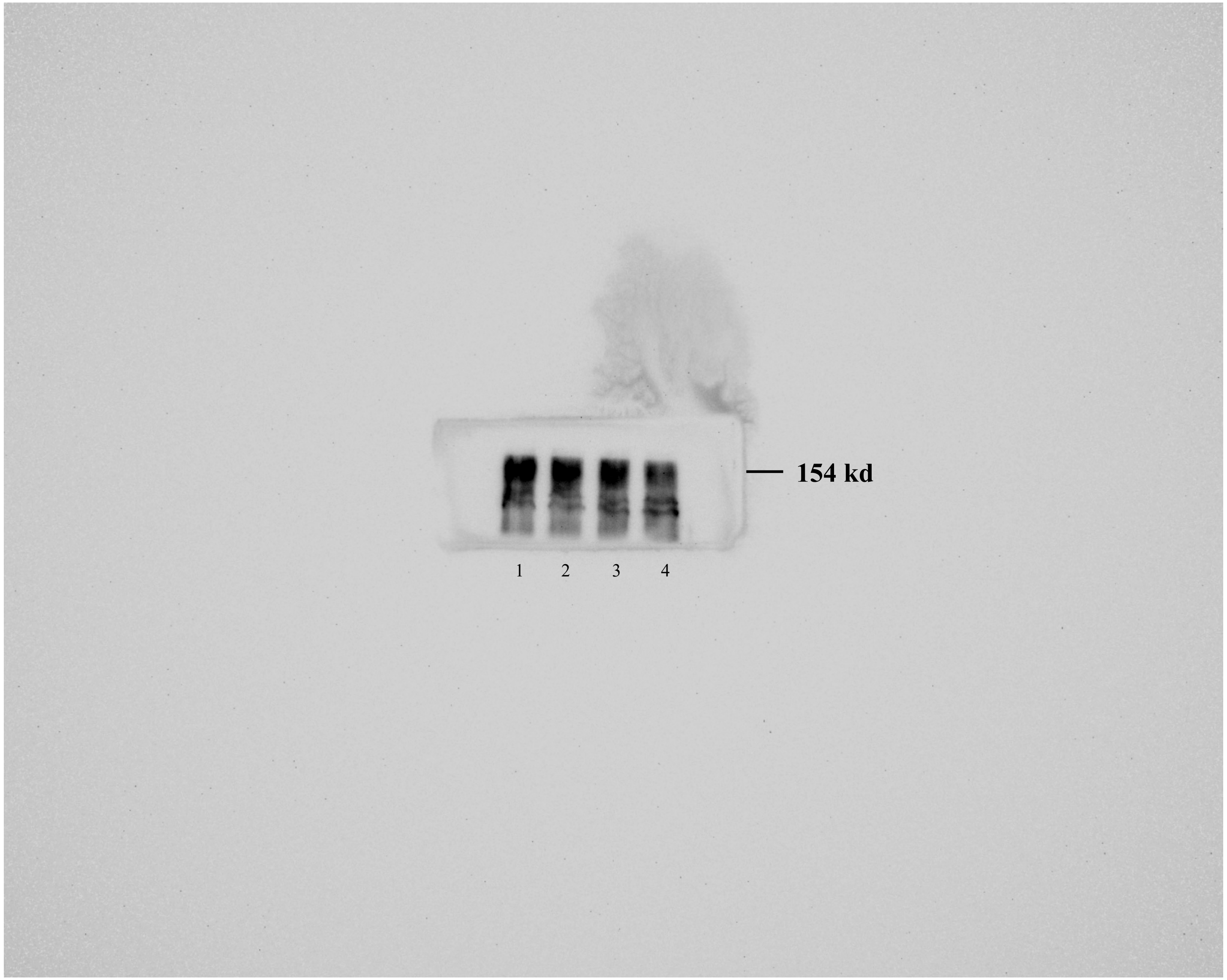

AFF4

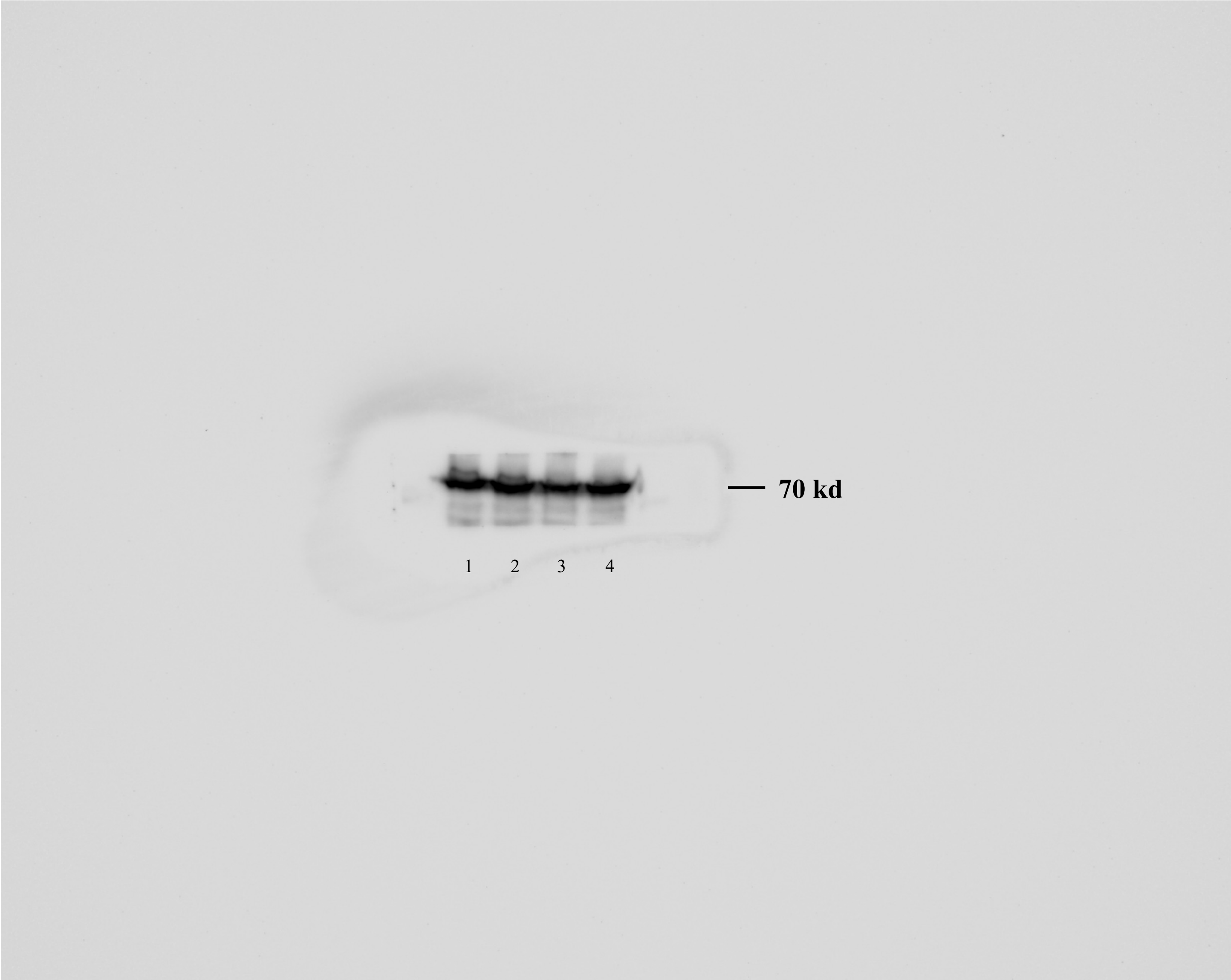

ELL

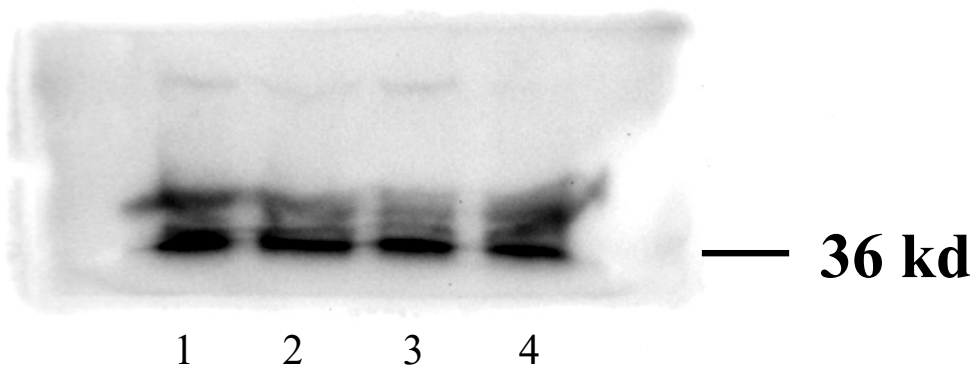

GAPDH

Figure S6D

Sample legend:

Lane	1	2
Sample	control	METTL3-OE

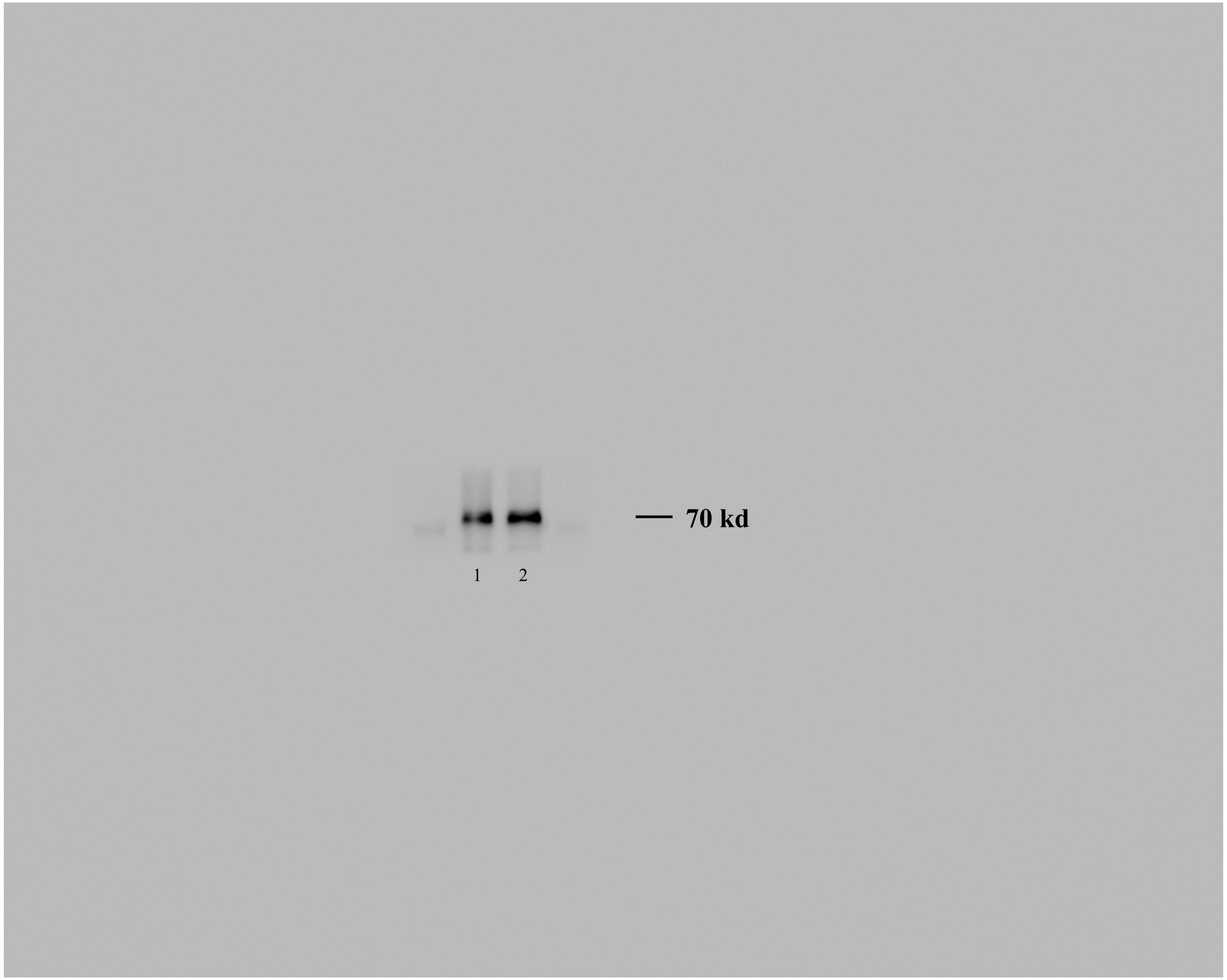

METTL3

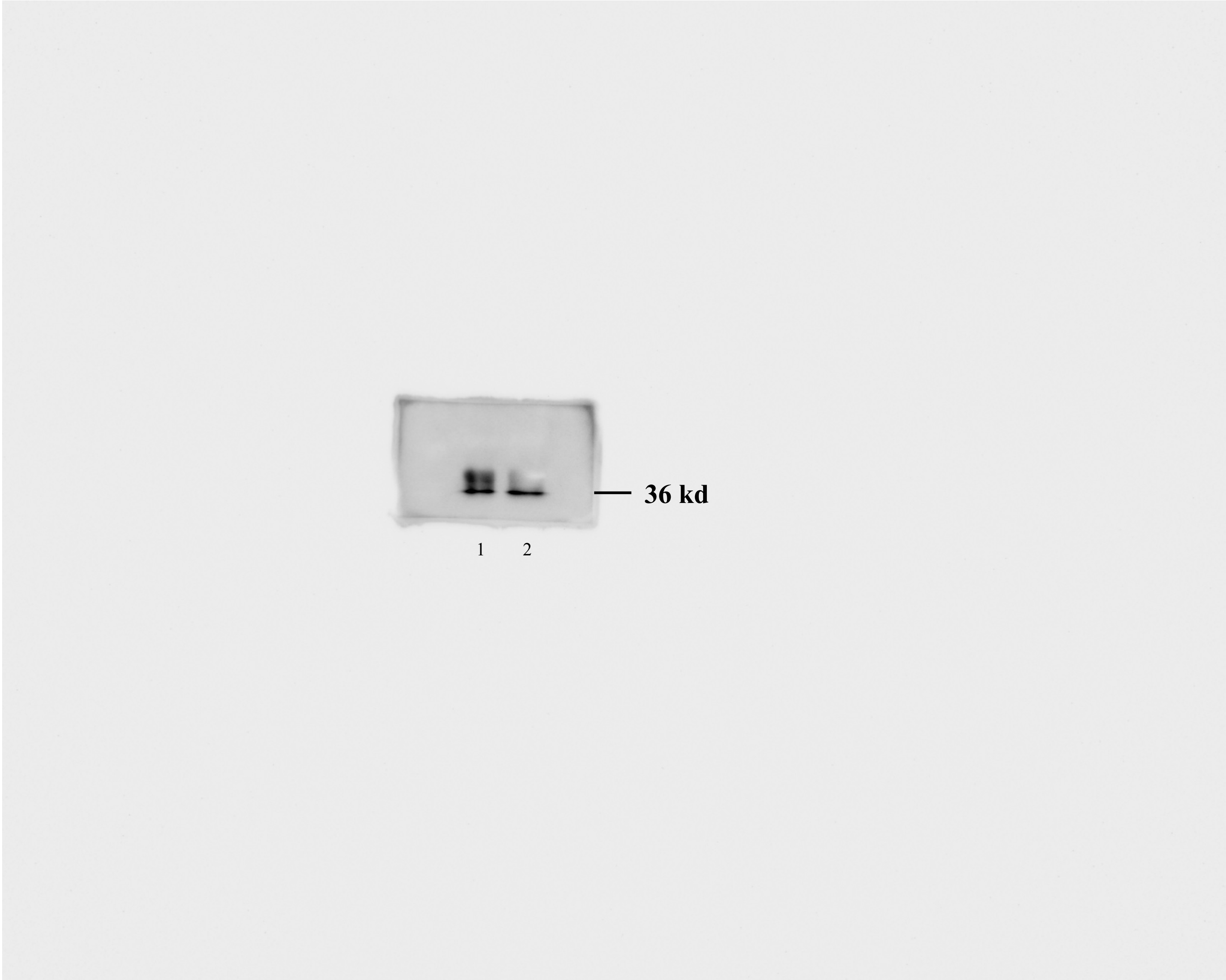

GAPDH

Figure S8D

Sample legend:

Lane	1	2	3	4	5	6	7	8
Sample	Not mentioned in manuscript	Not mentioned in manuscript	Not mentioned in manuscript	Not mentioned in manuscript	Not mentioned in manuscript	DMSO	KL-2	Not mentioned in manuscript

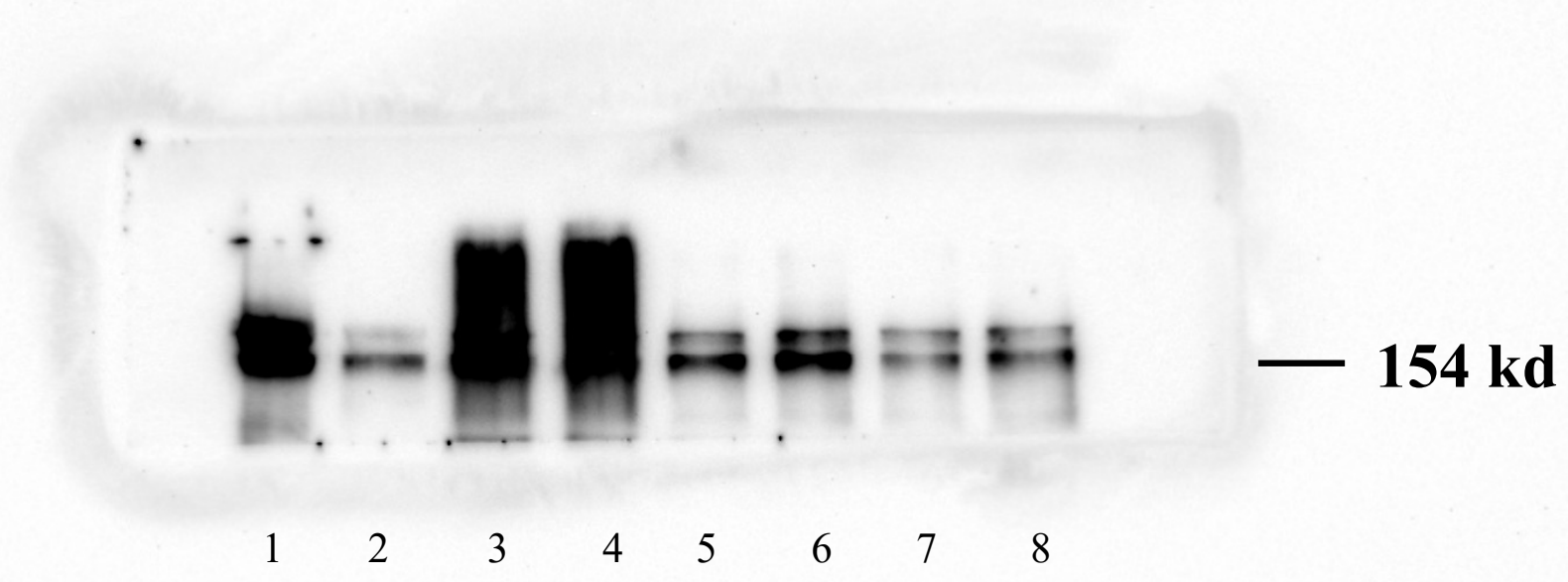

AFF4

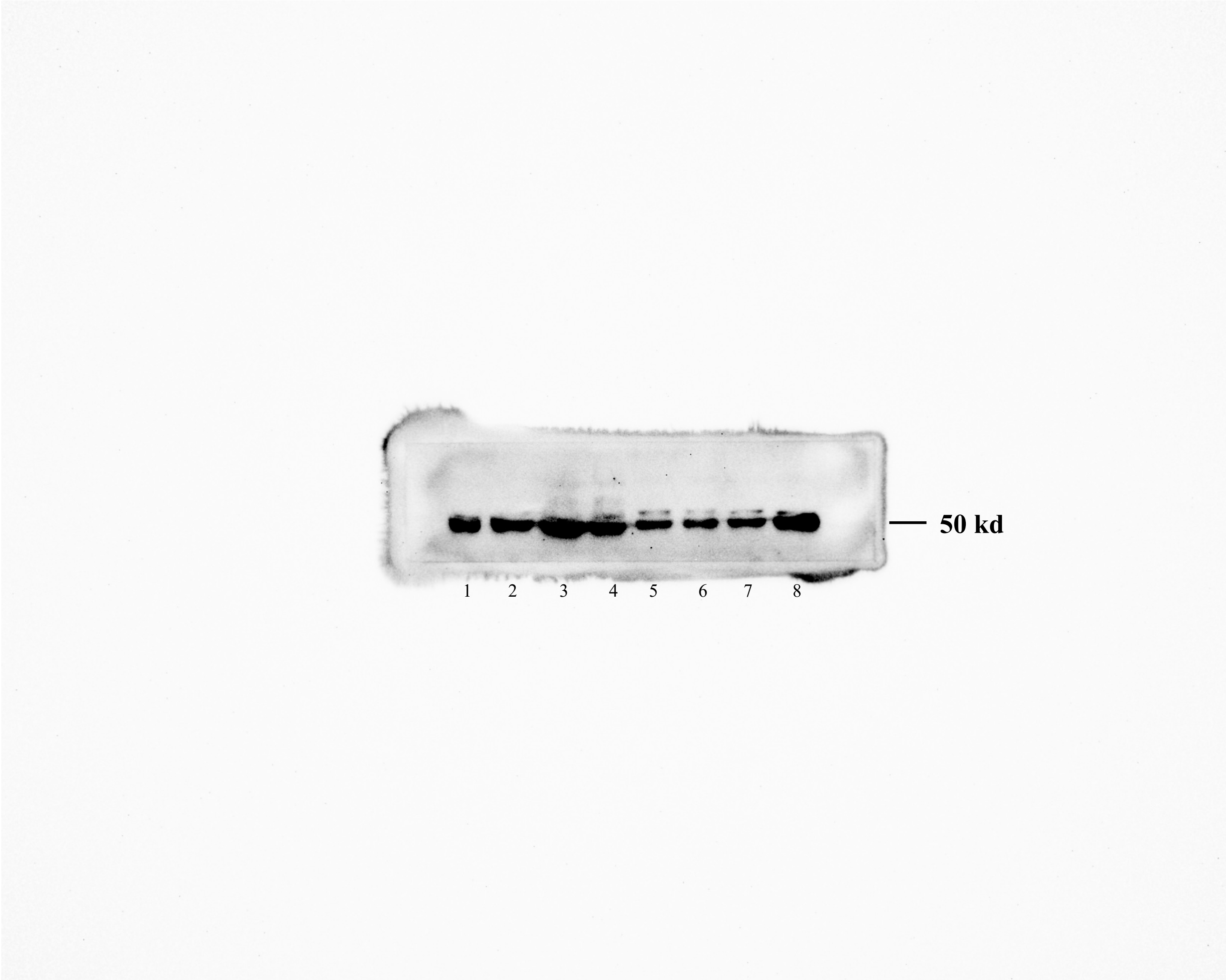

β-actin
